# Synthesis of Janus Bases for Targeting C‑G and U–U Pairs of CUG-RNA Repeats Associated with Myotonic Dystrophy Type 1

**DOI:** 10.1021/acsomega.5c09489

**Published:** 2025-10-06

**Authors:** Shivaji A. Thadke, J. Dinithi R. Perera, Savani W. Thrikawala, Danith H. Ly

**Affiliations:** Department of Chemistry and Institute for Bimolecular Design and Discovery (IBD), 6612 Carnegie Mellon University, 4400 Fifth Avenue, Pittsburgh, Pennsylvania 15213, United States

## Abstract

We report the synthesis of bifacial Janus bases E and K, along with their corresponding γ peptide nucleic acid monomers, designed to recognize C-G and U–U pairs within CUG-RNA repeats implicated in Myotonic Dystrophy Type 1. This study establishes the foundation for a fully programmable system of 16 Janus bases capable of recognizing all possible RNA base-pairs—both canonical and noncanonical—allowing precise manipulation of RNA structure and function. Importantly, beyond targeting CUG-repeats, this system holds a potential promise for addressing other triplet repeat expansions linked to over 35 neuromuscular and neurodegenerative disorders, thereby opening up new avenues for therapeutic intervention.

## Graphical abstract

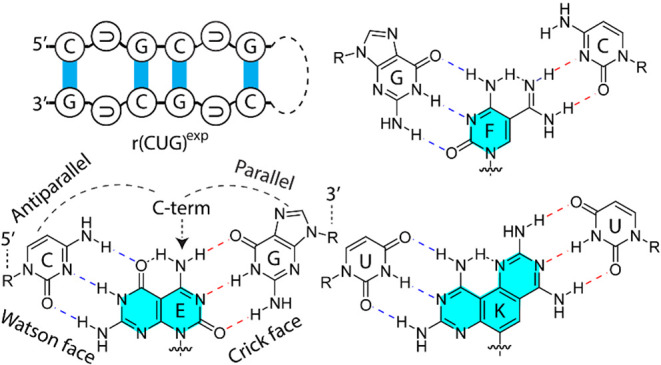

## Introduction

Ribonucleic acids (RNAs), despite being composed of just four chemical building blocks, can fold into intricate three-dimensional (3D) structures, rivaling proteins in diversity and complexity. This folding process is driven by the intramolecular self-organization of secondary motifs, guided by noncovalent interactions such as canonical and noncanonical base-pairing and van der Waals forces.^1^ While base-pairing simplifies sequence recognition, targeting RNA’s three-dimensional folds is far more challenging due to kinetic and thermodynamic constraints.^2^ Overcoming this hurdle requires prioritizing affinity over specificity, which almost inevitably leads to greater off-target effects.

This realization has sparked a growing interest in designing small molecules to target RNA, given its critical role in disease. However, unlike proteins, which often have well-defined binding pockets, RNA typically lacks such structures, making rationale design more difficult.^3,4^ To obviate this design bottleneck, researchers frequently turn to high-throughput screening to identify lead compounds.^5,6^ While many have been discovered,^7–9^ the issues of specificity and selectivity remain major hurdles. Improving precision in RNA targeting could enhance potency while minimizing off-target effects and toxicity—key to developing safe and effective therapies.^10,11^

Here, we report the synthesis of bifacial Janus bases G/C and A/A, and the corresponding γ peptide nucleic acid (γPNA) building blocks, designed to target C-G and U–U pairs within CUG-RNA repeats associated with Myotonic Dystrophy Type 1 (DM1). At just three units in length, these ligands resemble small molecules more than oligonucleotides. However, unlike typical small molecules, they achieve high specificity and selectivity through bifacial hydrogen-bonding with opposing nucleobases in the RNA loop—effectively filling a molecular “pothole”.

DM1 is a priority target, as it is a debilitating neuromuscular disorder for which there is no effective treatment.^12^ As the most common adult muscular dystrophy, DM1 affects 1 in 8000 people worldwide. It arises from an expanded CTG-repeat in the 3′-UTR of the *DMPK* gene, with a normal range of 5 to 35 repeats to a pathogenic range of >2500, with the severity of the disease directly correlated with the size of the repeat expansion and inversely related to the age of onset.^13^ Upon transcription, the expanded CUG-repeats adopt stable hairpin structures that sequester muscleblind-like protein 1 (MBNL1) and related RNA-splicing factors, disrupting RNA splicing and causing nuclear foci accumulation.^14–16^ This mis-splicing affects key genes like Clc-1 and IR, contributing to muscle hyperexcitability and insulin resistance.^17^ Since DM1 is primarily a disorder of misregulated RNA splicing, restoring MBNL1 function and that of other members of this protein family could halt or even reverse disease progression.^18^ While various treatment strategies, including small molecules,^19,20^ antisense technology,^18^ and genome editing,^21^ have been explored, none has been approved. Our approach provides an alternative by combining the appealing attributes of small molecules and antisense oligonucleotides to enhance specificity and selectivity.

## Results and Discussion

### Rationale

We previously reported the synthesis of first-generation Janus bases G/C (e) and C/G (F) (Chart 1) to target C-G and G-C pairs in DNA or RNA, each forming five hydrogen-bonds.^22,23^ When incorporated into a right-handed γPNA backbone and assembled into short oligomers (3–6 units in length), both molecular modeling and experimental findings confirmed that they engaged RNA targets as predicted through directional hydrogen-bonding. This recognition is enabled by a conformationally preorganized backbone.^24,25^ To further improve binding and expand sequence recognition for targeting CUG-RNA repeats, increasing hydrogen-bonding and base-stacking interactions, along with developing a novel Janus base A/A (K) for recognizing U–U pairs, is a logical next step.

**Chart 1:**
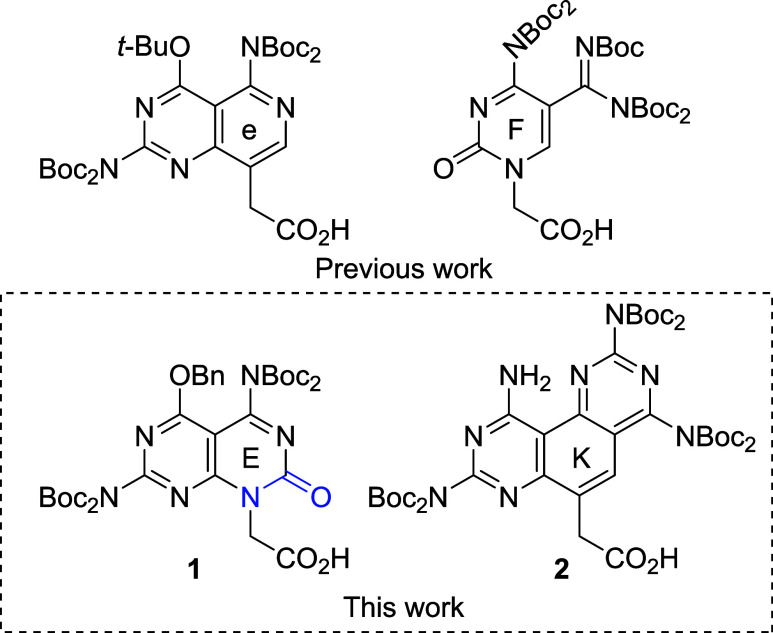
Chemical Structures of Carboxymethyl Janus Bases G/C (e, E), C/G (F), and U/U (K)

To achieve this, we incorporated an amide moiety into the primary ring of e, creating E (Chart 1), which increases hydrogen-bonding from five to six. Notably, similar compounds have been reported by He,^26^ Marchan,^27^ and Tor^28^ and Perrin^29^ for targeting C-G, U-G, and A-T­(U) pairs, respectively. However, without the necessary protecting groups, linkers, and preorganized backbone, these compounds are unsuitable for solid phase peptide synthesis (SPPS) and incapable of binding structured RNA. Incorporating the amide functionality not only strengthens hydrogen-bonding but also likely enhances base-stacking interactions.

Redesigning F, however, presents more significant challenges. While adding a second ring would enhance base-stacking and enable hexameric hydrogen-bonding, it also promotes a nonpreferred tautomeric form (Figure S1, Supporting Information) and is prone to acid degradation in SPPS. Due to these limitations, we deferred modifications for later study. To maintain structural uniformity for all 16 Janus bases, we designed a tricyclic ring system with a 2,4-diaminopyridine feature on both faces of K. This structure forms six hydrogen-bonds with U–U pairs while its expanded ring system enhances base-stacking interactions, improving stability, specificity, and selectivity. These improvements are essential for designing triplet ligands that targets CUG-RNA hairpins with high affinity, specificity, selectivity, and cooperativity, enabling the effective differentiation of expanded repeats from wild-type sequences. It is worth noting that while melamine has been used for U–U recognition,^19,30^ it is incompatible with the current design to enable modular nucleic acid recognition.

### Synthesis of Carboxymethyl Janus Base E

We adopted a strategy similar to that reported by Marchan^27^ and Fenniri^31^ for constructing the primary ring (Scheme 1). Starting with 2-amino-4,6-dichloropyrimidine-5-carbaldehyde, two sequential nucleophilic aromatic substitutions yielded intermediate **4**, featuring a linker and benzyl-protection, which was later converted into a carbonyl in the cleavage step of SPPS. Conversion of aldehyde to nitrile followed a two-step process, amination followed by dehydration to afford **6**. We attempted a recently reported one-pot reaction but was unsuccessful,^32^ likely due to the electron-donating groups in the *ortho* positions. The exocyclic amine was temporarily protected with *N*,*N*′-dimethylformamide dimethyl acetal (DMF-DMA) before undergoing cyclization with trichloroacetyl isocyanate in the presence of methanolic ammonia, which simultaneously removed DMF-DMA, yielding the bicyclic intermediate **8**. An attempt to install a single Boc on each exocyclic amine using CDI and *t*-butyl alcohol was unsuccessful. Instead, a standard DMAP/Boc_2_O protection protocol was used, resulting in two Boc groups per amine. Finally, allylic oxidation produced the carboxymethyl Janus base **E**.

**Scheme 1:**
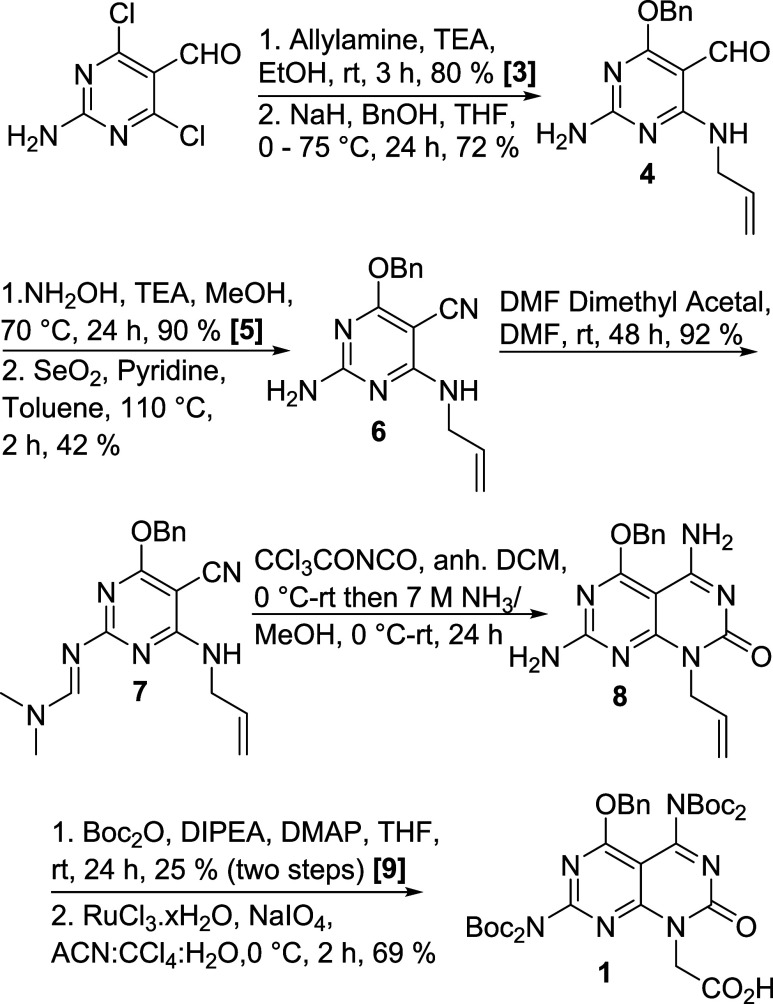


### Synthesis of Carboxymethyl Janus Base K

The synthetic route for K is outlined in Scheme 2, beginning with 2,4-difluoroisophthalonitrile. Lithiation occurred exclusively at the *ortho* position due to the unique fluorine effect.^33^ The conversion of carboxyl to nitrile followed established protocols. Cyclization was carried out at elevated temperatures, and the resulting mixture was used directly in the bromination step after aqueous wash to remove polar byproducts, without purification. The formation and bromination of the tricyclic system can be easily traced by the appearance and disappearance of intense blue fluorescence. Boc-protection and Stille coupling with freshly prepared ethyl 2-(tributylstannyl)­acetate yielded the ester intermediate **16**. Saponification followed by neutralization yielded a white precipitate, which was filtered and dried to give compound **2** as a major compound. The minor product contained seven Boc-groups.

**Scheme 2:**
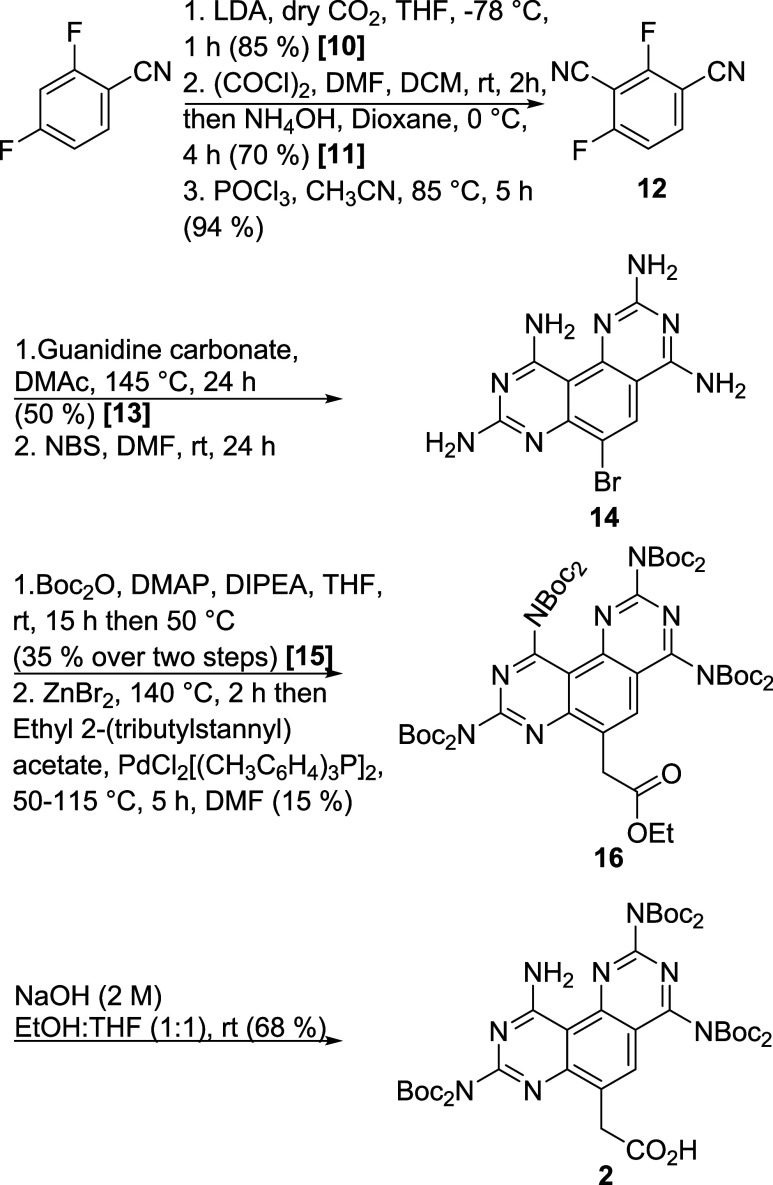


### Synthesis of γPNA Building Blocks Containing E and K

Compound **1** and **2** were coupled to Fmoc-protected miniPEG-γPNA (MPγPNA) backbone 17 (Scheme 3).^34^ The reaction was conducted at elevated temperatures to enhance coupling efficiency, overcoming steric hindrance from the nucleobases and backbone. Removal of the Alloc protecting group produced the final building blocks **19** and **20**, which were then used for oligomer synthesis via SPPS.

**Scheme 3:**
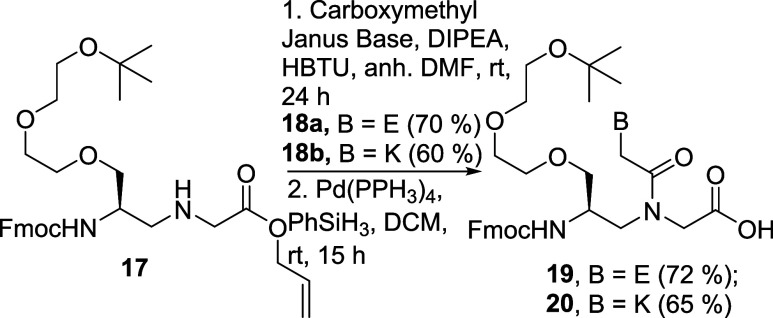


Triplet ligands composed of E, F, and K were successfully synthesized, purified, and characterized. They demonstrated high specificity, selectivity, and cooperativity for their targets, effectively distinguishing expanded CUG-RNA repeats from the wild-type. These results are unpublished and will be reported in due course.

## Conclusions

This study lays the foundation for an alternative RNA-targeting strategy that merges the advantages of small molecules and antisense oligonucleotides. By integrating bifacial nucleobases with a preorganized chiral γPNA backbone, we introduce a modular approach for precise RNA recognition. Our work is the first step toward constructing a complete set of 16 Janus bases capable of binding any RNA base-pair combinations, enabling precision control over RNA structure and function. Unlike previous systems, these bases can be rationally designed for tailored RNA targeting, paving the way for future therapeutic and biotechnological applications.

## Experimental Section

All starting materials and chemicals were obtained from commercial suppliers and used without further purification unless specified. Reactions were conducted under a nitrogen atmosphere with dried solvents under anhydrous conditions, unless otherwise noted. Solvent removal in vacuo refers to distillation using a rotary evaporator connected to a high-vacuum pump, and products obtained as solids, syrups, or liquids were dried under high vacuum.

Analytical thin-layer chromatography (TLC) was performed on precoated silica gel plates (60 F_254_, 0.25 mm thickness), with visualization using ultraviolet (UV) light or staining with ninhydrin, iodine, or heat as developing agents. NMR spectra were recorded on 300 or 500 MHz spectrometers using CDCl_3_ or DMSO-*d*_6_ as solvents, with TMS as an internal standard. The following abbreviations were used to denote multiplicities in NMR spectroscopy: s (singlet), d (doublet), t (triplet), q (quartet), m (multiplet), dd (doublet of doublets), tt (triplet of triplets), br (broad), and others. Common solvent impurities were labeled as EA (ethyl acetate), DMF (dimethylformamide), DCM (dichloromethane), etc. High-resolution mass spectrometry (HRMS) was conducted using an ESI-TOF mass analyzer and DART analyzer.

### 4-(Allylamino)-2-amino-6-chloropyrimidine-5-carbaldehyde (**3**)

2-Amino-4,6-dichloropyrimidine-5-carbaldehyde (25.0 g, 128.35 mmol) was dissolved in EtOH (600 mL). To this solution, triethylamine (19.8 mL, 141.18 mmol) was added, followed by the dropwise addition of allylamine (9.83 mL, 128.35 mmol). The reaction mixture was stirred at room temperature for 3 h, leading to precipitation of the product. The solid was filtered, washed with cold EtOH, and dried, yielding an off-white solid (21.8 g, 80%). *R*_f_ = 0.4 (30% EA/hexane). The compound was used directly in the next step without further purification. ^1^H NMR (500.13 MHz, CDCl_3_) δ 9.91 (s, 1H), 9.19 (t, 1H), 7.72 (s, 1H), 7.63 (s, 1H), 5.93 (ddt, *J* = 17.2, 10.5, 5.3 Hz, 1H), 5.13–5.19 (m, 2H), 4.09 (tt, *J* = 5.5, 1.7 Hz, 2H). ^13^C­{^1^H} NMR (125.77 MHz, CDCl_3_) δ 187.9, 165.8, 162.8, 162.4, 134.9, 116.2, 42.4; HRMS (ESI) *m*/*z*: [M + H]^+^ Calcd for C_8_H_9_ClN_4_OH 213.0543; Found 213.0539.

### 4-(Allylamino)-2-amino-6-(benzyloxy)­pyrimidine-5-carbaldehyde (**4**)

BnOH (11.26 mL, 108.17 mmol) was added to a solution of NaH (4.76 g, 118.98 mmol) in dry tetrahydrofuran (THF) (160 mL) and stirred for 1 h. The reaction mixture was then cooled to 0 °C in a salt-ice–water bath, and compound **3** (11.50 g, 54.08 mmol), dissolved in dry THF (320 mL), was added dropwise. Upon completion of the addition, the reaction was warmed to rt and refluxed at 75 °C in an oil bath under N_2_ for 24 h. After cooling, the THF layer was extracted with H_2_O (300 mL) and brine (100 mL). The organic layer was dried over Na_2_SO_4_, concentrated, and subjected to flash chromatography (5–30% EA/hexane) on silica gel. The desired compound (11.1 g, 72%) was obtained as a yellow solid. *R*_f_ = 0.45 (30% EA/hexane). ^1^H NMR (500.13 MHz, CDCl_3_) δ 9.85 (s, 1H), 9.17 (s, 1H), 7.33–7.47 (m, 7H), 5.92 (ddt, *J* = 17.2, 10.5, 5.3 Hz, 1H), 5.41 (s, 2H), 5.12–5.17 (m, 2H), 4.08 (d, *J* = 1.8 Hz, 2H). ^13^C­{^1^H} NMR (125.77 MHz, CDCl_3_) δ 184.8, 171.8, 164.2, 163.5, 137.0, 135.5, 128.9, 128.3, 128.4, 115.8, 92.2, 67.3, 42.3; HRMS (ESI) *m*/*z*: [M + H]^+^ Calcd for C_15_H_16_N_4_O_2_H 285.1352; Found 285.1346.

### 4-(Allylamino)-2-amino-6-(benzyloxy)­pyrimidine-5-carbaldehyde Oxime (**5**)

Compound **4** (10 g, 35.17 mmol) was dissolved in MeOH (175 mL). To this solution, triethylamine (19.81 mL, 140.69 mmol) was added, followed by hydroxylamine hydrochloride (9.90 g, 140.69 mmol). The reaction mixture was refluxed at 70 °C in an oil bath under a nitrogen atmosphere for 24 h. After cooling, MeOH was evaporated, yielding an off-white solid residue. The residue was dissolved in EA (200 mL), washed with H_2_O (150 mL) and brine (100 mL), dried over Na_2_SO_4_, and evaporated to dryness. The crude pale-yellow solid (9.5 g, 90%) was used directly in the next step without further purification. *R*_f_ = 0.40 (40% EA/hexane). ^1^H NMR (500.13 MHz, DMSO-*d*_6_) δ 10.59 (s, 1H), 8.25 (s, 1H), 8.06 (t, *J* = 5.6 Hz, 1H), 7.39–7.42 (m, 5H), 6.56 (s, 2H), 5.94 (ddt, *J* = 17.2, 10.3, 5.1 Hz, 1H), 5.33 (s, 2H), 5.10–5.20 (m, 2H), 4.10 (tt, *J* = 5.3, 1.8 Hz, 2H). ^13^C­{^1^H} NMR (125.77 MHz, DMSO-*d*_6_) δ 167.0, 161.7, 161.0, 144.2, 137.1, 135.5, 128.4, 127.8, 127.8, 115.2, 83.0, 66.6, 42.3; HRMS (ESI) *m*/*z*: [M + H]^+^ Calcd for C_15_H_17_N_5_O_2_H 300.1460; Found 300.1456.

### 4-(Allylamino)-2-amino-6-(benzyloxy)­pyrimidine-5-carbonitrile (**6**)

Compound **5** (6 g, 20.04 mmol) was suspended in toluene (500 mL) and heated to 30 °C in an oil bath for 15 min to form a suspension. Pyridine (2.1 mL) was then added, followed by selenium dioxide (2.27 g, 20.04 mmol). The reaction mixture was heated to 110 °C in the same oil bath and stirred for 3 h. Upon completion, the supernatant was decanted into a separate flask, evaporated, and purified by flash chromatography (10–50% EA/hexane) on silica gel, yielding the desired pale-yellow solid (2.37 g, 42%). *R*_f_ = 0.45 (40% EA/hexane); ^1^H NMR (500.13 MHz, DMSO-*d*_6_) δ 7.34–7.44 (m, 6H), 7.07 (s, 2H), 5.87 (ddt, *J* = 17.3, 10.3, 5.1 Hz, 1H), 5.37 (s, 2H), 5.04–5.11 (m, 2H), 3.95–3.97 (m, 2H). ^13^C­{^1^H} NMR (125.77 MHz, DMSO-*d*_6_) δ 170.6, 163.9, 162.8, 136.4, 135.6, 128.4, 128.0, 128.0, 115.9, 115.0, 67.0, 63.1, 42.4; HRMS (ESI) *m*/*z*: [M + H]^+^ Calcd for C_15_H_15_N_5_OH 282.1355; Found 282.1352.

### *N*′-(4-(Allylamino)-6-(benzyloxy)­pyrimidine-5-cyanopyrimidin-2-yl)-*N*,*N*-dimethylformimidamide (**7**)

Compound **6** (20 g, 71.09 mmol) was dissolved in dry DMF (475 mL), followed by the addition of 1,1-diethoxy-*N*,*N*-dimethylmethanamine (31.73 mL, 177.73 mmol). The mixture was stirred under a nitrogen atmosphere for 24 h. After completion, DMF was evaporated, yielding a yellow solid residue. The solid was then stirred in cold diethyl ether (300 mL) for 30 min, allowed to settle for another 30 min, and filtered. The resulting pale-yellow solid (22 g, 92%) was used directly in the next step without further purification. *R*_f_ = 0.15 (40% EA/hexane); ^1^H NMR (500.13 MHz, DMSO-*d*_6_) δ 8.68 (s, 1H), 7.57 (t, *J* = 5.8 Hz, 1H), 7.38–7.44 (m, 5H), 5.89 (ddt, *J* = 17.3, 10.3, 5.2 Hz, 1H), 5.44 (s, 2H), 5.04–5.14 (m, 2H), 4.02 (t, *J* = 5.7 Hz, 2H), 3.15 (s, 3H), 3.03 (s, 3H). ^13^C­{^1^H} NMR (125.77 MHz, DMSO-*d*_6_) δ 170.9, 167.1, 164.2, 160.4, 137.1, 136.1, 128.9, 128.4, 128.2, 115.9, 115.6, 67.8, 67.1, 43.2, 41.3, 35.3; HRMS (ESI) *m*/*z*: [M + H]^+^ Calcd for C_18_H_20_N_6_OH 337.1777; Found 337.1776.

### 1-Allyl-4,7–6-diamino-5-(benzyloxy)­pyrimido­[4,5-*d*]­pyrimidin-2­(1*H*)-one (**8**)

Compound **7** (5 g, 14.86 mmol) was dissolved in dry DCM (150 mL), cooled to 0 °C, and 2,2,2-trichloroacetyl isocyanate (16.17 mL, 133.77 mmol) was added dropwise. The reaction mixture was stirred at 0 °C in a salt-ice–water bath for 30 min, then allowed to warm to rt overnight under a nitrogen atmosphere. After confirming the complete consumption of the starting material (via TLC), the reaction mixture was cooled to 0 °C, and a methanolic NH_3_ solution (7 M, 150 mL) was added. The reaction was stirred at rt under nitrogen for 24 h, during which the transparent yellow solution gradually turned into a white suspension. The solvents were evaporated, and the resulting solid was stirred in cold diethyl ether (150 mL) for 10 min, allowed to settle for 30 min, and filtered. The crude off-white solid (5.5 g, 98%) was obtained and used directly in the next step without further purification. *R*_f_ = 0.2 (100% EA); HRMS (ESI) *m*/*z*: [M + H]^+^ Calcd for C_16_H_16_N_6_O_2_H 325.1413; Found 325.1416. Unable to obtain NMR data due to poor solubility in organic solvents.

### Di-*tert*-butyl (8-Allyl-4-(benzyloxy)-7-oxo-7,8-dihydropyrimido­[4,5-*d*]­pyrimidine-2,5-diyl)­bis­((*tert*-butoxycarbonyl)­carbamate) (**9**)

Compound **8** (5.5 g crude, 16.96 mmol, assuming 100% purity) was dissolved in dry THF (80 mL). DIPEA (11.93 mL, 67.83 mmol), DMAP (415.14 mg, 3.39 mmol), and Boc_2_O (18.69 g, 84.79 mmol) were sequentially added. The reaction mixture was stirred under a nitrogen atmosphere for 24 h. After completion, the THF layer was extracted with saturated NaHCO_3_ (50 mL), H_2_O (50 mL), and brine (50 mL). The organic phase was dried over Na_2_SO_4_, evaporated, and purified by flash chromatography (5–30% EA/hexane) on silica gel. The desired compound (2.7 g, 25% over two steps) was obtained as a sticky yellow syrup. *R*_f_ = 0.75 (40% EA/hexane); ^1^H NMR (500.13 MHz, DMSO-*d*_6_) δ 7.37–7.43 (m, 5H), 5.94 (ddt, *J* = 16.2, 10.3, 5.0 Hz, 1H), 5.57 (s, 2H), 5.15 (d, *J* = 10.4 Hz, 1H), 4.94 (s, 1H), 4.78 (d, *J* = 5.2 Hz, 2H), 1.37 (d, *J* = 107.4 Hz, 35H). ^13^C­{^1^H} NMR (125.77 MHz, DMSO-*d*_6_) δ 166.1, 160.6, 159.8, 158.3, 153.8, 149.0, 148.5, 134.5, 131.5, 128.6, 128.4, 128.2, 116.8, 93.5, 84.5, 83.7, 70.2, 44.7, 27.2, 27.2; HRMS (ESI) *m*/*z*: [M + H]^+^ Calcd for C_36_H_48_N_6_O_10_H 725.3510; Found 725.3505.

### 2-(5-(Benzyloxy)-4,7-bis­(bis­(*tert*-butoxycarbonyl)­amino)-2-oxoprimido­[4,5-*d*]­pyrimidin-1­(2*H*)-yl)­acetic Acid (**1/E**)

Compound **9** (1.88 g, 2.59 mmol) was dissolved in a dry solvent mixture of ACN (43 mL)/CCl_4_ (43 mL)/H_2_O (65 mL) and cooled to 0 °C in a salt-ice–water bath. After 10 min, NaIO_4_ (4.48 g, 20.75 mmol) was added, followed by RuCl_3_·H_2_O (116.95 mg, 518.76 μmol). The reaction mixture was stirred at 0 °C under a nitrogen atmosphere for 2 h. Upon consumption of the starting material, the organic solvents were evaporated at 35 °C, and the resulting solution was treated with H_2_O (50 mL) and extracted with EA (2 × 100 mL). The organic layer was dried over Na_2_SO_4_, concentrated, and purified via flash chromatography (20–100% EA/hexane, 1–20% MeOH/EA) on silica gel. The desired compound (1.33 g, 69%) was obtained as an off-white crunchy solid. *R*_f_ = 0.65 (10% MeOH/EA); ^1^H NMR (500.13 MHz, DMSO-*d*_6_) δ 7.37–7.42 (m, 5H), 5.58 (s, 2H), 4.85 (s, 2H), 1.47 (s, 18H), 1.27 (s, 18H). ^13^C­{^1^H} NMR (125.77 MHz, DMSO-*d*_6_) δ 168.6, 166.5, 161.1, 160.7, 158.7, 154.5, 149.4, 149.0, 134.9, 129.1, 128.9, 128.7, 93.9, 85.1, 84.2, 77.5, 70.8, 44.5, 28.7, 27.7, 27.6; HRMS (ESI) *m*/*z*: [M + H]^+^ Calcd for C_35_H_46_N_6_O_12_H 743.3252; Found 743.3251.

### 3-Cyano-2,6-difluorobenzoic Acid (**10**)

2,4-Difluorobenzonitrile (25 g, 1 equiv) was dissolved in dry THF (450 mL) and cooled to −78 °C in a dry ice-acetone bath while stirring. LDA (2.0 M, 98.85 mL, 1.1 equiv) was added dropwise over 30 min, and the reaction mixture was stirred at −78 °C for 2 h, after which excess crushed dry CO_2_ was carefully added. Stirring was continued at the same temperature for 2 h, and reaction progress was monitored by TLC (neutralizing a small aliquot with 10% HCl). Upon completion, the reaction mixture was carefully poured into chilled 10% HCl (750 mL) and stirred/shaken for 5 min (pH = 3–4). The product was extracted into EA, dried over Na_2_SO_4_, and concentrated. The resulting crude product was suspended in 10% EA/hexane solution (2×) and stirred for 30 min at rt. The precipitate was collected by filtration and dried under vacuum, yielding pure product as a light yellow solid (28 g, 85%). ^1^H NMR (500.13 MHz, DMSO-*d*_6_): δ 14.45 (s, 1H), 8.17 (ddd, *J* = 9.0, 7.5, 5.7 Hz, 1H), 7.46 (t, *J* = 8.9 Hz, 1H). ^13^C­{^1^H} NMR (125.77 MHz, DMSO-*d*_6_, ^19^F coupled): δ 163.3, 163.2, 161.8, 161.7, 161.2, 161.2, 160.6, 159.7, 159.6, 137.1, 137.0, 114.3, 114.3, 114.1, 113.0, 98.0, 98.0, 97.9, 97.9. ^19^F NMR (470.59 MHz, DMSO-*d*_6_, H decoupled): δ −100.8 (q, *J* = 7.58 Hz, 1F), −105.8 (t, *J* = 7.58 Hz, 1F). This compound is commercially available, *albeit* costly.

### 3-Cyano-2,6-difluoro-*N*-hydroxybenzamide (**11**)

To a solution of **10** (26 g, 142.07 mmol, 1 equiv) in anhydrous DCM (284 mL) with a catalytic amount of DMF (0.55 mL, 7.10 mmol, 0.05 equiv), oxalyl chloride (16 mL, 186.11 mmol, 1.31 equiv) was added dropwise over 15 min at rt. The reaction mixture was stirred for 2 h, during which the appearance of a clear solution indicated the formation of the acid chloride. After completion, DCM was evaporated at 35 °C, and the resulting residue was dissolved in 1,4-dioxane (125 mL) under a nitrogen atmosphere. In a separate dried round-bottom flask, NH_4_OH (852.40 mmol, 6 equiv, 28%) was taken under a nitrogen atmosphere and cooled to 0 °C. The acid chloride solution was slowly added dropwise to the NH_4_OH solution while maintaining the temperature at 0 °C. As the reaction progressed, a light brown precipitate formed, indicating the formation of the amide. After 4 h (TLC-monitored completion), the precipitate was collected by vacuum filtration and washed with water. The filtrate was extracted with EA, concentrated, and combined with the solid to optimize the yield. The crude product was then washed with 10% EA/hexane, yielding the pure compound as a light yellow solid (18 g, 70%). ^1^H NMR (500.13 MHz, DMSO-*d*_6_): δ 8.28 (s, 1H), 8.16–8.04 (m, 2H), 7.45 (t, *J* = 8.7 Hz, 1H). ^13^C­{^1^H} NMR (125.77 MHz, DMSO-*d*_6_, ^19^F coupled): δ 162.6, 162.6, 162.5, 160.9, 160.8, 160.6, 160.5, 159.5, 158.8, 158.8, 135.7, 135.6, 117.1, 116.9, 116.9, 116.7, 114.0, 113.9, 113.8, 113.7, 113.1, 97.3, 97.3, 97.2, 97.2. ^19^F NMR (470.59 MHz, DMSO-*d*_6_, H decoupled): δ −102.8 (td, *J* = 13.18, 6.02 Hz, 1F), −107.9 (t, *J* = 8.28 Hz, 1F). HRMS (ESI) *m*/*z*: [M + H]^+^ Calcd for C_8_H_4_F_2_N_2_OH 183.0370; Found 183.0367.

### 2,4-Difluoroisophthalonitrile (**12**)

Amide **11** (16.50 g, 90.64 mmol, 1 equiv) and POCl_3_ (12.70 mL, 135.97 mmol, 1.5 equiv) were heated in acetonitrile at 85 °C in an oil bath for 5 h. After completion, the reaction mixture was cooled, and the acetonitrile was removed under vacuum. The crude residue was carefully treated with saturated NaHCO_3_, then extracted with EA (2×). The organic layer was dried, concentrated, and the crude product was recrystallized from a small amount of DCM, yielding the desired white crystalline solid (14 g, 94%). ^1^H NMR (500.13 MHz, DMSO-*d*_6_): δ 8.81–8.18 (m, 1H), 7.93–7.45 (m, 1H). ^13^C­{^1^H} NMR (125.74 MHz, DMSO-*d*_6_, ^19^F coupled): δ 166.0, 166.0, 165.0, 165.0, 163.9, 163.9, 162.9, 162.9, 162.9, 140.7, 140.6, 140.6, 140.5, 114.5, 114.4, 114.3, 114.2, 112.0, 112.0, 108.3, 98.6, 98.6, 98.5, 98.5, 93.6, 93.4, 93.4, 93.3. ^19^F NMR (470.59 MHz, DMSO-*d*_6_, H decoupled): δ −95.2 (ddd, *J* = 8.7, 6.00, 4.10 Hz, 1F), −98.8 (m, 1F).This compound is commercially available, *albeit* costly.

### Pyrimido­[4,5-*f*]­quinazoline-2,4,8,10-tetraamine (**13**)

A mixture of compound **12** (5 g, 30.48 mmol, 1 equiv) and guanidine carbonate (243.87 mmol, 8 equiv) was heated in N,N-dimethylacetamide (152 mL) at 145 °C in a silicone oil bath for 24 h in a pressure rated round-bottom flask. After completion, the reaction mixture was evaporated using a rotary evaporator, yielding a slurry. To this slurry, DI water (100 mL) was added, leading to precipitate formation. The solid was collected by vacuum filtration and then washed with EA and acetone to obtain the pure tricyclic compound as a yellow solid (3.7 g, 50%). NMR was obtained from the crude reaction mixture after EA washing. ^1^H NMR (500.13 MHz, DMSO-*d*_6_): δ 10.37 (d, *J* = 4.9 Hz, 1H), 7.92 (d, *J* = 9.1 Hz, 1H), 7.31 (d, *J* = 4.8 Hz, 1H), 7.10 (bs, 2H), 6.72 (d, *J* = 9.0 Hz, 1H), 6.34 (bs, 2H), 6.04 (bs, 2H). ^13^C­{^1^H} NMR (125.74 MHz, DMSO-*d*_6_): 163.9, 162.1, 161.9, 160.5, 158.0, 152.7, 127.1, 117.7, 101.7, 101.2. HRMS (ESI) *m*/*z*: [M + H]^+^ Calcd for C_10_H_10_N_8_H 243.1107; Found 243.1101.

### 6-Bromopyrimido­[4,5-*f*]­quinazoline-2,4,8,10-tetraamine (**14**)

To a solution of tricyclic **13** (10.50 g, 43.37 mmol, 1 equiv) in anhydrous DMF (193 mL), NBS (49.88 mmol, 1.15 equiv) was added portion-wise over 15 min. The reaction mixture was stirred overnight at rt. After completion, the mixture was concentrated to dryness, and the crude residue was treated with EA (500 mL) and stirred for 30 min. The resulting precipitate was collected via vacuum filtration, washed several times with EA, and dried under high vacuum, yielding the crude brominated compound as a greenish-yellow solid (13.0 g, 93.39% crude). NMR was obtained from the crude reaction mixture after EA washing. ^1^H NMR (300.13 MHz, DMSO-*d*_6_): δ 10.97 (bs, 1H), 8.52 (s, 1H), 7.95 (bs, 1H), 7.40 (bs, 2H), 6.68 (bs, 4H). ^13^C­{^1^H} NMR (125.77 MHz, DMSO-*d*_6_): δ 163.7, 161.6, 161.2, 151.2, 140.6, 133.1, 117.9, 105.4, 103.8, 101.5. HRMS (ESI) *m*/*z*: [M + H]^+^ Calcd for C_10_H_9_BrN_8_H 321.0212; Found 321.0208.

### Tetra-*tert*-butyl (6-Bromopyrimido­[4,5-*f*]­quinazoline-2,4,8,10-tetrayl)­tetrakis­((*tert*-butoxycarbonyl)­carbamate) (**15**)

To a solution of **14** (13 g, 40.62 mmol, 1 equiv) in anhydrous THF, DIPEA (64 mL, 365.61 mmol, 9 equiv) and DMAP (0.99 g, 81.25 mmol, 0.2 equiv) were added, followed by Boc_2_O (88 g, 406.23 mmol, 10 equiv) at rt. The reaction mixture was stirred for 15 h. After this initial period, an additional 5 equiv of Boc_2_O was added, and the mixture was heated to 45 °C in an oil bath, stirring additional 10 h. Another 5 equiv of Boc_2_O was added, and the reaction was continued at 45 °C for another 10 h. Upon completion, the reaction mixture was extracted with EA and saturated NaHCO_3_. The organic layer was dried over Na_2_SO_4_, concentrated, and purified by silica gel column chromatography (20% EA/Hex), yielding pure compound **15** as a light yellow solid (17 g, 35% over two steps). ^1^H NMR (500.13 MHz, DMSO-*d*_6_): δ 9.03 (s, 1H), 1.43 (s, 36H), 1.31 (s, 18H), 1.24 (s, 18H). ^13^C­{^1^H} NMR (125.74 MHz, DMSO-*d*_6_): δ 170.8, 160.3, 150.5, 150.2, 149.7, 149.3, 148.8, 133.5, 124.1, 119.2, 116.1, 84.8, 84.6, 84.5, 84.0, 83.8, 27.8, 27.7, 27.6, 27.6, 27.3. HRMS (ESI) *m*/*z*: [M + Na]^+^ Calcd for C_50_H_73_BrN_8_O_16_Na 1145.4200; Found 1145.4179.

### Ethyl 2-(2,4,8,10-Tetrakis­(bis­(*tert*-butoxycarbonyl)­amino)­Pyrimido­[4,5-*f*]­quinazolin-6-yl)­acetate (**16**)

In an oven-dried round-bottom flask (RBF), ZnBr_2_ (562 mg, 2.50 mmol, 1.4 equiv, 99.999% Zn Assay) was heated under high vacuum at 145 °C in an oil bath for 1 h in a silicone oil bath. The flask was then removed from the oil bath and cooled to rt. Meanwhile, the oil bath temperature was adjusted to 80 °C. To the cooled flask, a mixture of bromo compound (2.00 g, 1.78 mmol, 1 equiv), ethyl (tributylstannyl)­acetate (5.00 mmol, 2.8 equiv), and dichlorobis­(tri-*o*-tolylphosphine)­palladium­(II) (0.14 mmol, 0.08 equiv) in anhydrous DMF (40 mL) was added under an inert atmosphere at rt. The flask was then transferred to the preheated 80 °C oil-bath, and the mixture was gradually warmed to 118 °C and stirred for 4 h. The progress of the reaction was monitored by TLC, and after complete consumption of the starting material, the mixture was concentrated at 40 °C. The resulting crude residue was dissolved in EA, washed with saturated NaHCO_3_, and the organic layer was separated, dried over Na_2_SO_4_, and concentrated. Purification via silica gel column chromatography (20% EA/Hex) yielded the ester as a light yellow solid (0.8 g, 15%), accompanied by a mixture of 6/7/8 Boc compounds coeluting at the same *R*_f_ value. HRMS (ESI) *m*/*z*: [M + H]^+^ Calcd for C_44_H_64_N_8_O_14_H 929.4620; Found 929.4601. Due to the product mixture, ^1^H and ^13^C NMR spectra showed indiscernible signals and therefore were not included.

### 2-(10-Amino-2,4,8-tris­(bis­(*tert*-butoxycarbonyl)­amino)­pyrimido­[4,5-*f*]­quinazolin-6-yl)­acetic Acid (**2/K**)

Ester **16** (500 mg, 0.44 mmol, 1 equiv) was dissolved in a mixture of EtOH/THF (7 mL, 1:1 vol). To this solution, 2 M NaOH (1.35 mL, 2.22 mmol, 5 equiv) was slowly added at rt. The progress of the reaction was monitored by TLC, and upon complete consumption of the ester, the reaction mixture was transferred to a separating funnel containing 10% HCl and EA. The aqueous layer was extracted with EA (3×), and the combined organic phase was dried over Na_2_SO_4_, concentrated, and purified via column chromatography (50% EA/Hex, 100% EA and finally 20% MeOH/EA) to obtain the final acid compound as a white amorphous solid with 6–7 Boc groups Yield: 0.2 g, 68%. *Note: Prolonged reaction times led to partial Boc deprotection and minor decarboxylation.* ^1^H NMR (300.13 MHz, DMSO-*d*_6_, two Boc compounds (1:1.32)): δ 10.47 (s, 1H), 8.64 (s, 1H), 8.49 (s, 1H), 8.15 (s, 2H), 7.99 (s, 3H), 4.09 (s, 2H), 4.04 (s, 2H), 1.50 (s, 18H), 1.40 (s, 58H), 1.26 (s, 40H). HRMS (ESI) *m*/*z*: [M + H]^+^ Calcd for C_37_H_52_N_8_O_12_H 801.3783; Found 801.3780. Due to the product mixture, ^13^C NMR spectrum showed indiscernible signals and therefore was not included.

### Allyl (*R*)-11-((((9*H*-Fluoren-9-yl)­methoxy)­carbonyl)­amino)-13-(2-(5-(benzyloxy)-4,7-bis­(bis­(*tert*-kutoxycarbonyl)­amino)-2-oxopyrimido­[4,5-*d*]­pyrimidin-1­(2*H*)-yl)­acetyl)-2,2-dimethyl-3,6,9-trioxa-13-azapentadecan-15-oate (**18a**)

A mixture of **1**/**E** (1.00 mmol, 1 equiv) and DIPEA (1.00 mmol, 1 equiv) in anhydrous DMF (0.2 M) was prepared. HBTU (1.00 mmol, 1 equiv) was then added in one portion at rt. After 10 min of stirring, backbone **17** (1.00 mmol, 1 equiv), dissolved in DMF (0.2 M), was added to the reaction mixture. The reaction was maintained at 45 °C in an oil bath. The reaction progress was monitored by TLC (EA/hexane (50:50)), and upon complete consumption of the starting material, DMF was removed under vacuum at 45 °C. The crude material was then treated with 1% HCl and EA. The organic layers (2×) were separated from the aqueous layer using a separating funnel. The combined organic layers were dried, concentrated, and purified via flash silica gel column chromatography (80% EA/Hex), yielding the final product as a white amorphous solid with a 70% (0.90 g) yield. ^1^H NMR (500.13 MHz, DMSO-*d*_6_, rotamers): δ 7.89–8.26 (m, 3H), 7.69–7.71 (m, 2H), 7.21–7.43 (m, 9H), 5.83–6.00 (m, 1H), 5.56–5.73 (s/s, 2H), 5.00–5.39 (m, 4H), 3.40–3.67 (m, 7H), 3.39–3.90 (m, 10H), 3.11–3.28 (m, 2H), 1.27–1.44 (s/s, 35H), 1.05–1.09 (s/s 8H). ^13^C­{^1^H} NMR (125.77 MHz, DMSO-*d*_6_, rotamers): δ 166.5, 156.3, 149.3, 144.3, 141.2, 132.7, 132.6, 129.1, 129.0, 128.9, 128.6, 128.1, 127.5, 125.7, 120.6, 118.2, 93.9, 84.9, 84.8, 84.2, 72.7, 70.9, 70.8, 70.7, 70.3, 70.2, 70.1, 66.0, 65.9, 65.3, 61.1, 61.0, 50.5, 48.6, 47.2, 27.8, 27.8, 27.7, 27.7, 27.6, 27.6, 27.6, 27.3; HRMS (ESI) *m*/*z*: [M + H]^+^ Calcd for C_66_H_86_N_8_O_18_H 1279.6138; Found 1279.6132.

### Allyl (*R*)-11-((((9*H*-Fluoren-9-yl)­methoxy)­carbonyl)­amino)-13-(2-(10-amino-4-(bis­(*tert*-butoxycarbonyl)­amino)-2,8-bis­((*tert*-butoxycarbonyl)­amino)­pyrimido­[4,5-*f*]­quinazolin-6-yl)­acetyl)-2,2-dimethyl-3,6,9-trioxa-13-azapentadecan-15-oate (**18b**)

This compound was synthesized following the same procedure as described for **18a**, using compound **2/K** and backbone **17** as the starting materials. The product was obtained as a white amorphous solid after chromatography (80% EA/Hex) with a 60% (0.8 g) yield. ^1^H NMR (500.13 MHz, DMSO-*d*_6_, rotamers, Tetra Boc): δ 10.45–11.60 (s/s, 1H), 10.21 (s, 1H), 6.96–8.57 (m, 13H), 5.81–5.94 (m, 1H), 5.13–5.35 (m, 2H), 4.48–4.77 (m, 3H), 4.12–4.46 (m, 4H), 3.37– 4.00 (m, 10H), 2.98 – 3.29 (m, 3H), 1.24–1.51 (s/s, 35H), 1.03–1.11 (m, 8H). ^13^C­{^1^H} NMR (125.77 MHz, DMSO-*d*_6_, rotamers): δ 171.4, 170.8, 170.2, 169.7, 160.8, 156.2, 156.1, 156.0, 151.4, 151.2, 149.3, 144.3, 141.2, 132.7, 132.7, 132.6, 132.5, 132.4, 128.1, 127.5, 126.4, 126.3, 124.9, 120.6, 118.7, 118.2, 118.2, 83.7, 82.8, 82.5, 82.5, 82.4, 82.4, 80.4, 72.7, 70.9, 70.5, 70.3, 70.1, 65.8, 65.2, 65.2, 61.1, 60.2, 47.2, 28.4, 28.3, 28.3, 28.1, 28.0, 28.0, 27.9, 27.8, 27.8, 27.7, 27.7, 27.6; HRMS (ESI) *m*/*z*: [M + H]^+^ Calcd for C_68_H_92_N_10_O_18_H 1337.6669; Found 1337.6666.

### (*R*)-11-((((9*H*-Fluoren-9-yl)­methoxy)­carbonyl)­amino)-13-(2-(5-(benzyloxy)-4,7-bis­(bis­(*tert*-butoxycarbonyl)­amino)-2-oxopyrimido­[4,5-*d*]­pyrimidin-1­(2*H*)-yl)­acetyl)-2,2-dimethyl-3,6,9-trioxa-13-azapentadecan-15-oic Acid (**19**)

Palladium tetrakis­(triphenylphosphine) (0.05 mmol, 0.05 equiv) was added to a mixture of phenyl silane (2.00 mmol, 1 equiv) and allyl monomer **18a** (1.00 mmol, 1 equiv) in anhydrous DCM (0.1 M) at rt. The reaction progress was monitored by TLC, and complete conversion of the starting material to the corresponding acid was observed over a period of 15 h. After completion, silica gel was added to the reaction mixture at rt, and the solvents were removed using a rotary evaporator. The crude silica gel-absorbed product was then purified by flash silica gel chromatography (80% EA/Hex then 20% MeOH/EA), yielding the desired acid product as a yellowish amorphous solid. Yield: 0.9 g, 72%. ^1^H NMR (500.13 MHz, DMSO-*d*_6_, rotamers): δ 12.82 (bs, 1H), 7.88/7.89 (s/s, 2H), 7.67–7.72 (m, 2H), 7.61–7.73 (m, 2H), 7.56–7.58 (m, 2H), 7.38–7.44 (m, 6H), 7.31–7.34 (m, 2H), 5.55–5.58 (s/s, 2H), 5.00–5.23 (m, 2H), 3.90–4.46 (m, 6H), 3.37–3.78 (m, 10H), 3.11–3.29 (m, 2H), 1.18–1.6 (m, 35H), 1.04/1.09 (s/s, 9H). ^13^C­{^1^H} NMR (125.77 MHz, DMSO-*d*_6_, rotamers): δ 161.5, 154.5, 149.3, 149.3, 149.0, 144.3, 141.2, 141.2, 132.5, 132.5, 132.0, 131.9, 129.3, 129.2, 129.1, 128.9, 128.6, 128.6, 128.1, 127.5, 125.7, 120.6, 93.9, 84.9, 84.8, 84.2, 72.7, 70.9, 70.8, 70.7, 70.3, 70.2, 70.1, 66.0, 65.9, 61.1, 61.0, 47.2, 27.8, 27.7, 27.7, 27.6, 27.6, 27.6; HRMS (ESI) *m*/*z*: [M + H]^+^ Calcd for C_63_H_82_N_8_O_18_H 1239.5820; Found 1239.5822.

### (*R*)-11-((((9*H*-Fluoren-9-yl)­methoxy)­carbonyl)­amino)-13-(2-(10-amino-2,4,8-tris­(bis­(*tert*-butoxycarbonyl)­amino)­pyrimido­[4,5-*f*]­quinazolin-6-yl)­acetyl)-2,2-dimethyl-3,6,9-trioxa-13-azapentadecan-15-oic Acid (**20**)

This compound was synthesized following the same procedure as described for compound **19**, using compound **18b** as the starting material. The product was obtained as a yellowish amorphous solid after chromatography (80% EA/Hex then 20% MeOH/EA) with a 65% (0.9 g) yield. ^1^H NMR (500.13 MHz, DMSO-*d*_6_, rotamers, Tetra Boc): δ 12.78 (bs, 1H), 10.18–10.65 (m, 2H), 8.45/8.52 (s/s, 1H), 7.21–8.16 (m, 13H), 3.41–4.62 (m, 17H), 2.96–3.41 (m, 5H), 1.24–1.56 (m, 36H), 1.03/1.08 (s/s, 9H). ^13^C­{^1^H} NMR (125.77 MHz, DMSO-*d*_6_, rotamers): δ 171.9, 171.8, 171.7, 171.3, 163.3, 157.1, 156.2, 156.0, 151.4, 151.0, 141.2, 141.1, 132.0, 131.9, 131.2, 129.3, 129.2, 128.1, 128.1, 127.5, 125.6, 120.6, 114.2, 108.2, 82.5, 82.4, 82.4, 82.3, 80.4, 72.7, 72.7, 70.9, 70.7, 70.5, 70.1, 66.1, 65.9, 61.1, 51.0, 49.9, 49.0, 47.2, 35.8, 35.6, 28.5, 28.4, 28.4, 28.4, 28.3, 28.1, 28.0, 28.0, 27.9, 27.7, 27.7; HRMS (ESI) *m*/*z*: [M + H]^+^ Calcd for C_65_H_88_N_10_O_18_H 1297.6356; Found 1297.6347.

## Supplementary Material



## Data Availability

The data underlying this study are available in the published article and its Supporting Information.

## Notes

Nucleobase and monomer synthesis, along with the NMR and MS spectra (PDF)
