# Supplementary material for: Synthesis of Janus Bases for Targeting C‑G and U–U Pairs of CUG-RNA Repeats Associated with Myotonic Dystrophy Type 1
Source: ACS Omega. 2025 Oct 6;10(41):49252–9. doi: 10.1021/acsomega.5c09489 (PMC12547544; doi:10.1021/acsomega.5c09489)
Supplement: Supplementary file 1 [file ao5c09489_si_001.pdf]

SUPPLEMENTAL INFORMATION

Synthesis of Janus Bases for Targeting C-G and U-U Pairs of CUG-RNA Repeats Associated with Myotonic Dystrophy Type 1

Shivaji A. Thadke,† J. Dinithi R. Perera,† Savani W. Thrikawala,† and Danith H. Ly*

Email: dly@andrew.cmu.edu

Department of Chemistry and Institute for Bimolecular Design and Discovery (IBD), Carnegie Mellon University, 4400 Fifth Avenue, Pittsburgh, Pennsylvania 15213, United States

Table of Contents

		Page
**Figure S1**	Chemical structures of Janus base F derivatives	S2
**Figure S2**	Spectroscopic data for Compound 3	S3-4
**Figure S3**	Spectroscopic data for Compound 4	S5-6
**Figure S4**	Spectroscopic data for Compound 5	S7-8
**Figure S5**	Spectroscopic data for Compound 6	S9-10
**Figure S6**	Spectroscopic data for Compound 7	S11-12
**Figure S7**	Spectroscopic data for Compound 8	S13
**Figure S8**	Spectroscopic data for Compound 9	S14-15
**Figure S9**	Spectroscopic data for Compound 1/E	S16-17
**Figure S10**	Spectroscopic data for Compound 10	S18-19
**Figure S11**	Spectroscopic data for Compound 11	S20-22
**Figure S12**	Spectroscopic data for Compound 12`	S23-24
**Figure S13**	Spectroscopic data for Compound 13	S25-26
**Figure S14**	Spectroscopic data for Compound 14	S27-28
**Figure S15**	Spectroscopic data for Compound 15	S29-30
**Figure S16**	Spectroscopic data for Compound 16	S31
**Figure S17**	Spectroscopic data for Compound 2/K	S32
**Figure S18**	Spectroscopic data for Compound 18a	S33-34
**Figure S19**	Spectroscopic data for Compound 18b	S35-36
**Figure S20**	Spectroscopic data for Compound 19	S37-38
**Figure S21**	Spectroscopic data for Compound 20	S39-40

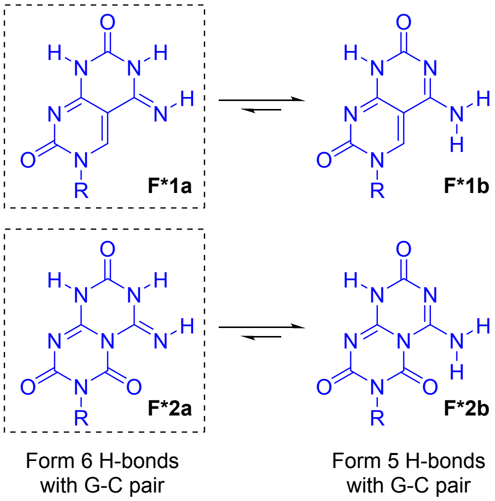

Figure S1. Chemical structures of Janus base F derivatives optimized for enhanced hydrogen bonding and/or base stacking interactions. Thermodynamic considerations suggest that **F*1a** and **F*2a** could be the predominant tautomers in the bound state (Wang *et al. Proc. Natl. Acad. Sci. U.S.A.* **2011**, *108(43)*, 17644-17648).

(a)

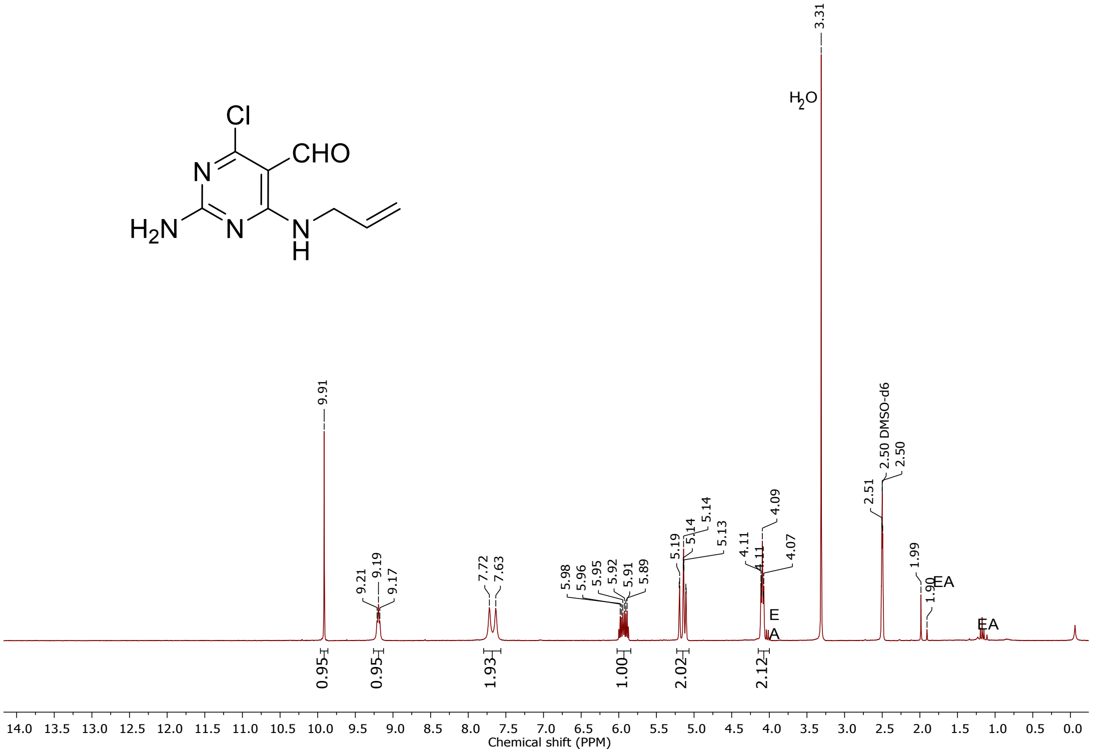

Cl
CHO
N
H2N
N
N
H
H2O
3.31
9.91
9.21
9.19
9.17
7.72
7.63
5.98
5.96
5.95
5.92
5.91
5.89
5.19
5.14
5.14
5.13
4.11
4.11
4.09
4.07
2.51
2.50 DMSO-d6
2.50
1.99
1.90
EA
0.95
0.95
1.93
1.00
2.02
2.12
14.0 13.5 13.0 12.5 12.0 11.5 11.0 10.5 10.0 9.5 9.0 8.5 8.0 7.5 7.0 6.5 6.0 5.5 5.0 4.5 4.0 3.5 3.0 2.5 2.0 1.5 1.0 0.5 0.0
Chemical shift (PPM)

(b)

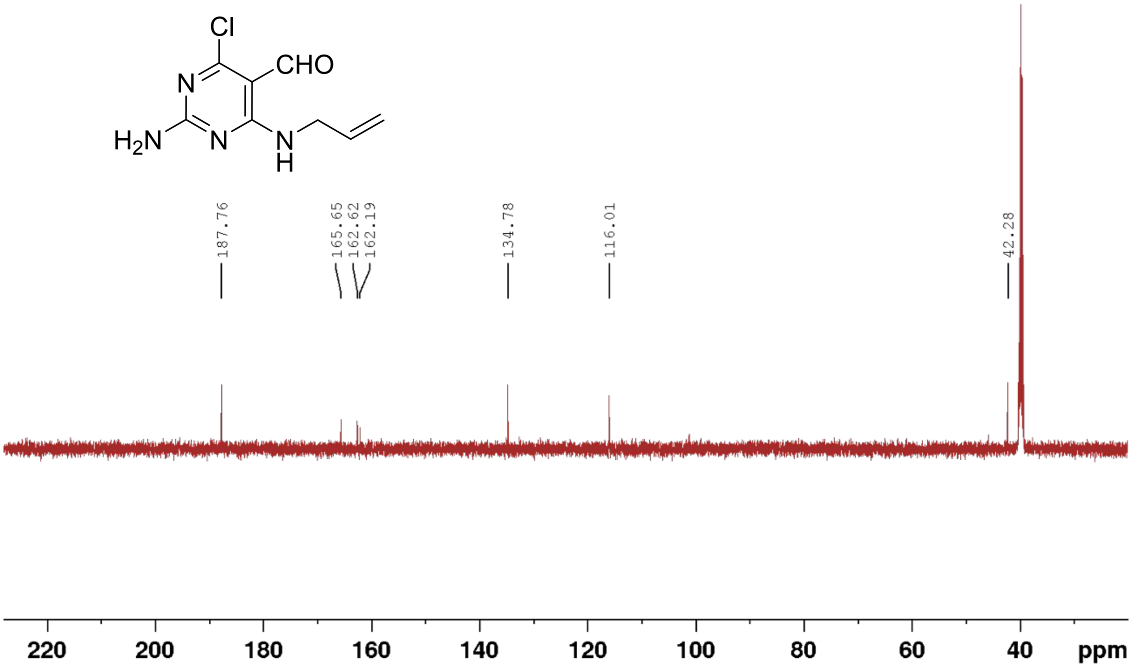

Cl
CHO
N
H2N
N
N
H
187.76
165.65
162.62
162.19
134.78
116.01
42.28
220 200 180 160 140 120 100 80 60 40 ppm

(c)

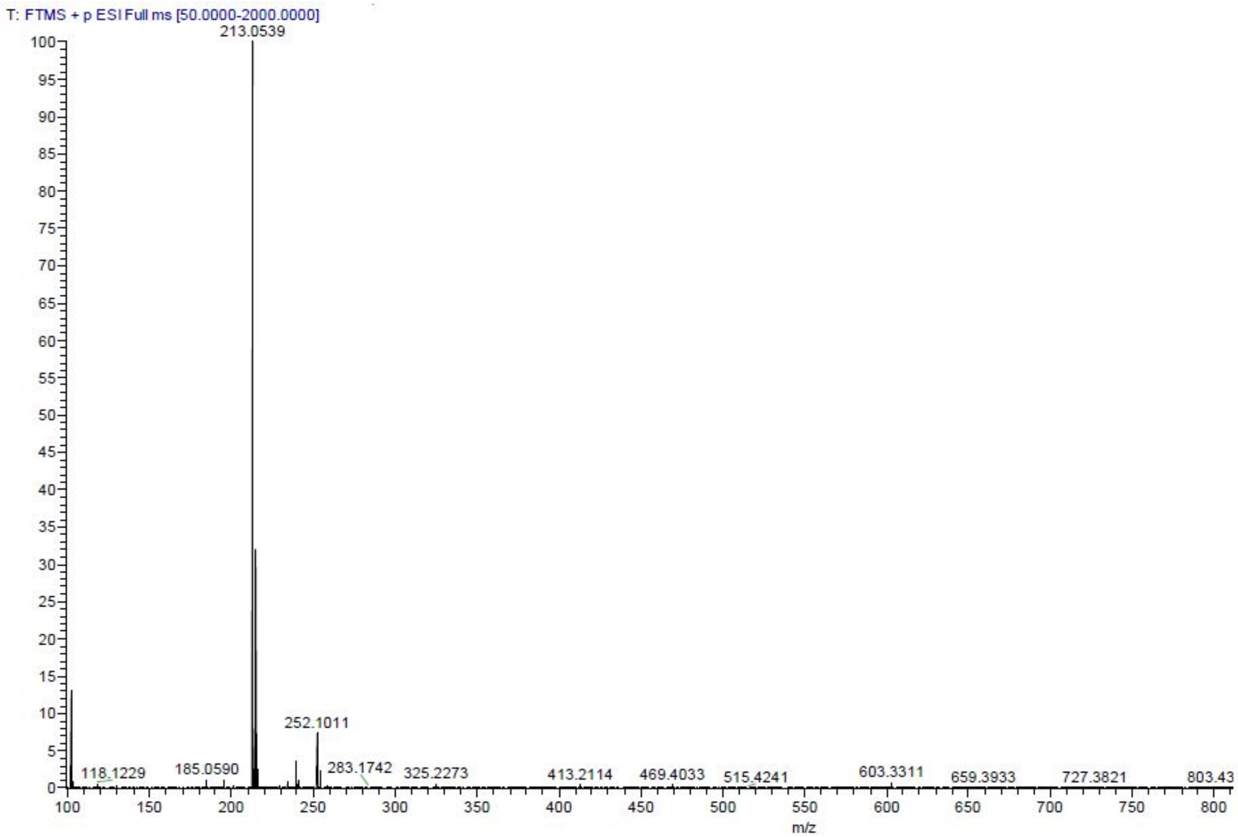

Figure S2. Spectroscopic data for compound **3**: (**a**) ^{1}H NMR (500.13 MHz, DMSO-d_6), (**b**) ^{13}C{^{1}H} NMR (125.77 MHz, DMSO-d_6), and (**c**) HRMS (ESI).

(a)

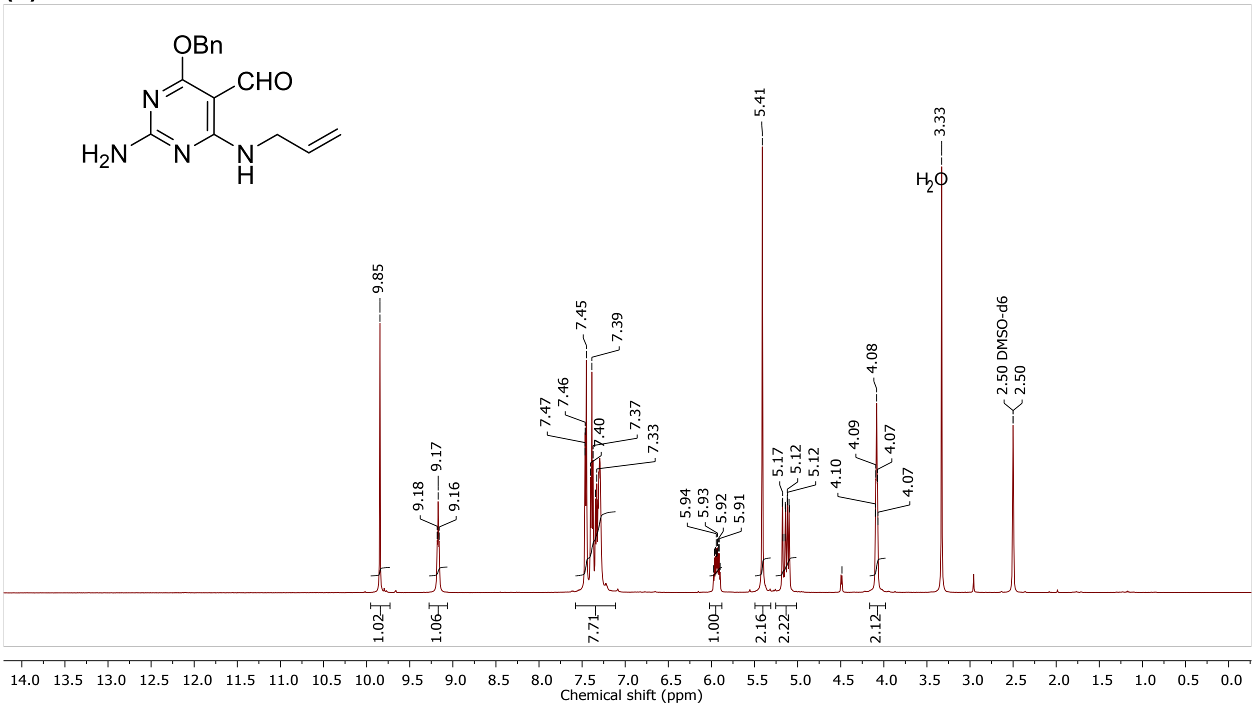

(b)

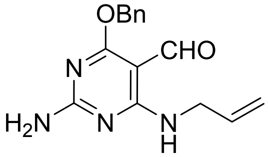
OBn
CHO
N
H2N
N
N
H

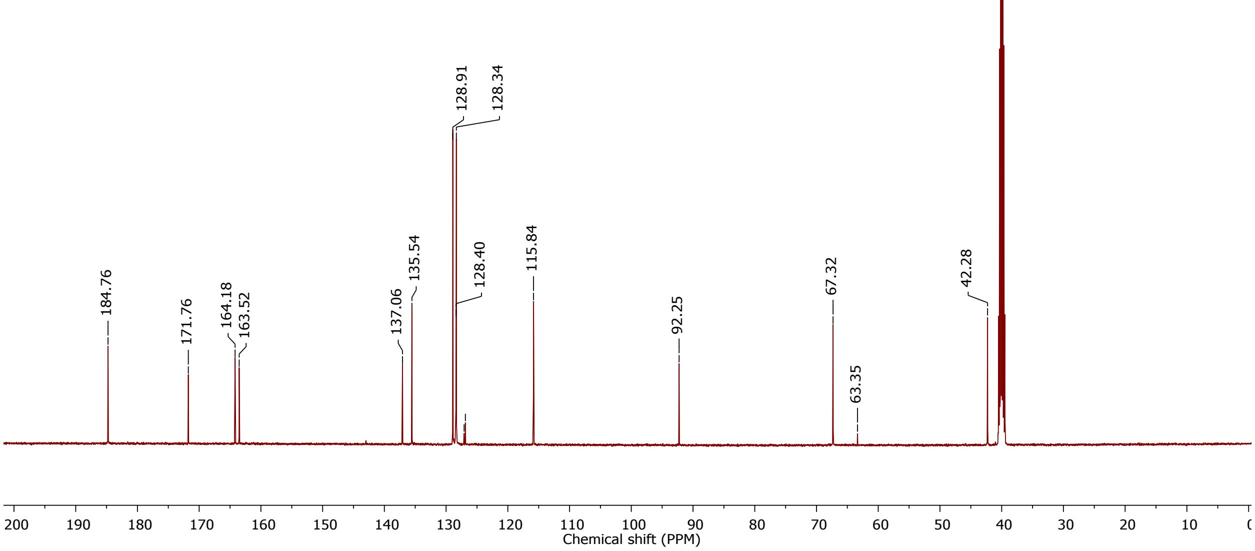
184.76
171.76
164.18
163.52
137.06
135.54
128.91
128.34
128.40
115.84
92.25
67.32
63.35
42.28
Chemical shift (PPM)

(c)

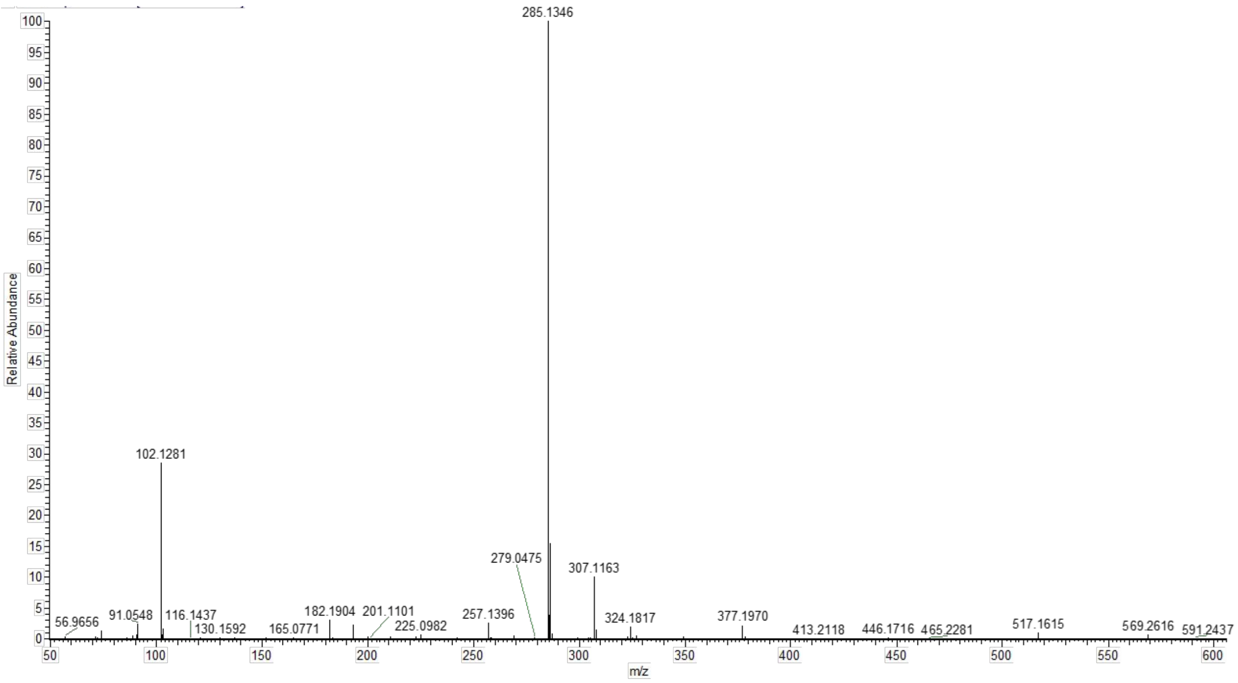

Figure S3. Spectroscopic data for compound **4**: (**a**) ^{1}H NMR (500.13 MHz, DMSO-d_6), (**b**) $^{13}C\{^{1}H\}$ NMR (125.77 MHz, DMSO-d_6), and (**c**) HRMS.

(a)

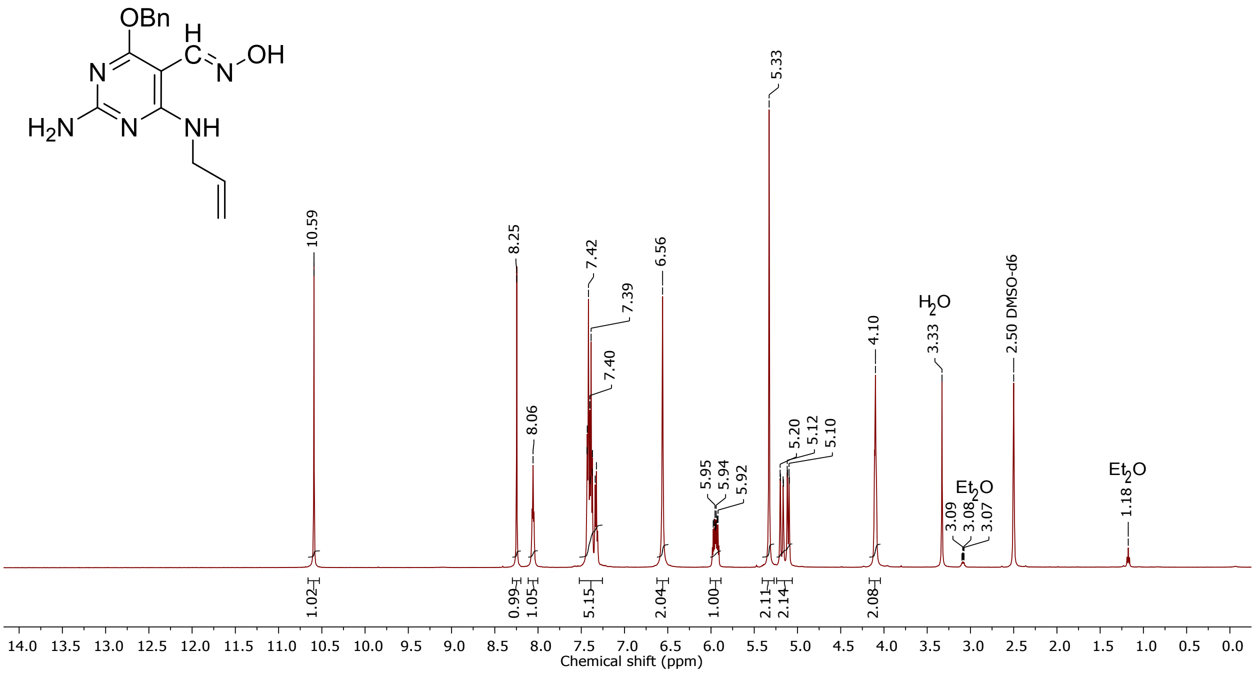
OBn
H
C
N
OH
N
H2N
N
NH
10.59
8.25
8.06
7.42
7.39
7.40
6.56
5.95
5.94
5.92
5.33
5.20
5.12
5.10
4.10
H2O
3.33
3.09
3.08
3.07
Et2O
2.50 DMSO-d6
Et2O
1.18
1.02
0.99
1.05
5.15
2.04
1.00
2.11
2.14
2.08
14.0 13.5 13.0 12.5 12.0 11.5 11.0 10.5 10.0 9.5 9.0 8.5 8.0 7.5 7.0 6.5 6.0 5.5 5.0 4.5 4.0 3.5 3.0 2.5 2.0 1.5 1.0 0.5 0.0
Chemical shift (ppm)

(b)

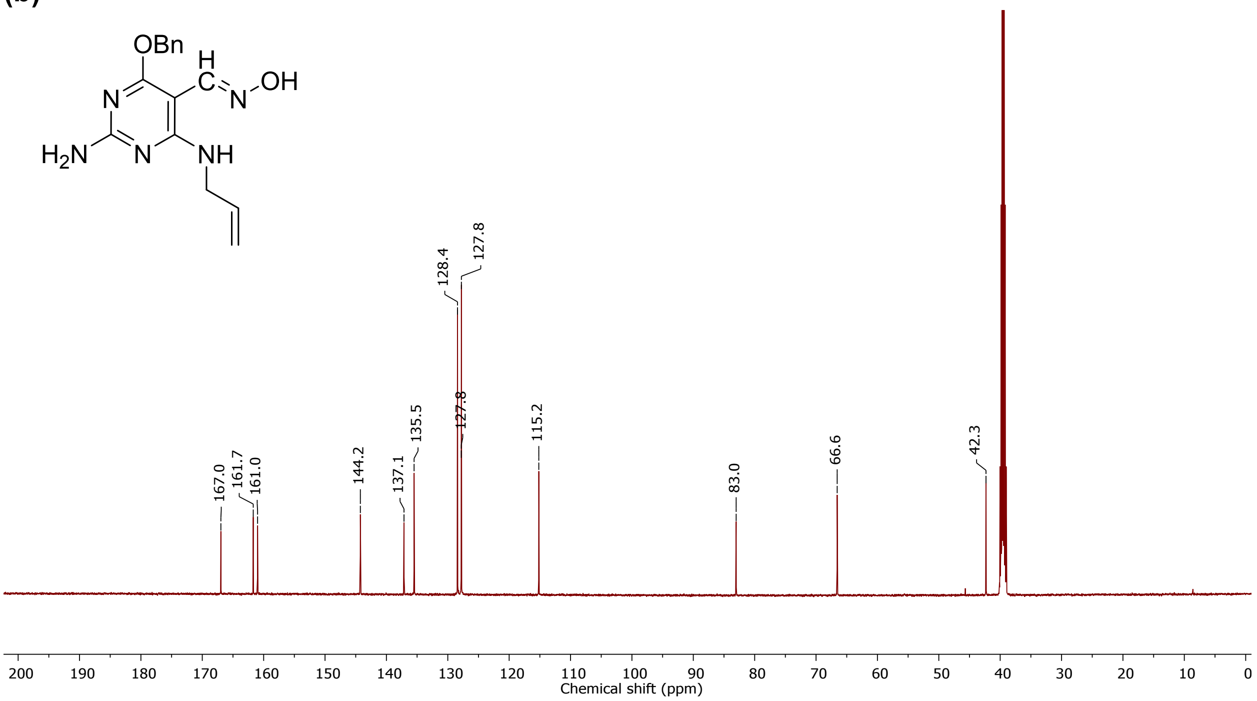
OBn
H
C
N
OH
N
H2N
N
NH
167.0
161.7
161.0
144.2
137.1
135.5
128.4
127.8
127.8
115.2
83.0
66.6
42.3
200 190 180 170 160 150 140 130 120 110 100 90 80 70 60 50 40 30 20 10 0
Chemical shift (ppm)

(c)

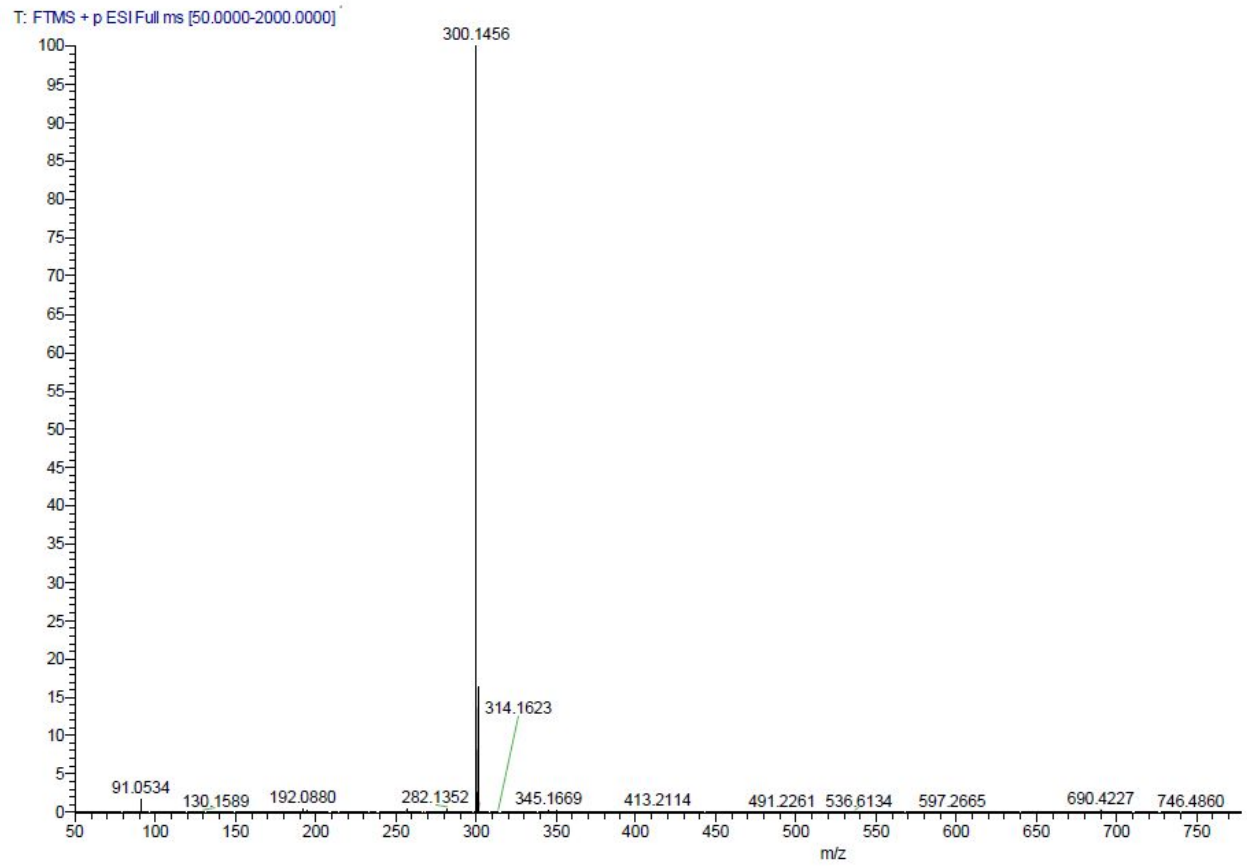

Figure S4. Spectroscopic data for compound **5**: (**a**) ^{1}H NMR (500.13 MHz, DMSO-d_6), (**b**) $^{13}C\{^{1}H\}$ NMR (125.77 MHz, DMSO-d_6), and (**c**) HRMS (ESI).

(a)

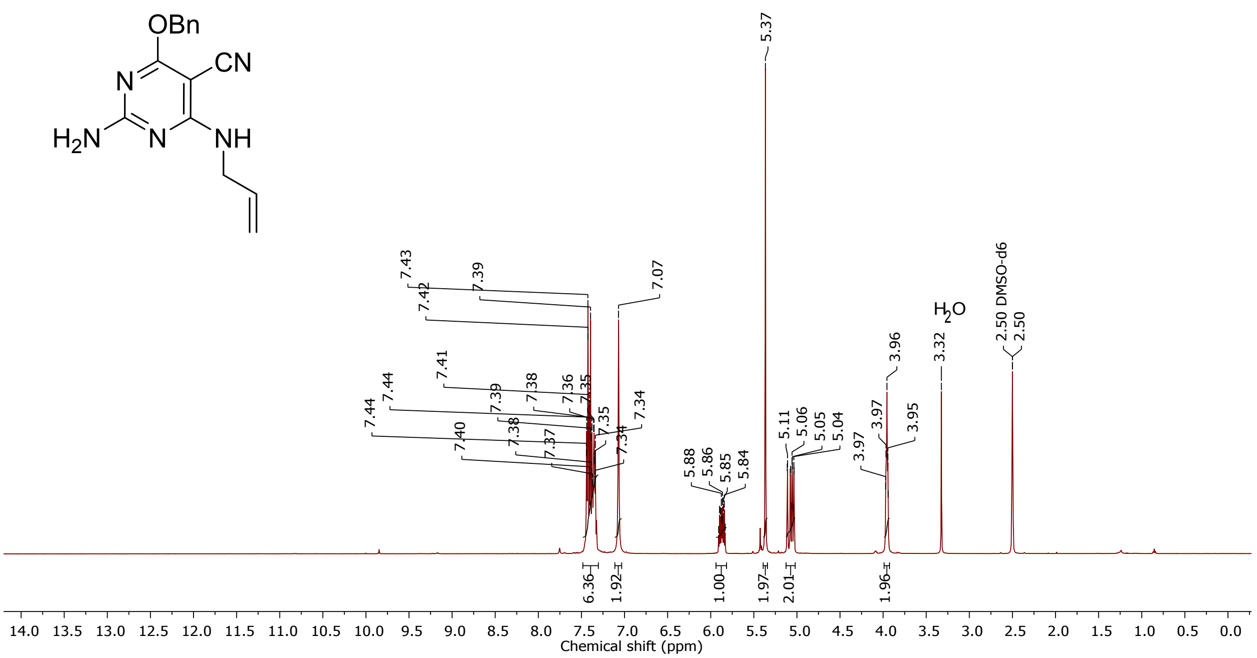
OBn
CN
N
H2N
N
NH
5.37
7.43
7.42
7.39
7.07
7.41
7.44
7.44
7.39
7.38
7.36
7.35
7.38
7.40
7.37
7.35
7.34
7.34
5.88
5.86
5.85
5.84
5.11
5.06
5.05
5.04
3.97
3.97
3.96
3.95
H2O
3.32
2.50 DMSO-d6
2.50
6.36
1.92
1.00
1.97
2.01
1.96
14.0 13.5 13.0 12.5 12.0 11.5 11.0 10.5 10.0 9.5 9.0 8.5 8.0 7.5 7.0 6.5 6.0 5.5 5.0 4.5 4.0 3.5 3.0 2.5 2.0 1.5 1.0 0.5 0.0
Chemical shift (ppm)

(b)

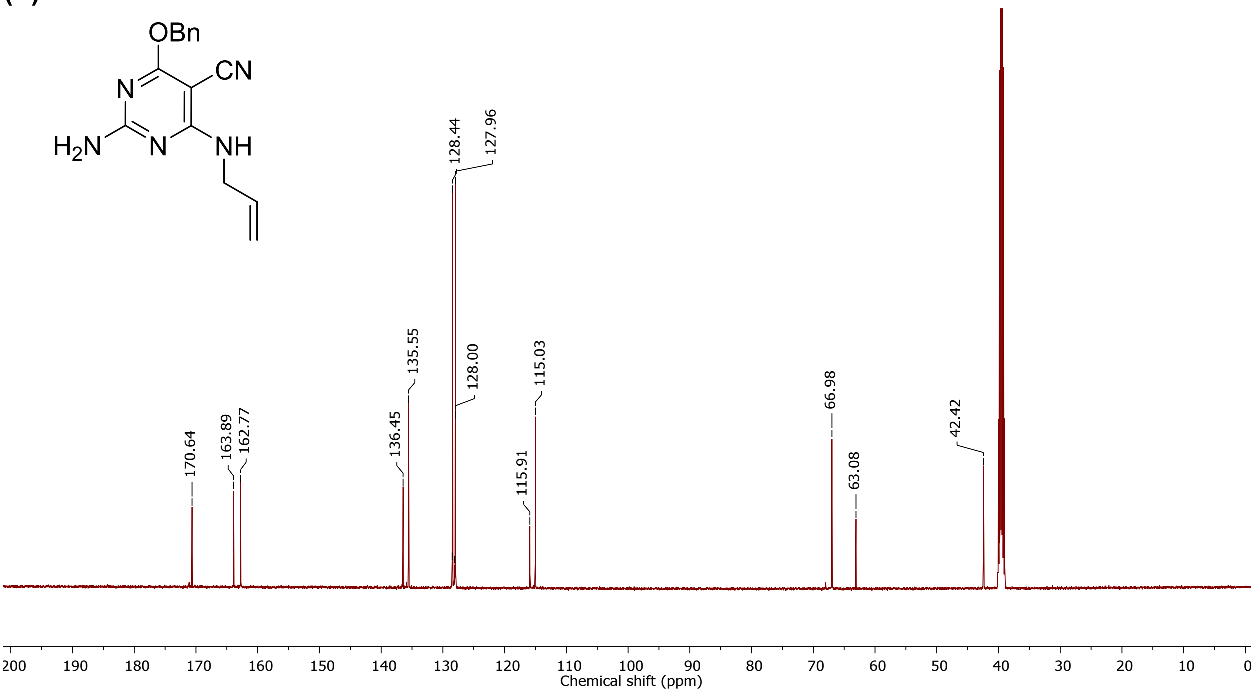
OBn
CN
N
H2N
N
NH
128.44
127.96
170.64
163.89
162.77
136.45
135.55
128.00
115.91
115.03
66.98
63.08
42.42
200 190 180 170 160 150 140 130 120 110 100 90 80 70 60 50 40 30 20 10 0
Chemical shift (ppm)

(c)

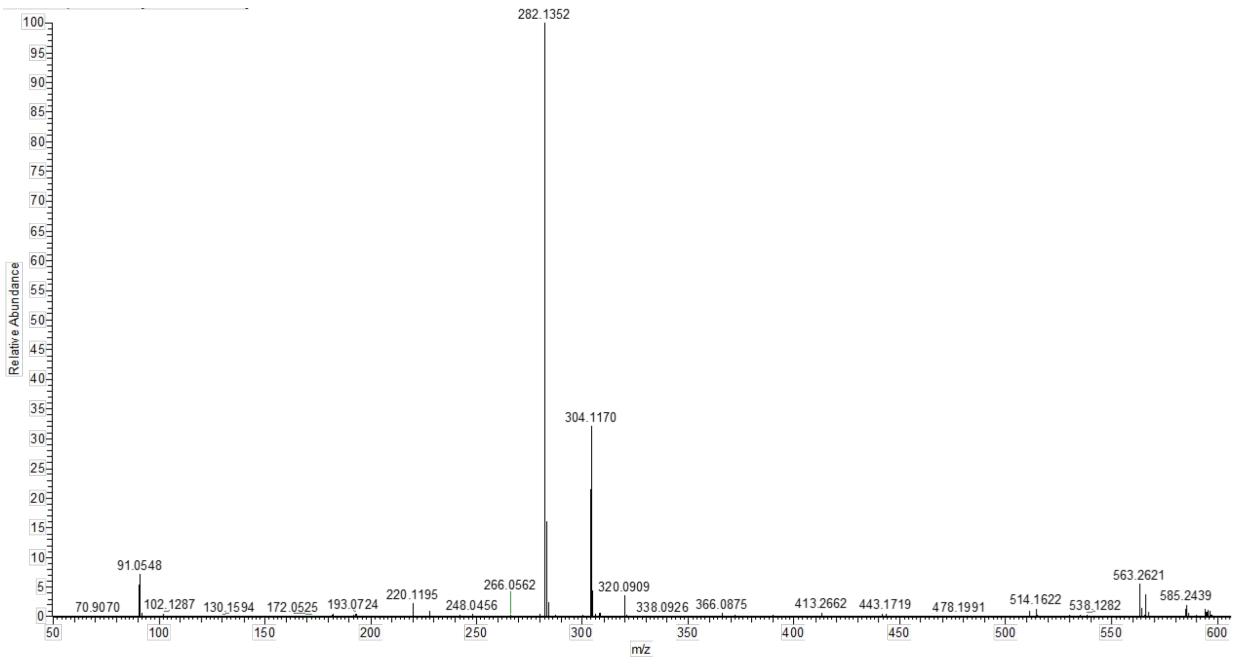

Figure S5. Spectroscopic data for compound **6**: (**a**) ^{1}H NMR (500.13 MHz, DMSO-d_6), (**b**) $^{13}C\{^{1}H\}$ NMR (125.77 MHz, DMSO-d_6), and (**c**) HRMS (ESI).

(a)

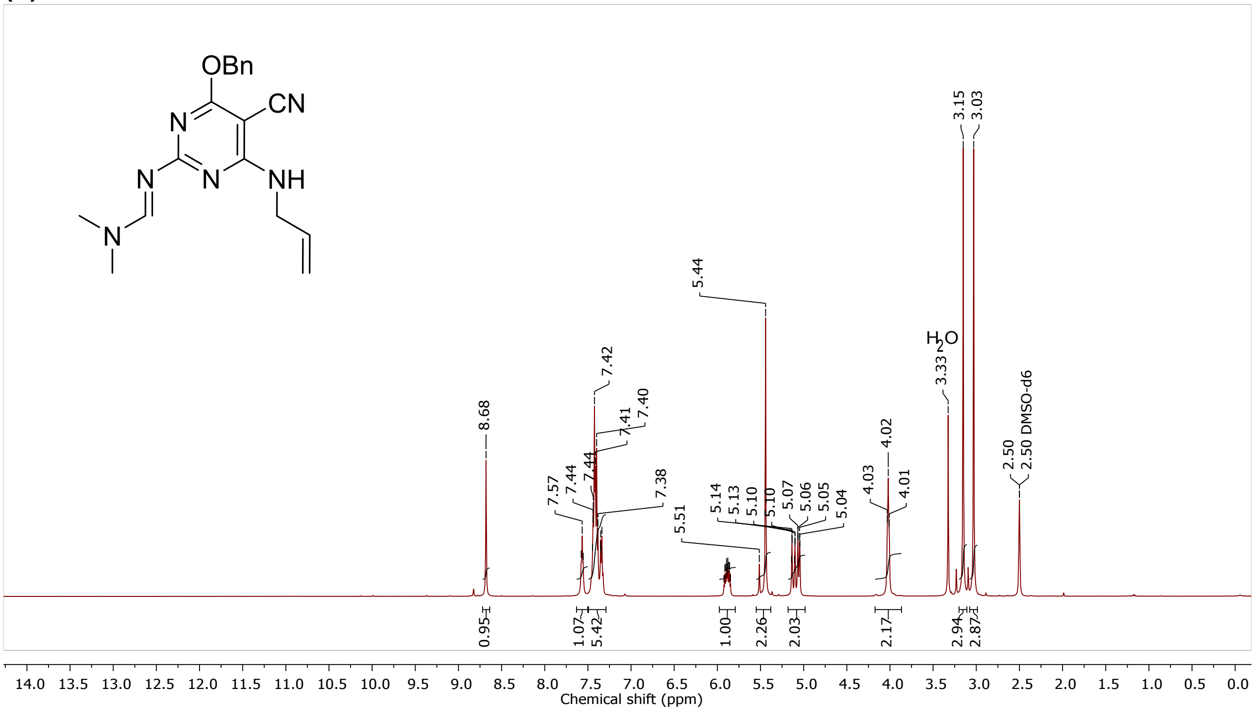
OBn
CN
N
N
N
NH
N
H2O
DMSO-d6
8.68
7.57
7.44
7.44
7.42
7.41
7.40
7.38
5.51
5.44
5.14
5.13
5.10
5.10
5.07
5.06
5.05
5.04
4.03
4.02
4.01
3.33
3.15
3.03
2.50
2.50 DMSO-d6
0.95
1.07
5.42
1.00
2.26
2.03
2.17
2.94
2.87
14.0 13.5 13.0 12.5 12.0 11.5 11.0 10.5 10.0 9.5 9.0 8.5 8.0 7.5 7.0 6.5 6.0 5.5 5.0 4.5 4.0 3.5 3.0 2.5 2.0 1.5 1.0 0.5 0.0
Chemical shift (ppm)

(b)

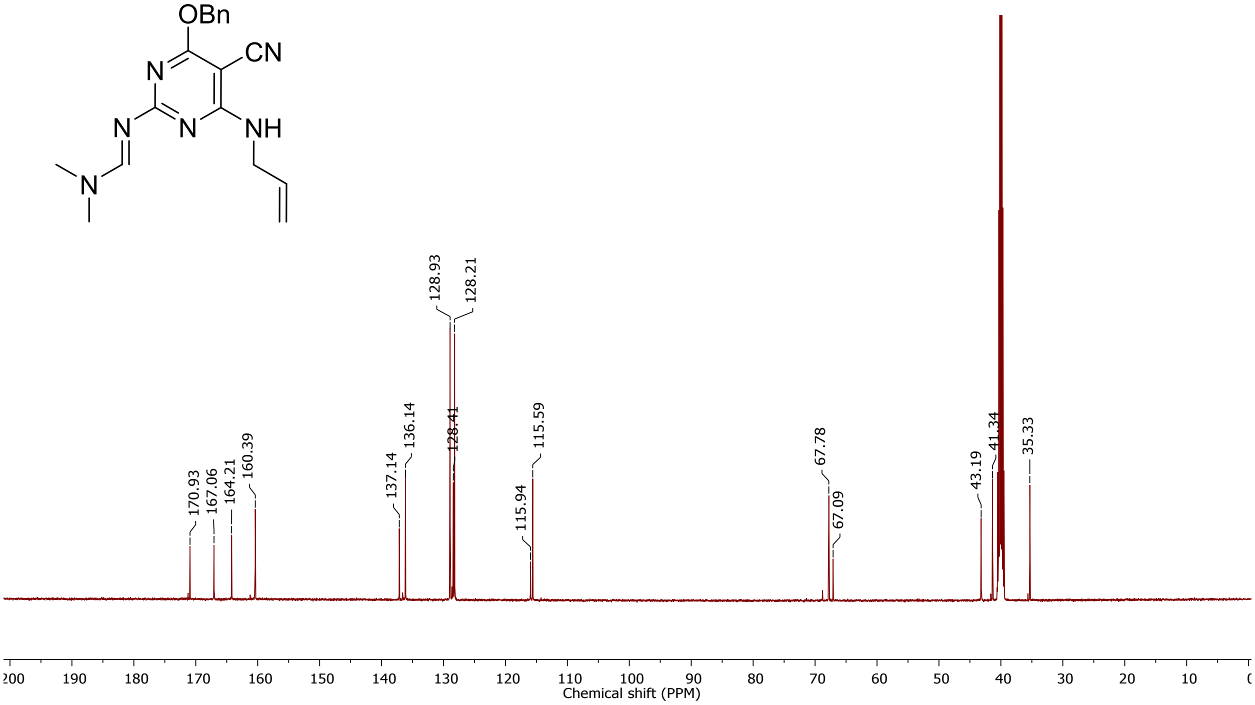
OBn
CN
N
N
N
NH
N
170.93
167.06
164.21
160.39
137.14
136.14
128.93
128.41
128.21
115.94
115.59
67.78
67.09
43.19
41.34
35.33
200 190 180 170 160 150 140 130 120 110 100 90 80 70 60 50 40 30 20 10 0
Chemical shift (PPM)

(c)

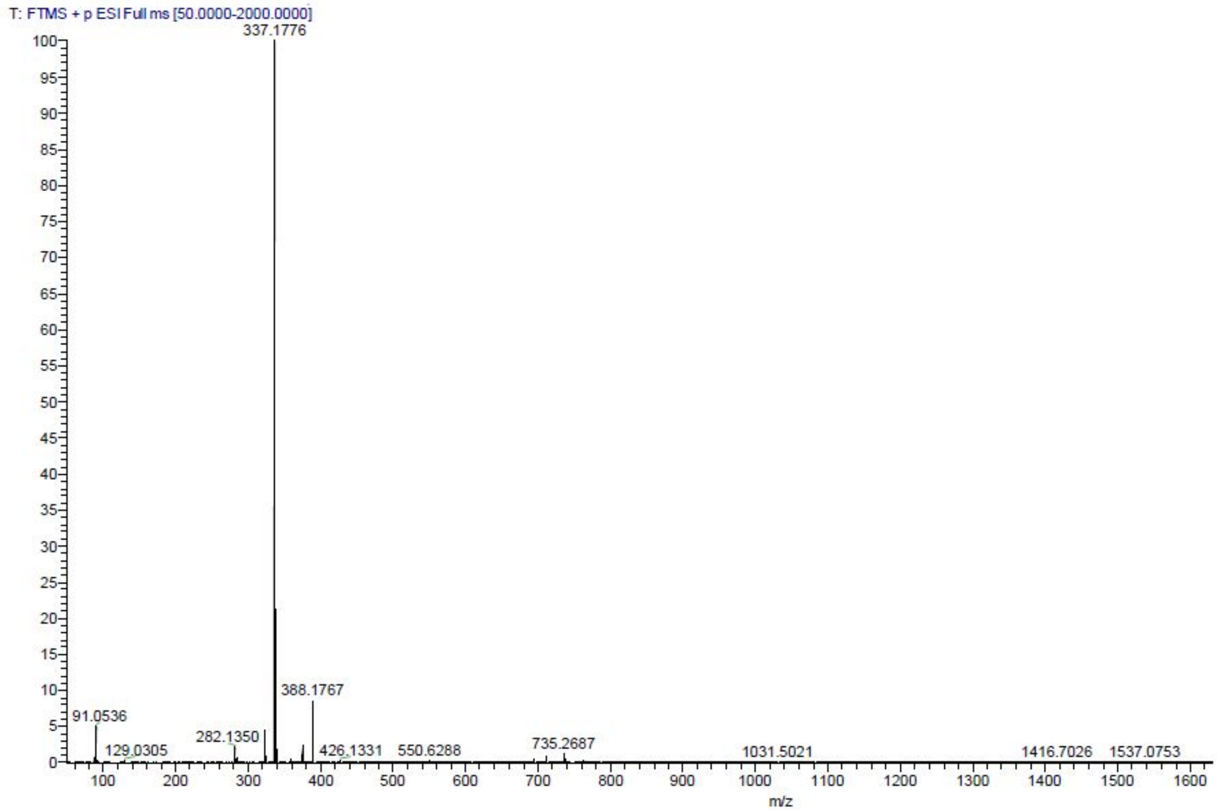

Figure S6. Spectroscopic data for compound **7**: (**a**) ^{1}H NMR (500.13 MHz, DMSO-d_6), (**b**) ^{13}C{^{1}H} NMR (125.77 MHz, DMSO-d_6), and (**c**) HRMS (ESI).

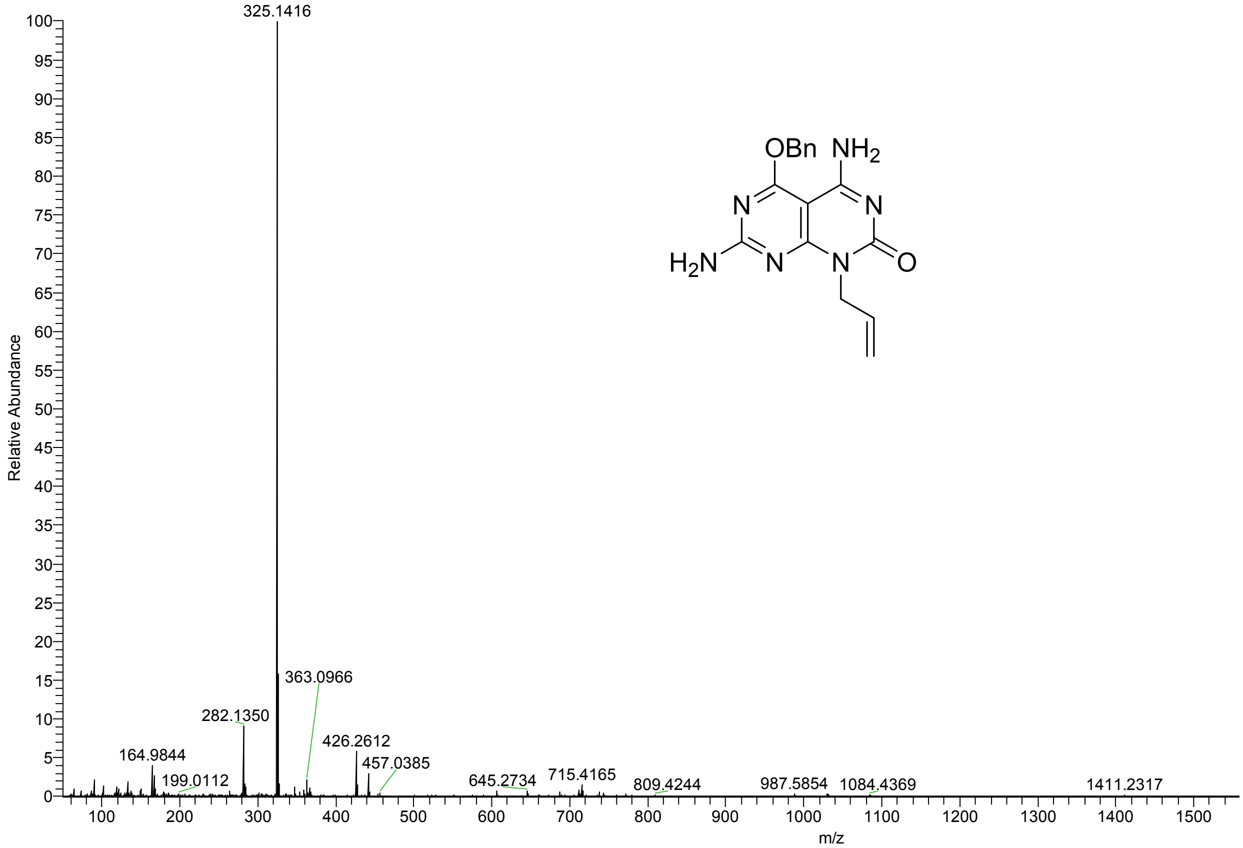

Figure S7. Spectroscopic data for compound **8:** HRMS (ESI).

(a)

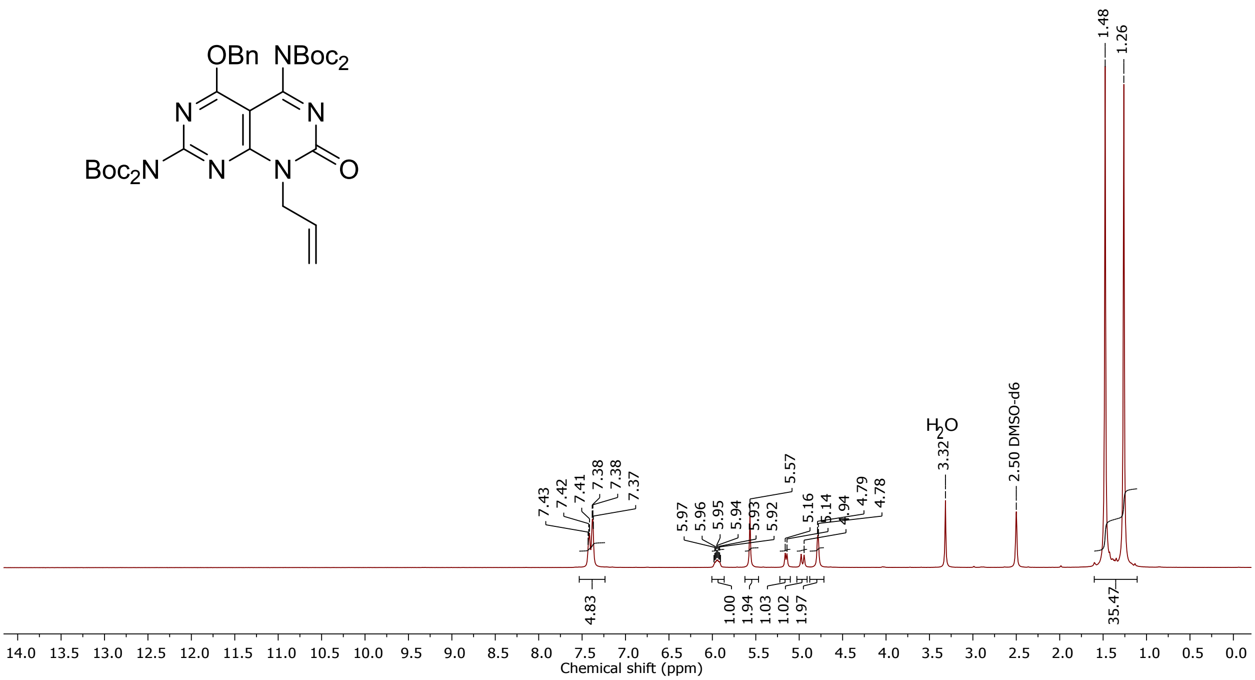
OBn
NBoc2
Boc2N
N
O
7.43
7.42
7.41
7.38
7.38
7.37
5.97
5.96
5.95
5.94
5.93
5.92
5.57
5.16
5.14
4.94
4.79
4.78
H2O
3.32
2.50 DMSO-d6
1.48
1.26
4.83
1.00
1.94
1.03
1.02
1.97
35.47
14.0 13.5 13.0 12.5 12.0 11.5 11.0 10.5 10.0 9.5 9.0 8.5 8.0 7.5 7.0 6.5 6.0 5.5 5.0 4.5 4.0 3.5 3.0 2.5 2.0 1.5 1.0 0.5 0.0
Chemical shift (ppm)

(b)

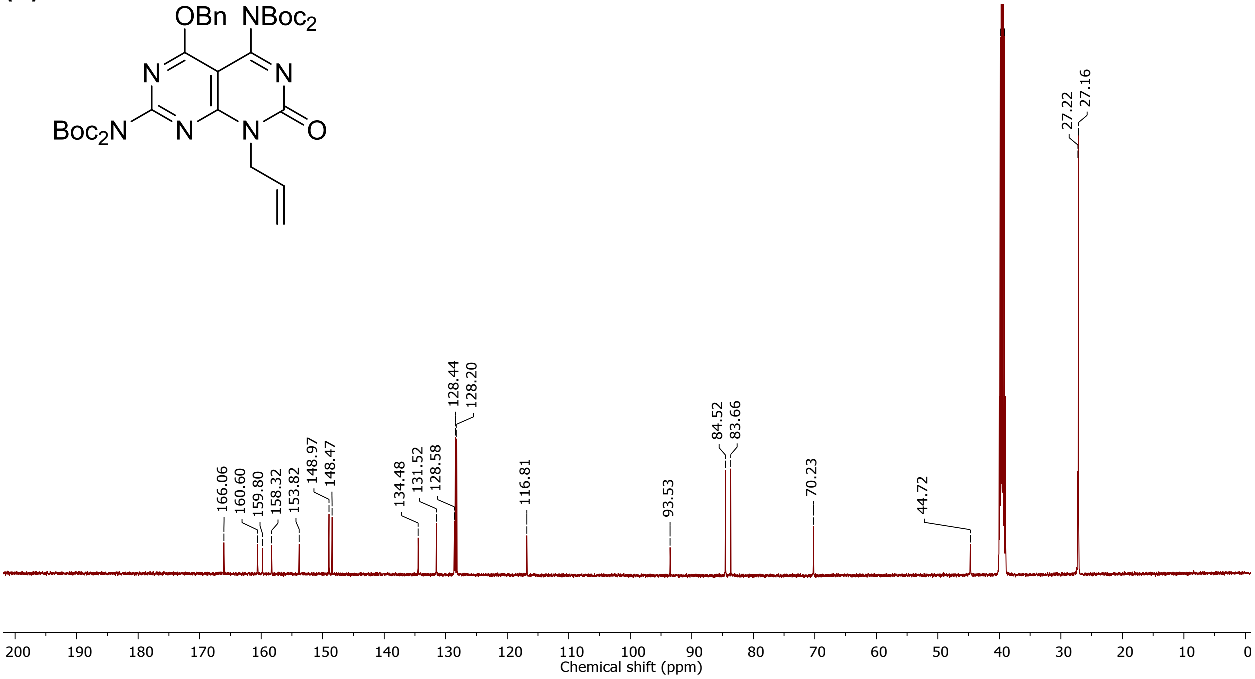
OBn
NBoc2
Boc2N
N
O
166.06
160.60
159.80
158.32
153.82
148.97
148.47
134.48
131.52
128.58
128.44
128.20
116.81
93.53
84.52
83.66
70.23
44.72
27.22
27.16
200 190 180 170 160 150 140 130 120 110 100 90 80 70 60 50 40 30 20 10 0
Chemical shift (ppm)

(c)

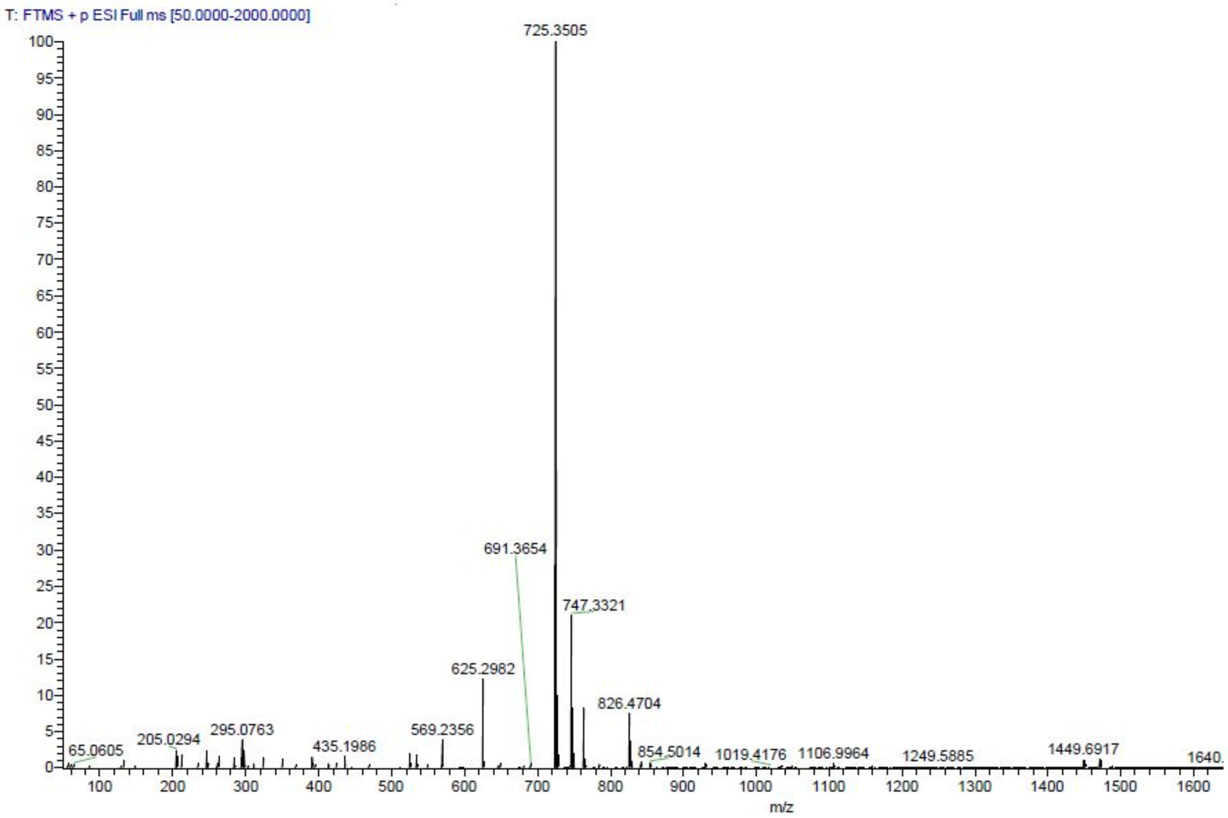

Figure S8. Spectroscopic data for compound **9**: (**a**) ^{1}H NMR (500.13 MHz, DMSO-d_6), (**b**) ^{13}C{^{1}H} NMR (125.77 MHz, DMSO-d_6), and (**c**) HRMS (ESI).

(a)

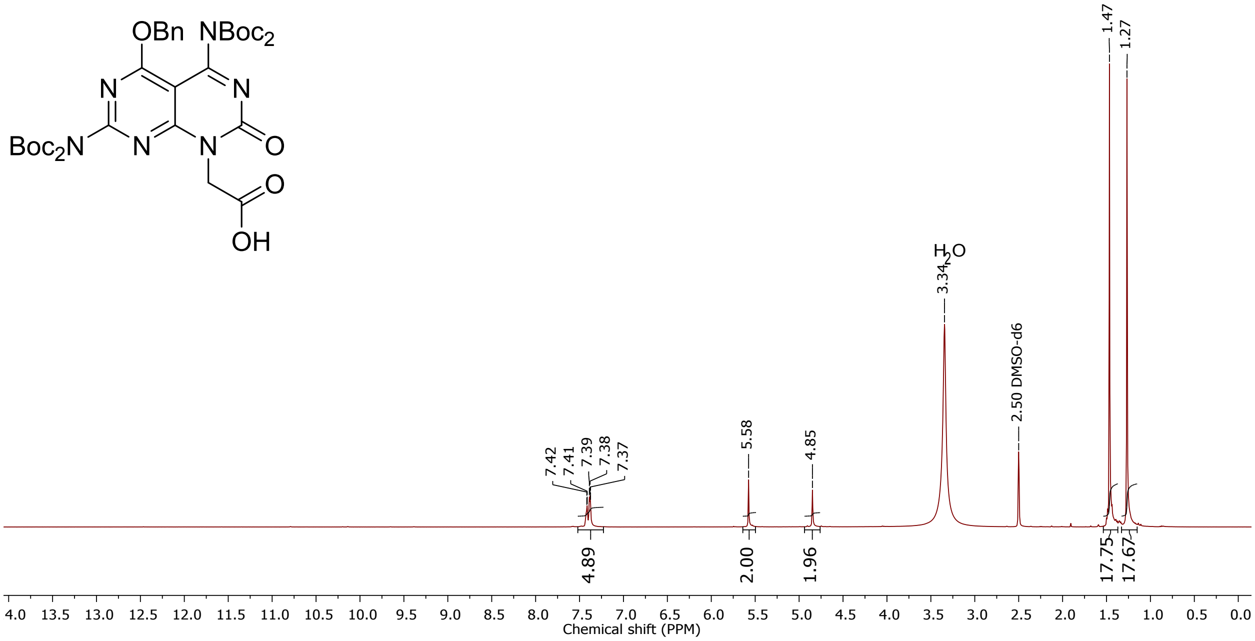
OBn NBoc2
N N
Boc2N N N O
O
OH
7.42 7.41 7.39 7.38 7.37
5.58
4.85
H2O 3.34
2.50 DMSO-d6
1.47
1.27
4.89
2.00
1.96
17.75
17.67
Chemical shift (PPM)

(b)

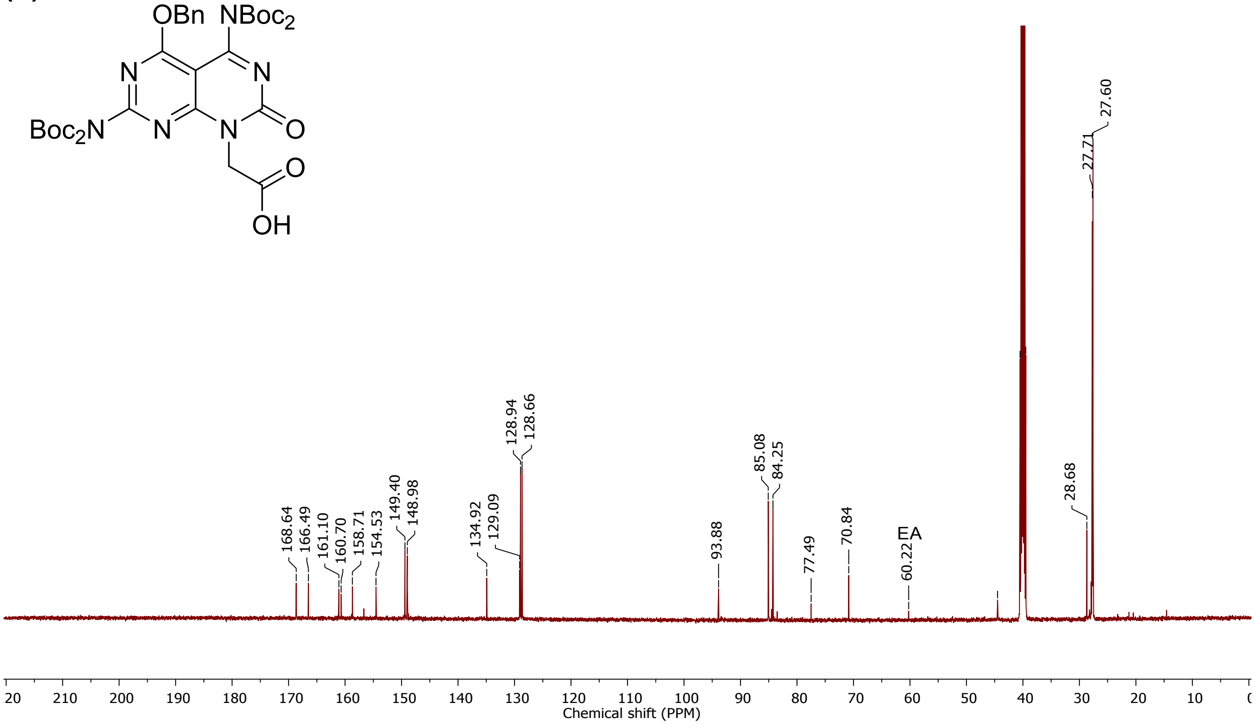
OBn NBoc2
N N
Boc2N N N O
O
OH
168.64 166.49 161.10 160.70 158.71 154.53 149.40 148.98
134.92 129.09 128.94 128.66
93.88
85.08 84.25
77.49
70.84
EA 60.22
28.68
27.71 27.60
Chemical shift (PPM)

(c)

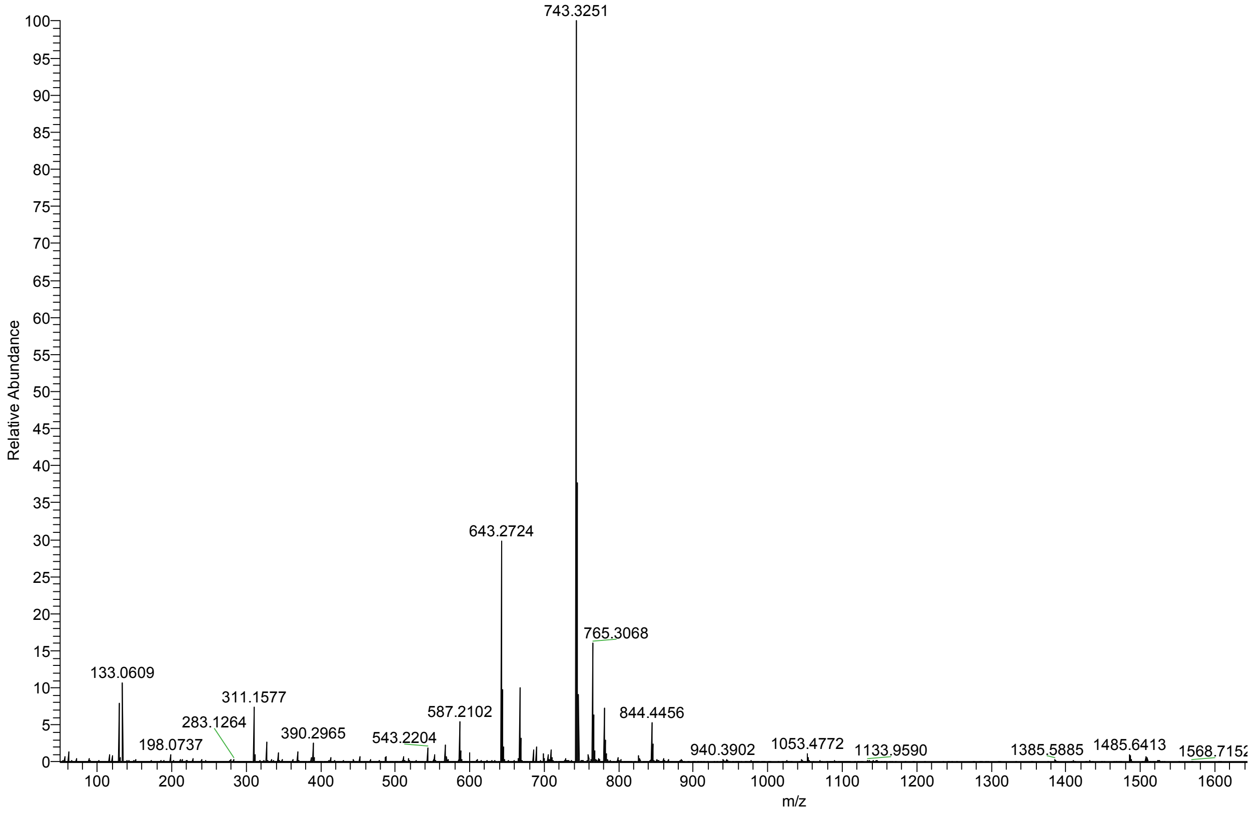

Figure S9. Spectroscopic data for compound **1/E**: (**a**) ^1H NMR (500.13 MHz, DMSO-d_6), (**b**) $^{13}C\{^1H\}$ NMR (125.77 MHz, DMSO-d_6), and (**c**) HRMS (ESI).

(a)

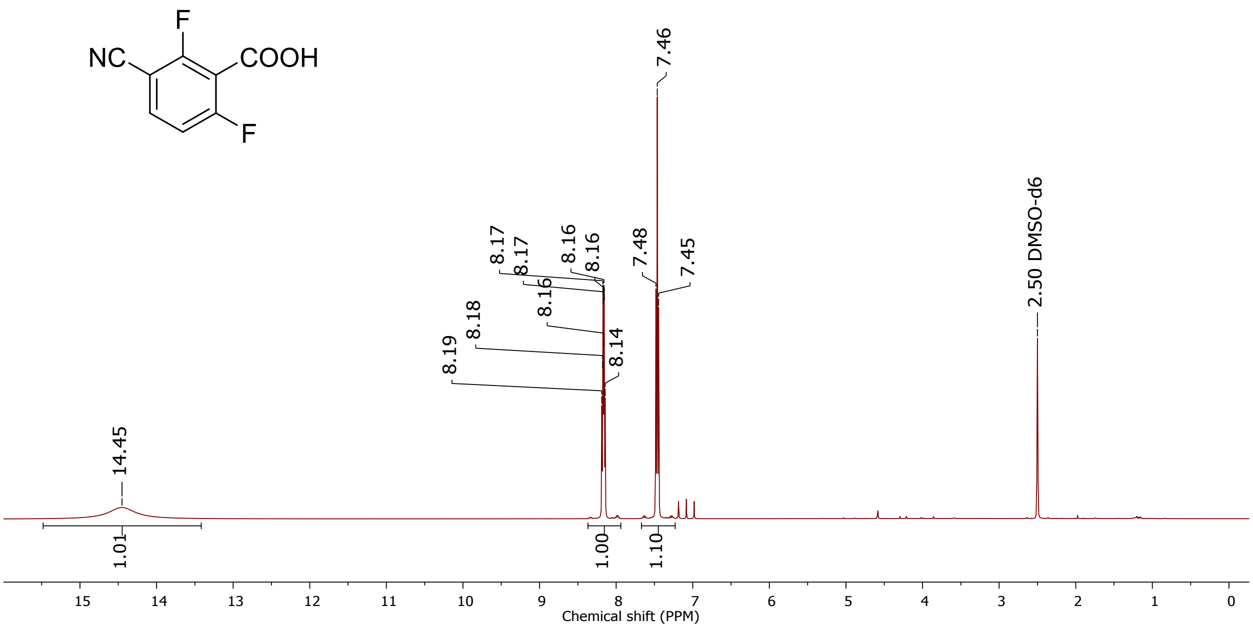

NC
F
COOH
F
14.45
8.19
8.18
8.17
8.17
8.16
8.16
8.16
8.14
7.48
7.46
7.45
2.50 DMSO-d6
1.01
1.00
1.10
15 14 13 12 11 10 9 8 7 6 5 4 3 2 1 0
Chemical shift (PPM)

(b)

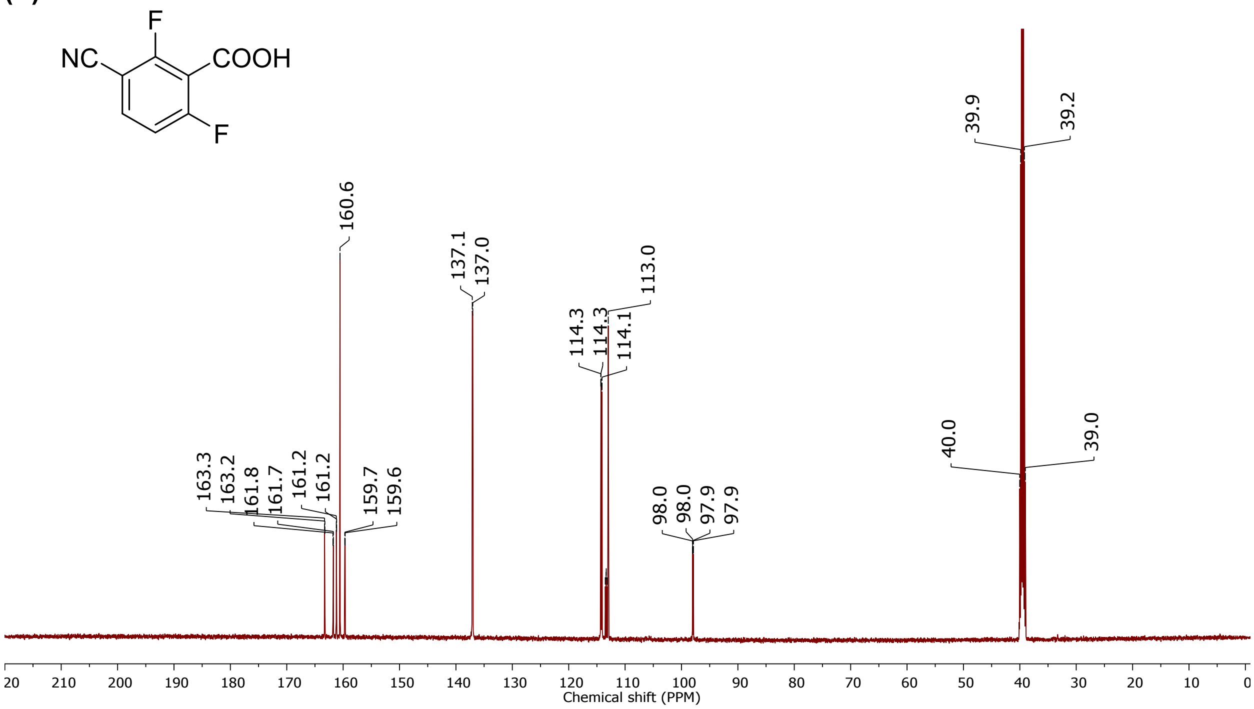

NC
F
COOH
F
163.3
163.2
161.8
161.7
161.2
161.2
160.6
159.7
159.6
137.1
137.0
114.3
114.3
114.1
113.0
98.0
98.0
97.9
97.9
40.0
39.9
39.2
39.0
20 210 200 190 180 170 160 150 140 130 120 110 100 90 80 70 60 50 40 30 20 10 0
Chemical shift (PPM)

(c)

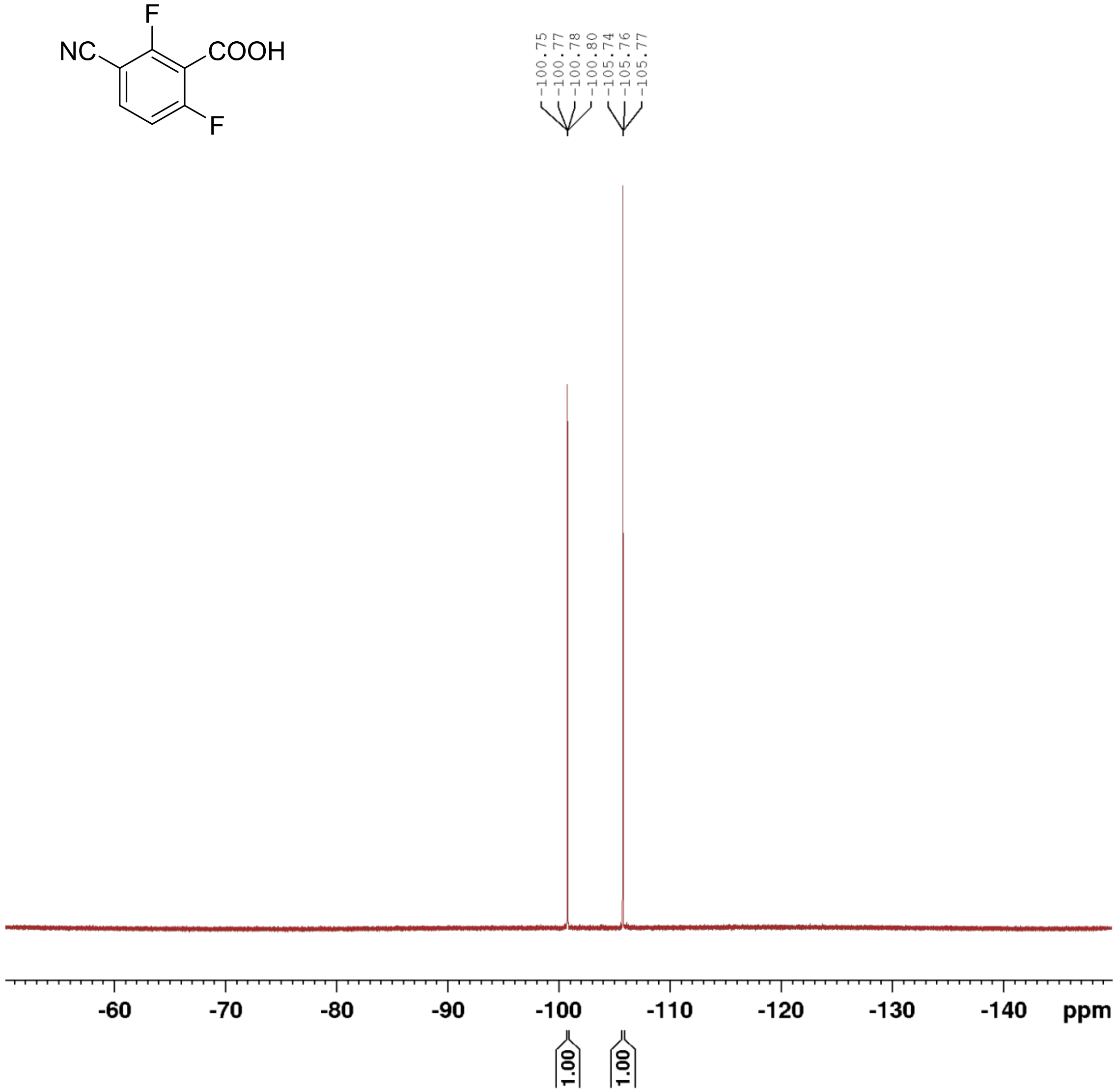

Figure S10. Spectroscopic data for compound **10**: (**a**) ^{1}H NMR (500.13 MHz, DMSO-d_6), (**b**) $^{13}C\{^{1}H\}$ NMR (125.77 MHz, DMSO-d_6), and **(c)** ^{19}F NMR (470.59 MHz, DMSO-d_6)

(a)

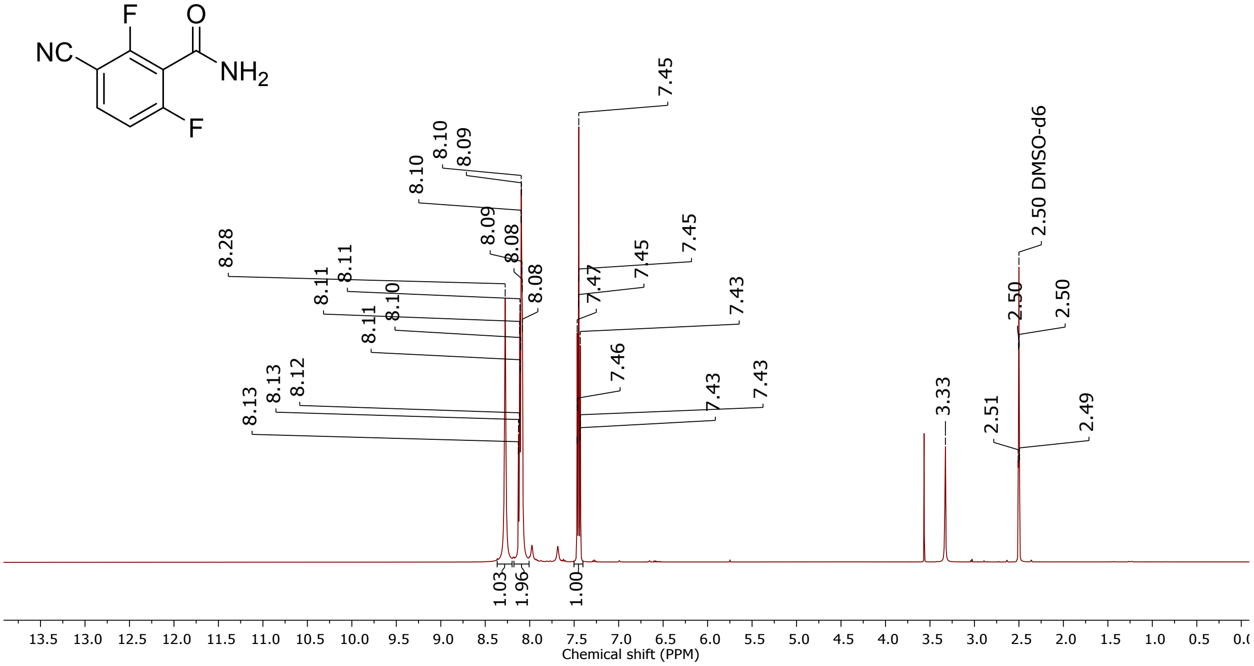
NC
F
O
NH2
F
7.45
8.10
8.10
8.09
2.50 DMSO-d6
8.28
8.09
8.08
8.11
8.11
8.08
7.47
7.45
7.45
7.43
8.11
8.10
2.50
2.50
7.46
8.13
8.13
8.12
7.43
7.43
3.33
2.51
2.49
1.03
1.96
1.00
13.5 13.0 12.5 12.0 11.5 11.0 10.5 10.0 9.5 9.0 8.5 8.0 7.5 7.0 6.5 6.0 5.5 5.0 4.5 4.0 3.5 3.0 2.5 2.0 1.5 1.0 0.5
Chemical shift (PPM)

(b)

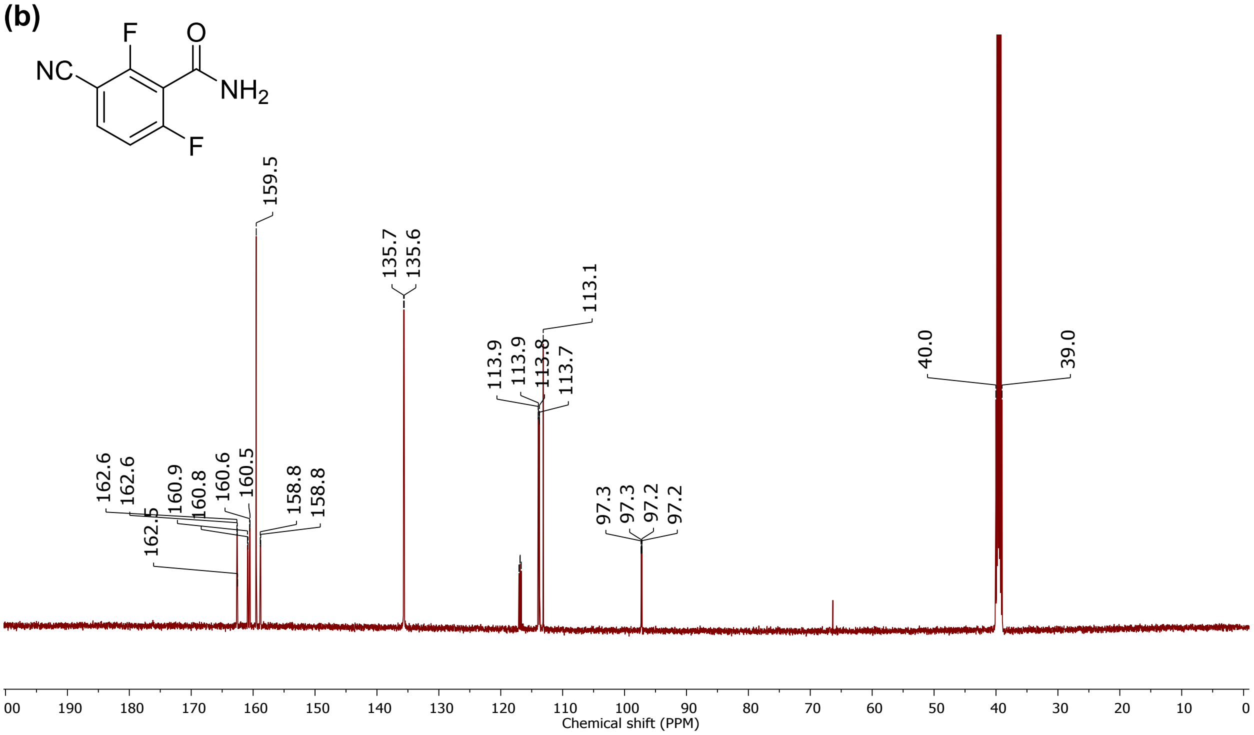
NC
F
O
NH2
F
159.5
135.7
135.6
113.1
113.9
113.9
113.8
113.7
40.0
39.0
162.6
162.6
160.9
160.8
160.6
160.5
162.5
158.8
158.8
97.3
97.3
97.2
97.2
190 180 170 160 150 140 130 120 110 100 90 80 70 60 50 40 30 20 10 0
Chemical shift (PPM)

(c)

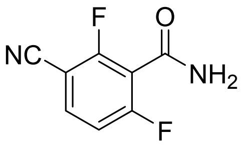
NC
F
O
NH2
F

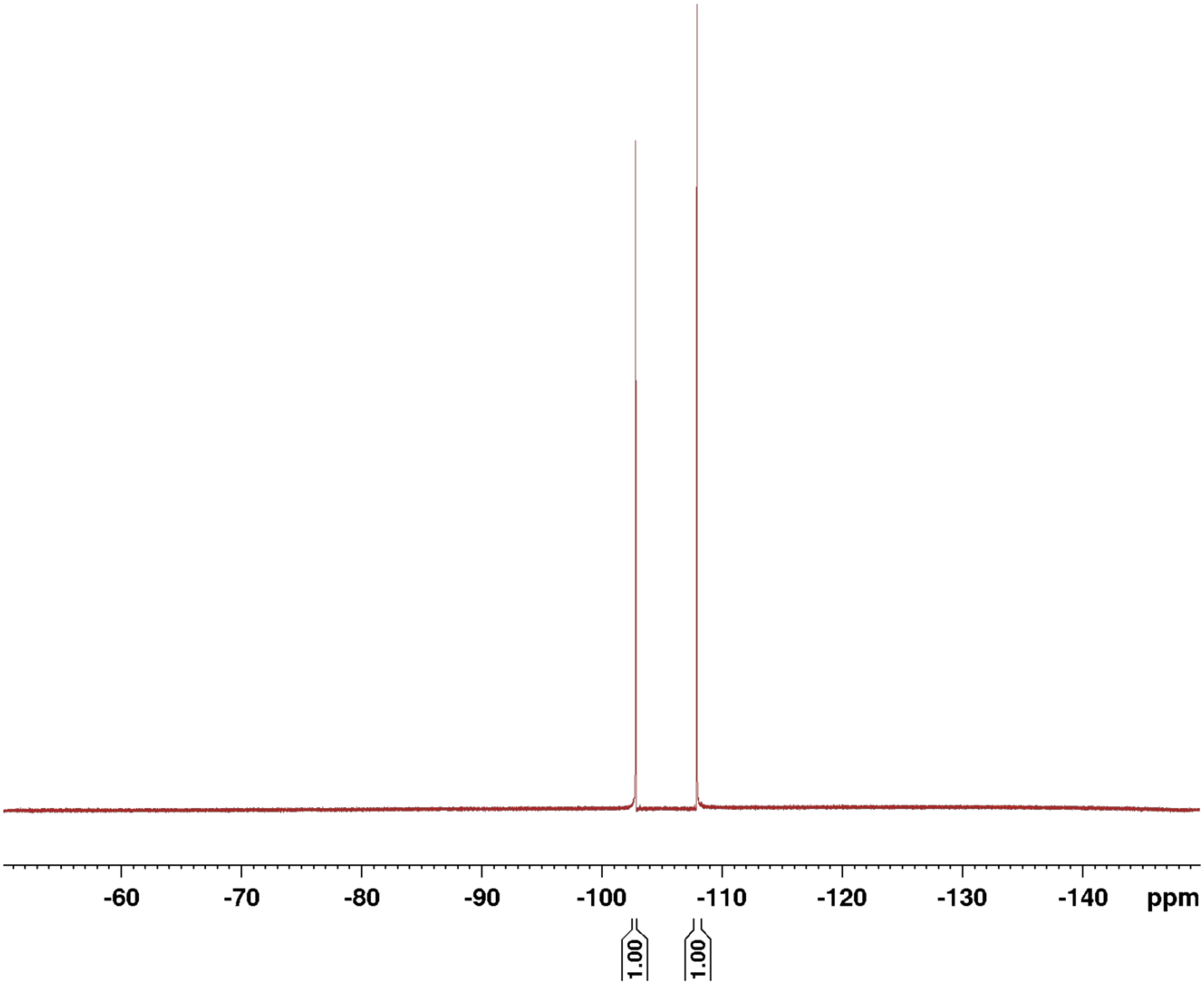
-102.79
-102.80
-102.81
-102.82
-102.82
-102.84
-107.90
-107.92
-107.94
-60
-70
-80
-90
-100
-110
-120
-130
-140
ppm
1.00
1.00

(d)

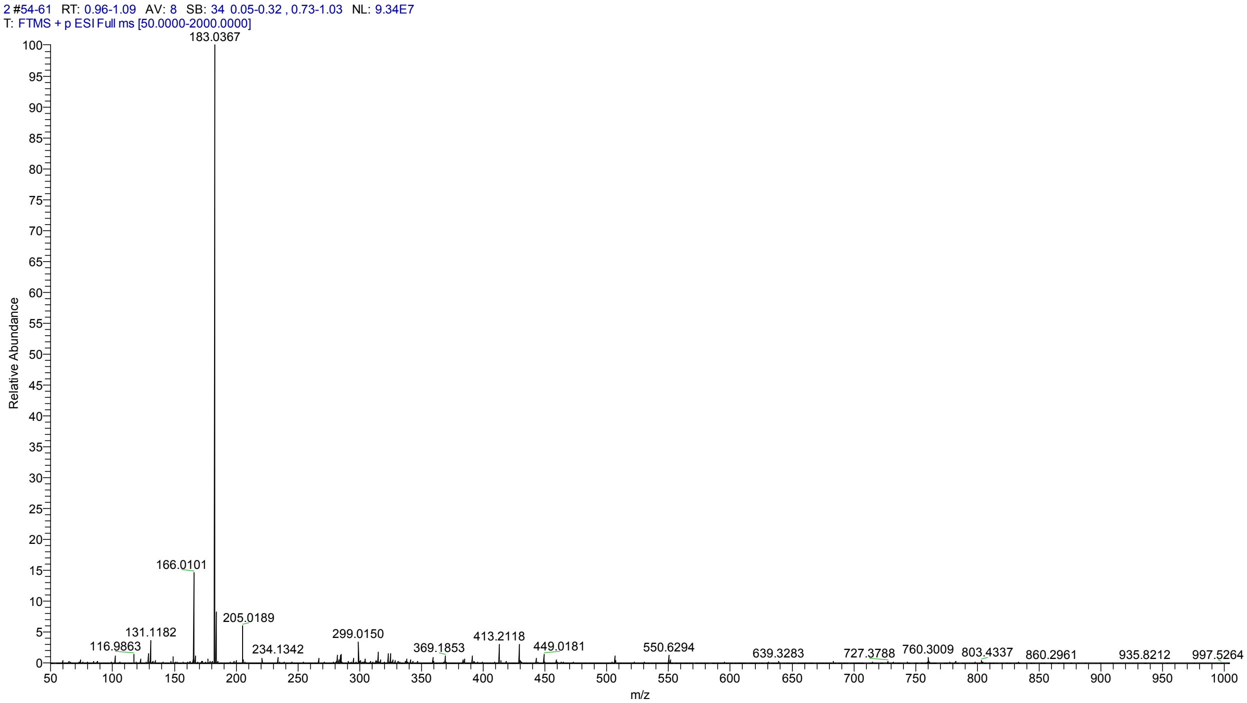

Figure S11. Spectroscopic data for compound **11**: (**a**) ^{1}H NMR (500.13 MHz, DMSO-d_6), (**b**) $^{13}C\{^{1}H\}$ NMR (125.77 MHz, DMSO-d_6), **(c)** ^{19}F NMR (470.59 MHz, DMSO-d_6), and (**d**) HRMS (ESI).

(a)

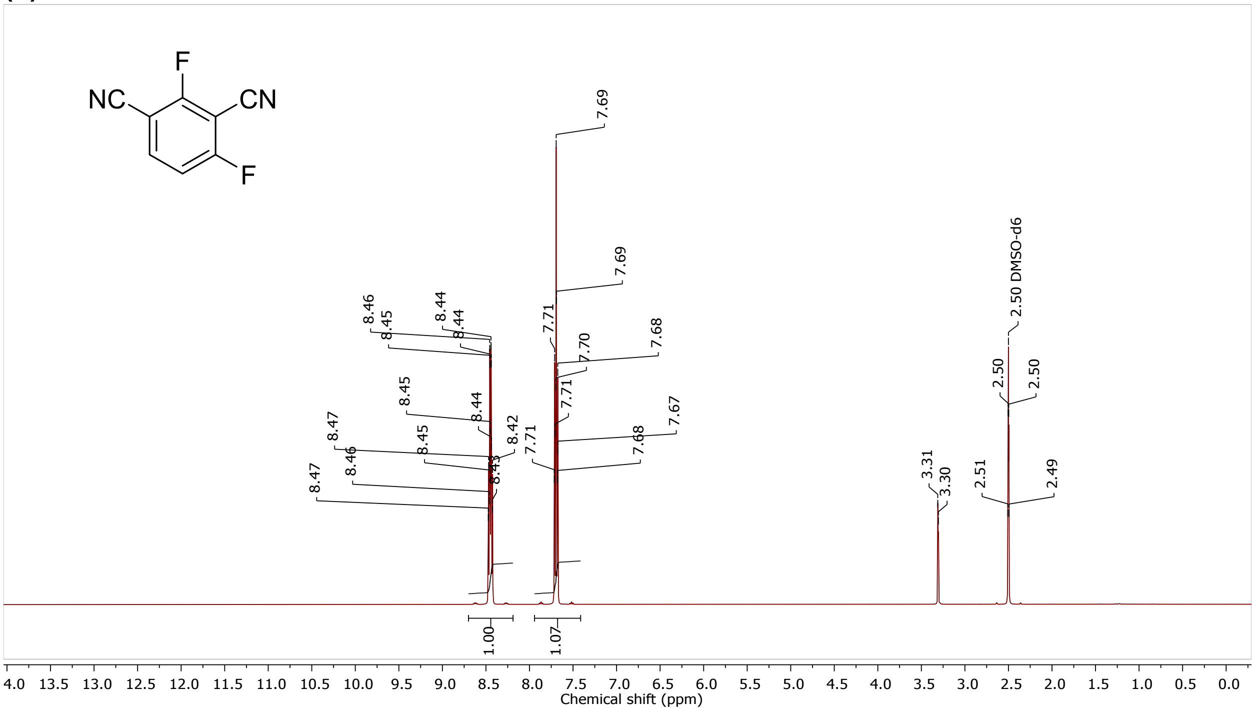
F
NC
CN
F
7.69
7.69
8.46
8.45
8.44
8.44
7.71
7.70
7.68
8.45
8.44
7.71
8.47
8.46
8.45
8.42
8.43
7.71
7.68
7.67
8.47
3.31
3.30
2.51
2.50
2.50
2.49
2.50 DMSO-d6
1.00
1.07
4.0 13.5 13.0 12.5 12.0 11.5 11.0 10.5 10.0 9.5 9.0 8.5 8.0 7.5 7.0 6.5 6.0 5.5 5.0 4.5 4.0 3.5 3.0 2.5 2.0 1.5 1.0 0.5 0.0
Chemical shift (ppm)

(b)

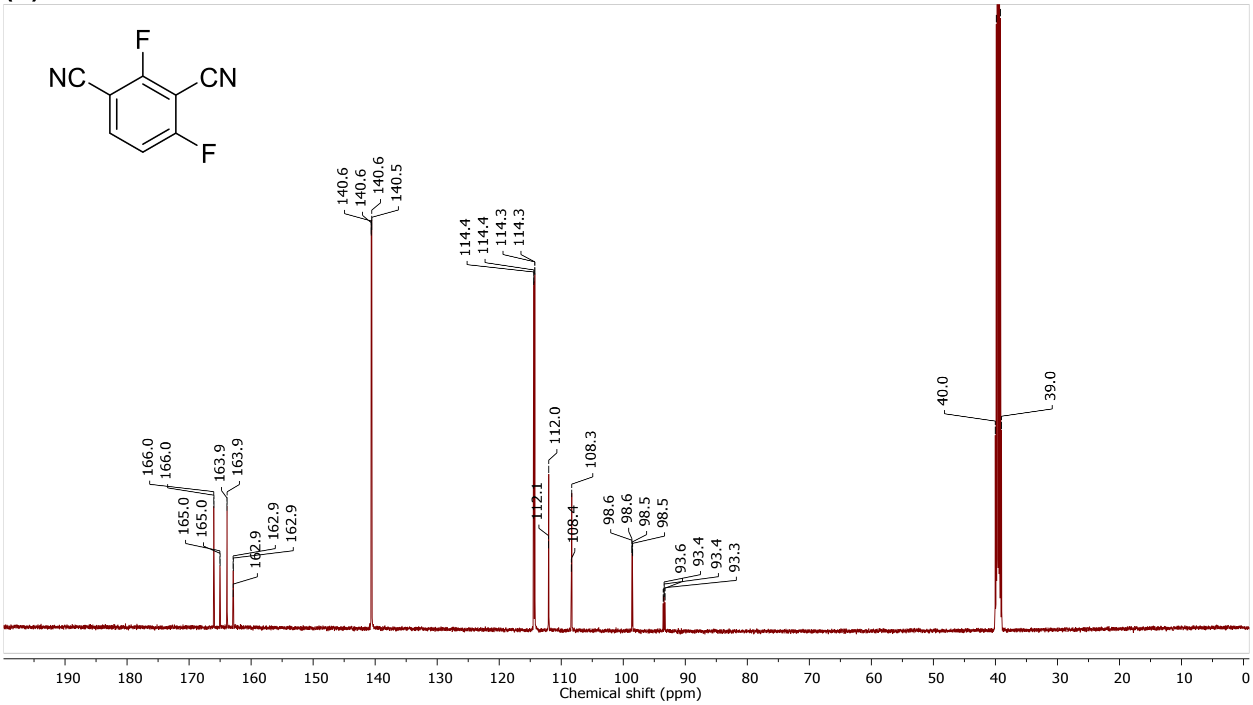
F
NC
CN
F
140.6
140.6
140.6
140.5
114.4
114.4
114.3
114.3
112.0
112.1
108.3
108.4
166.0
166.0
165.0
165.0
163.9
163.9
162.9
162.9
162.9
98.6
98.6
98.5
98.5
93.6
93.4
93.4
93.3
40.0
39.0
190 180 170 160 150 140 130 120 110 100 90 80 70 60 50 40 30 20 10 0
Chemical shift (ppm)

(c)

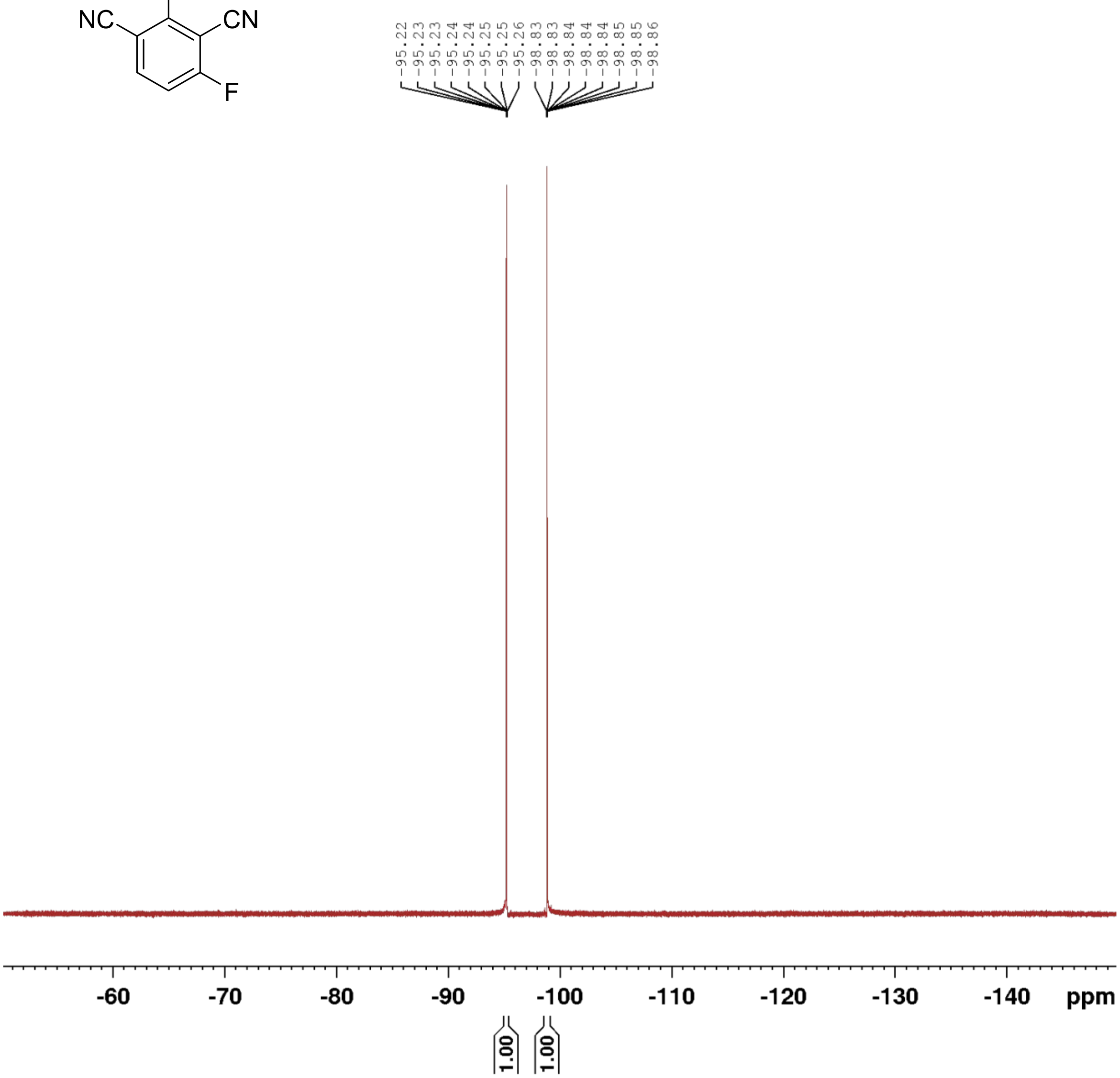

Figure S12. Spectroscopic data for compound **12:** (**a**) ^{1}H NMR (500.13 MHz, DMSO-d_6), (**b**) $^{13}C\{^{1}H\}$ NMR (125.77 MHz, DMSO-d_6), and **(c)** ^{19}F NMR (470.59 MHz, DMSO-d_6).

(a)

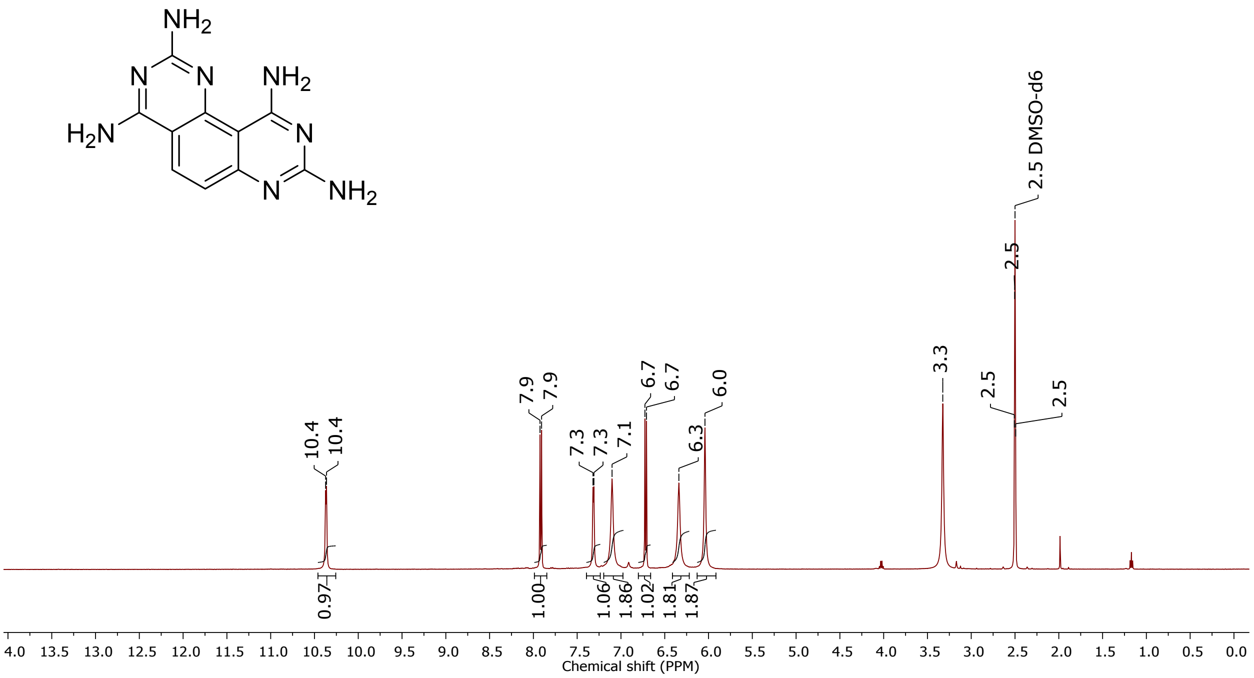
NH2
N
N
NH2
H2N
N
N
NH2
2.5 DMSO-d6
2.5
3.3
2.5
2.5
10.4
10.4
7.9
7.9
7.3
7.3
7.1
6.7
6.7
6.3
6.0
0.97
1.00
1.06
1.86
1.02
1.81
1.87
Chemical shift (PPM)

(b)

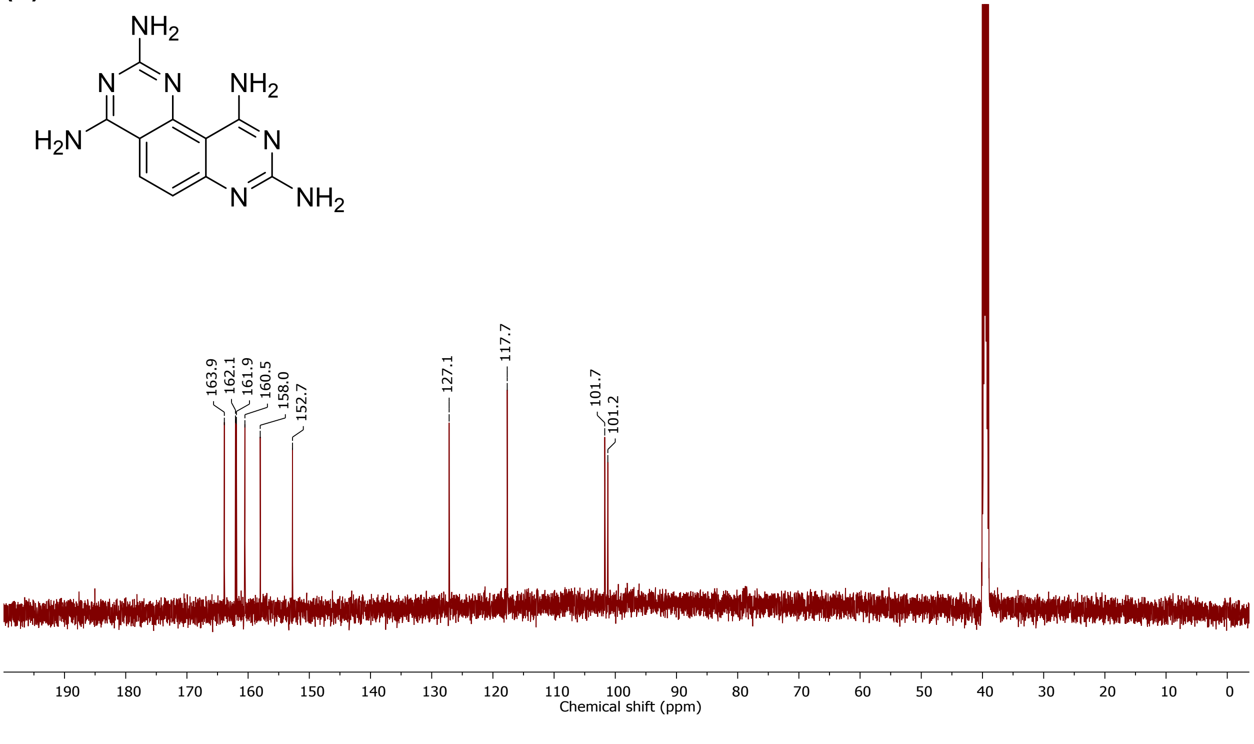
NH2
N
N
NH2
H2N
N
N
NH2
163.9
162.1
161.9
160.5
158.0
152.7
127.1
117.7
101.7
101.2
Chemical shift (ppm)

(c)

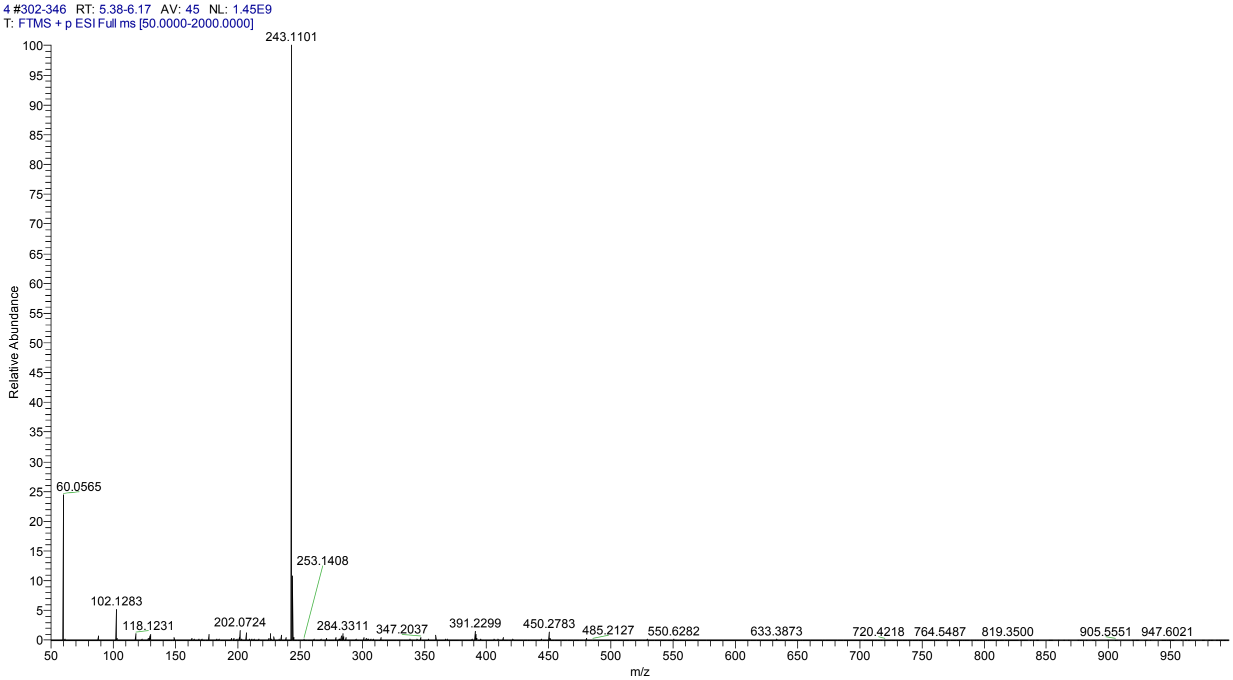

Figure S13. Spectroscopic data for compound **13:** (**a**) ^{1}H NMR (500.13 MHz, DMSO-d_6), (**b**) $^{13}C\{^{1}H\}$ NMR (125.77 MHz, DMSO-d_6), and (**c**) HRMS (ESI).

(a)

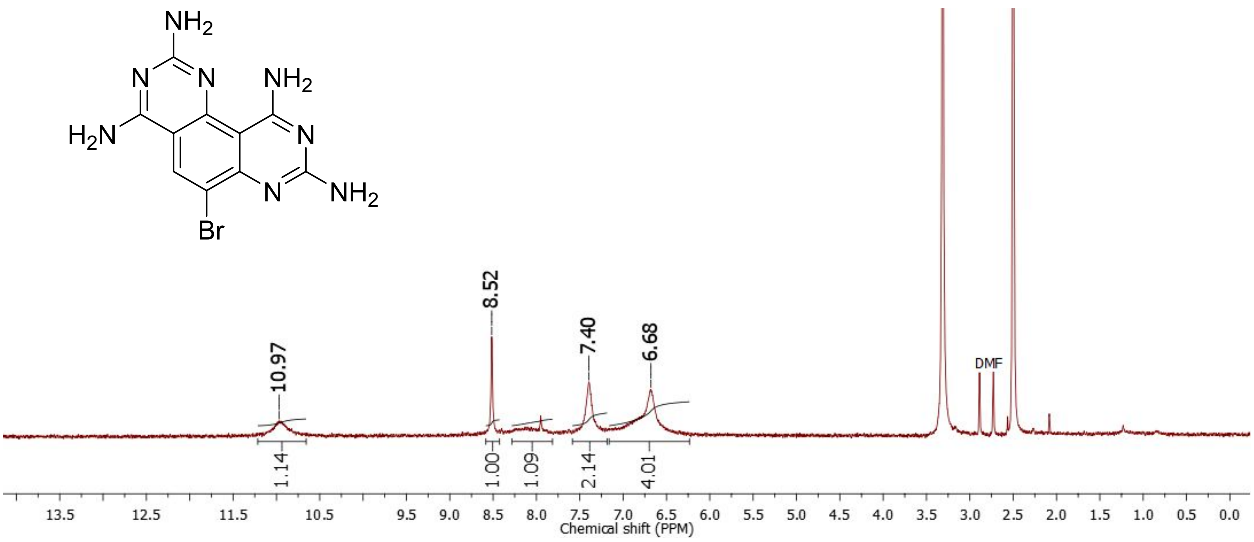
NH2
N
N
NH2
H2N
N
N
NH2
Br
10.97
8.52
7.40
6.68
DMF
1.14
1.00
1.09
2.14
4.01
13.5
12.5
11.5
10.5
9.5
9.0
8.5
8.0
7.5
7.0
6.5
6.0
5.5
5.0
4.5
4.0
3.5
3.0
2.5
2.0
1.5
1.0
0.5
0.0
Chemical shift (PPM)

(b)

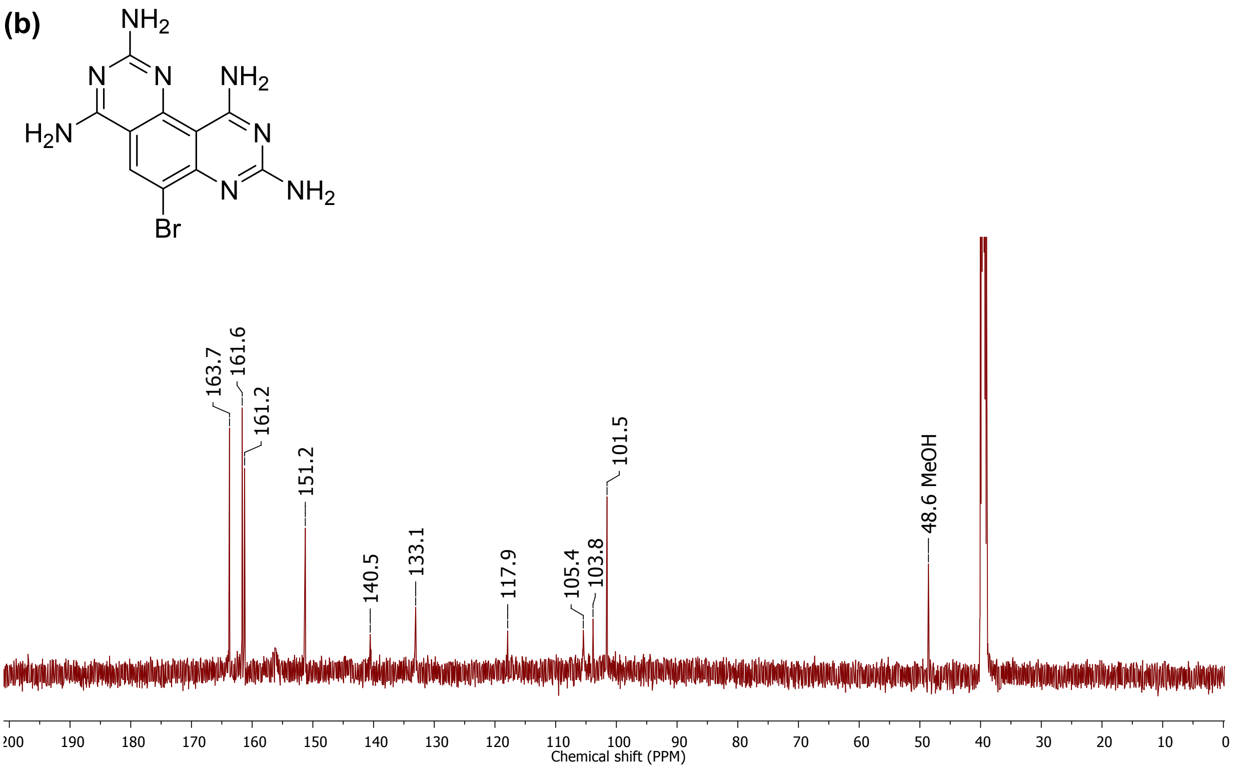
NH2
N
N
NH2
H2N
N
N
NH2
Br
163.7
161.6
161.2
151.2
140.5
133.1
117.9
105.4
103.8
101.5
48.6 MeOH
200
190
180
170
160
150
140
130
120
110
100
90
80
70
60
50
40
30
20
10
0
Chemical shift (PPM)

(c)

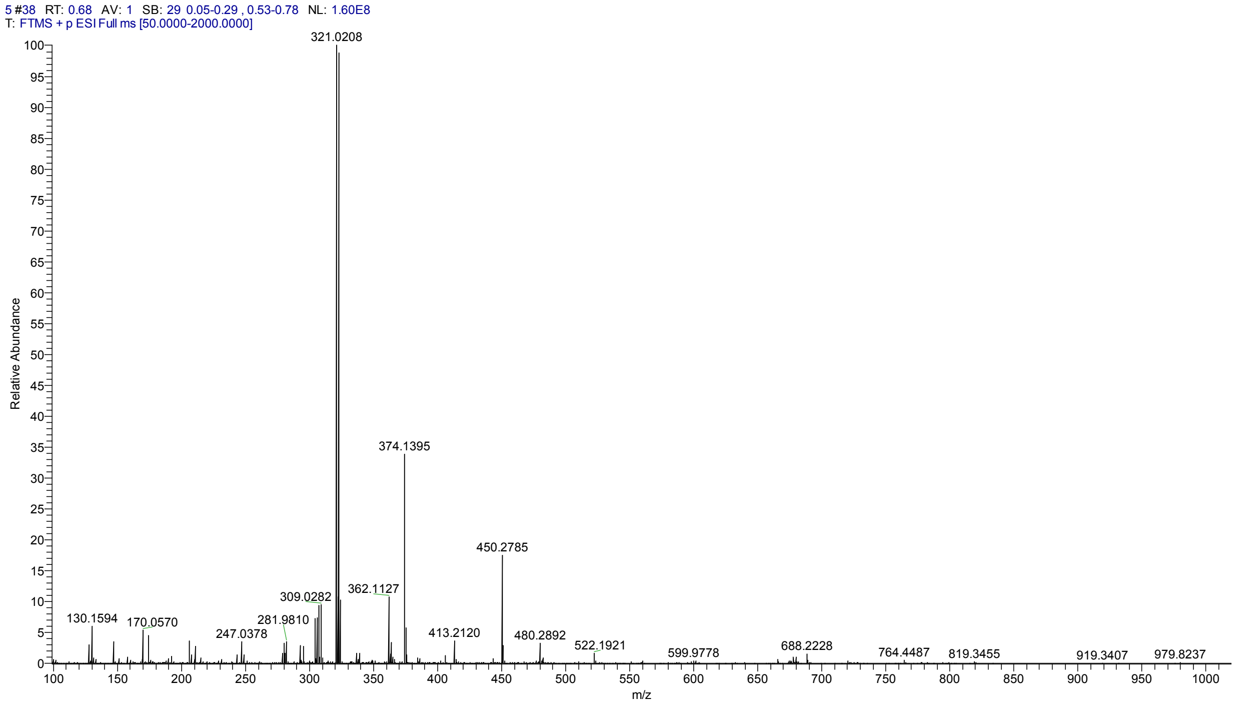

Figure S14. Spectroscopic data for compound **14:** (**a**) ^{1}H NMR (500.13 MHz, DMSO-d_6), (**b**) ^{13}C{^{1}H} NMR (125.77 MHz, DMSO-d_6), and (**c**) HRMS (ESI).

(a)

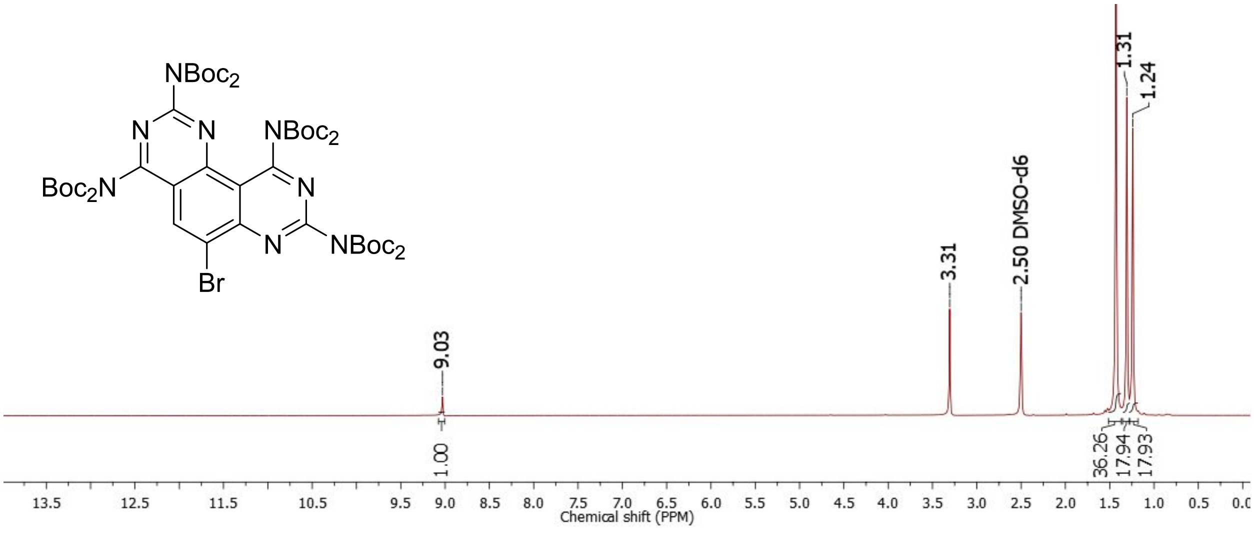
NBoc2
N
N
NBoc2
Boc2N
N
N
NBoc2
Br
9.03
3.31
2.50 DMSO-d6
1.31
1.24
1.00
36.26
17.94
17.93
13.5 12.5 11.5 10.5 9.5 9.0 8.5 8.0 7.5 7.0 6.5 6.0 5.5 5.0 4.5 4.0 3.5 3.0 2.5 2.0 1.5 1.0 0.5 0.0
Chemical shift (PPM)

(b)

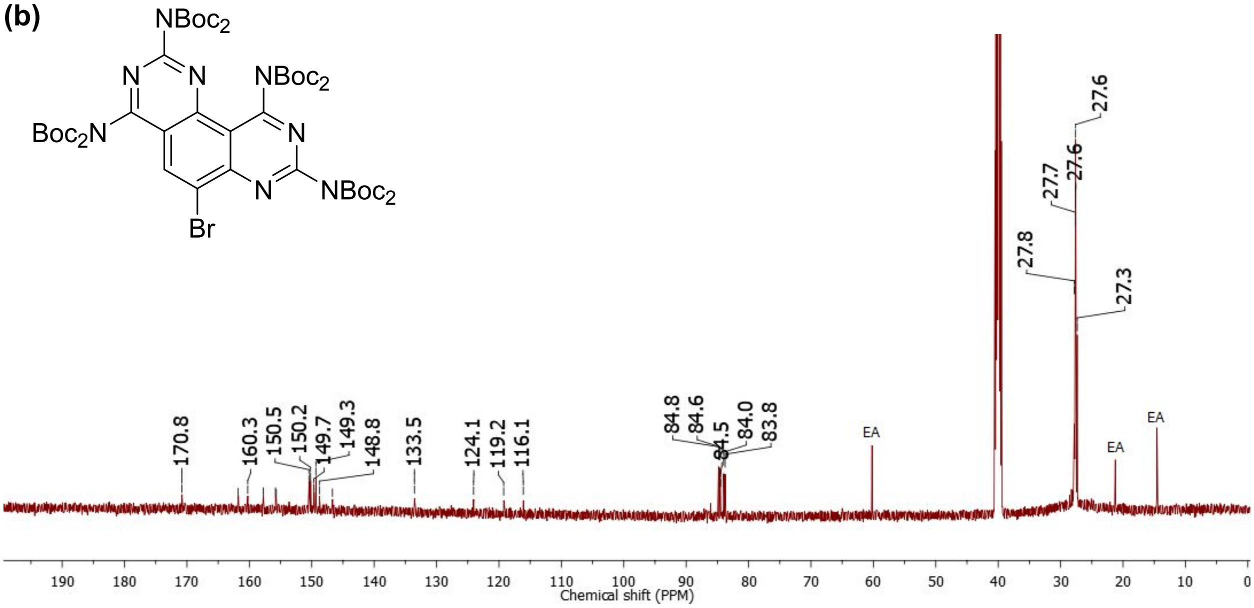
NBoc2
N
N
NBoc2
Boc2N
N
N
NBoc2
Br
170.8
160.3
150.5
150.2
149.7
149.3
148.8
133.5
124.1
119.2
116.1
84.8
84.6
84.5
84.0
83.8
EA
27.8
27.7
27.6
27.6
27.3
EA
EA
190 180 170 160 150 140 130 120 110 100 90 80 70 60 50 40 30 20 10 0
Chemical shift (PPM)

(c)

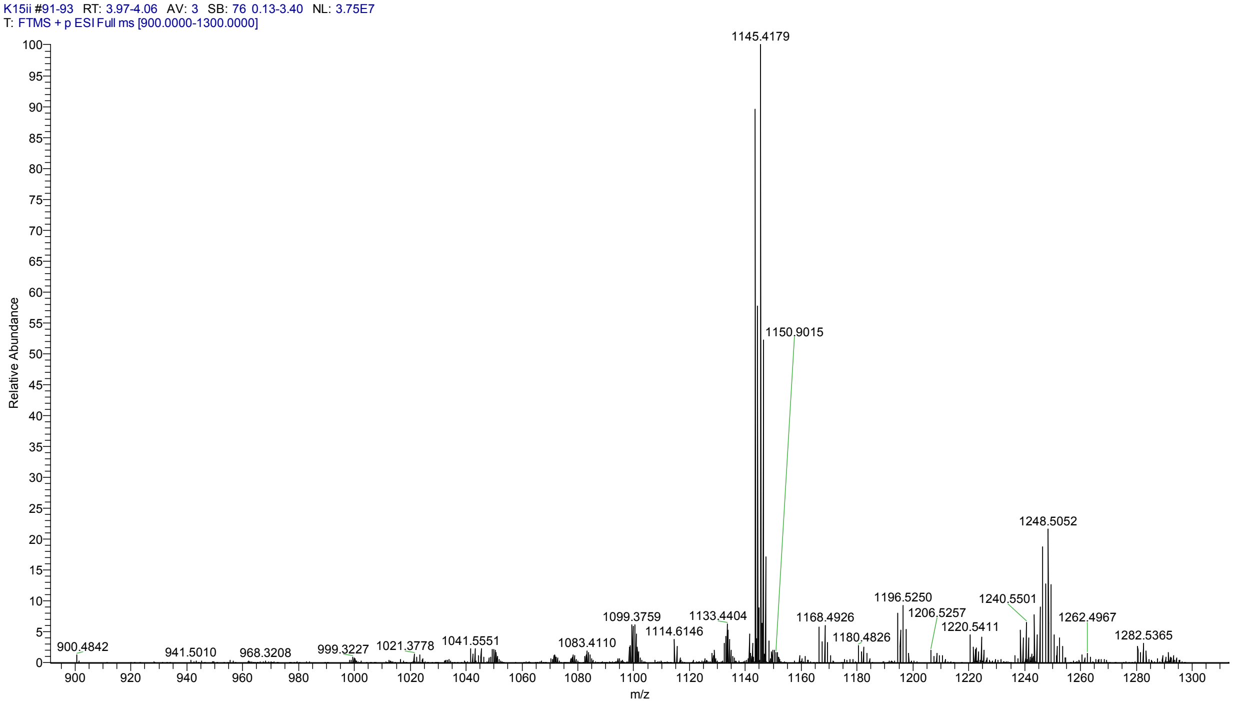

Figure S15. Spectroscopic data for compound **15**: (**a**) ^{1}H NMR (500.13 MHz, DMSO-d_6), (**b**) $^{13}C\{^{1}H\}$ NMR (125.77 MHz, DMSO-d_6), and (**c**) HRMS (ESI).

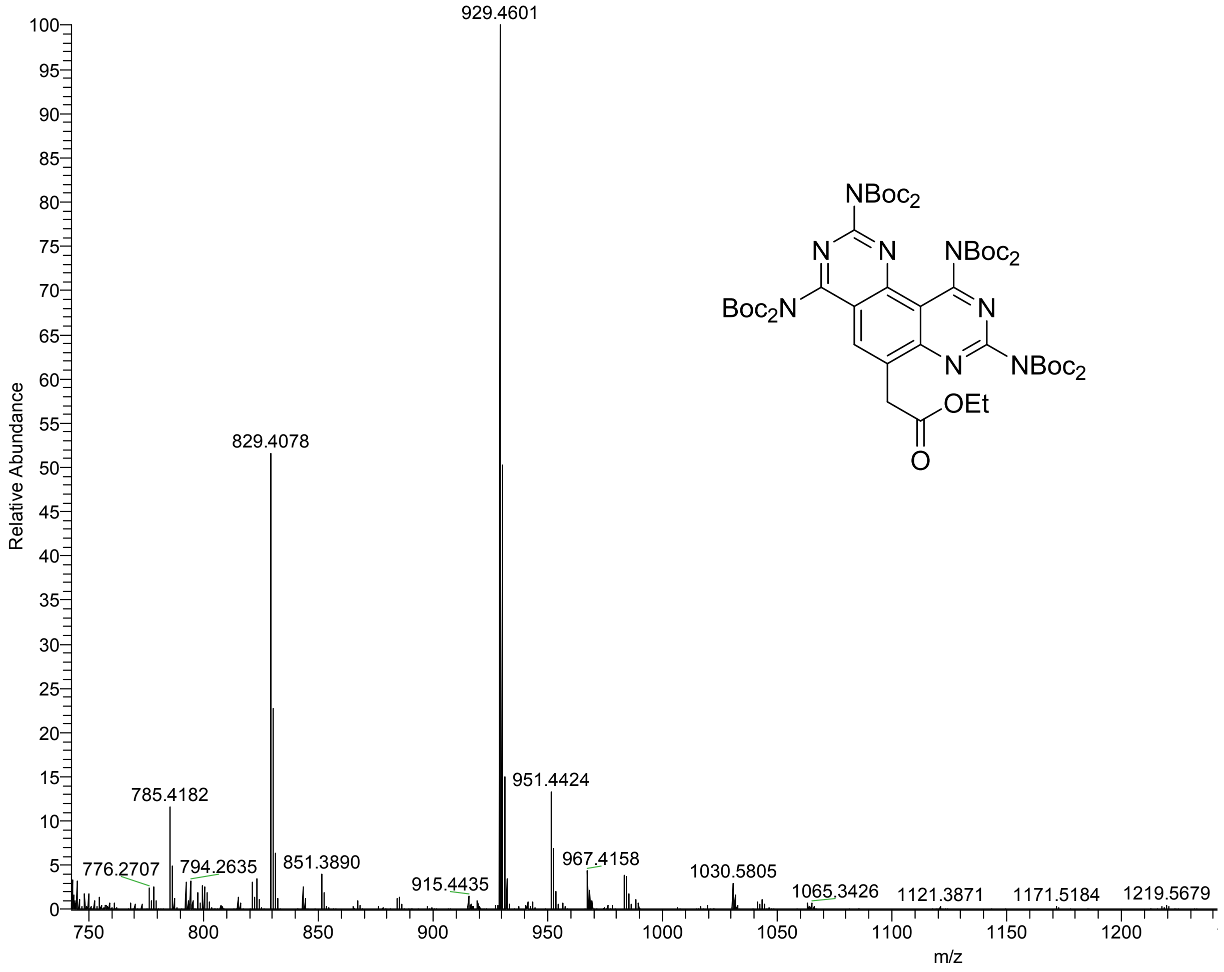

Figure S16. Spectroscopic data for compound **16**: HRMS (ESI).

(a)

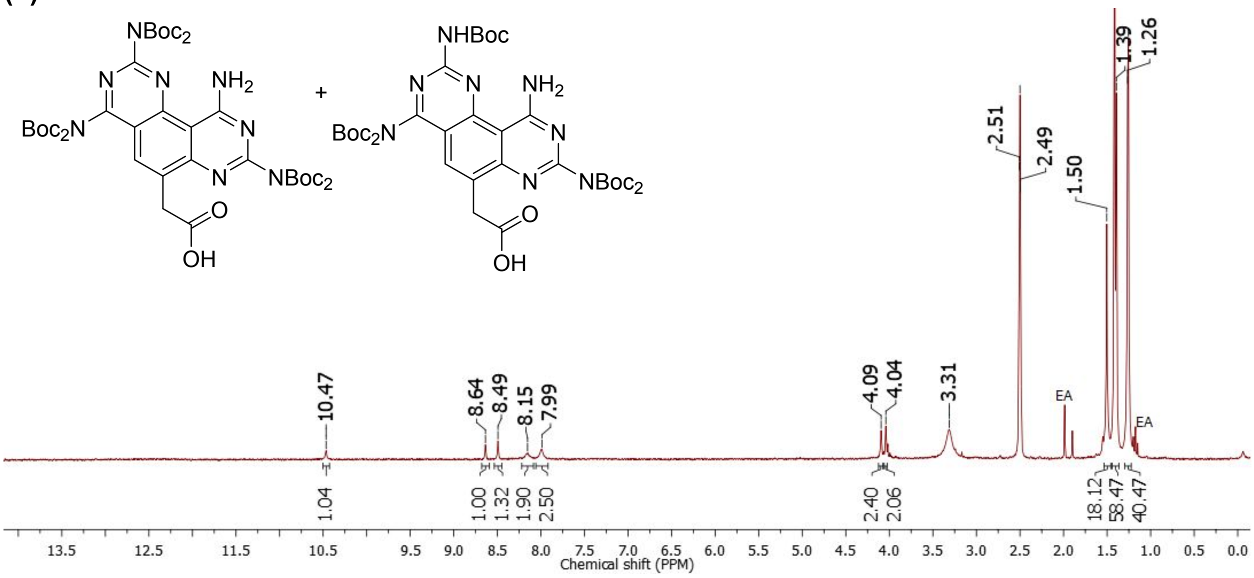

(b)

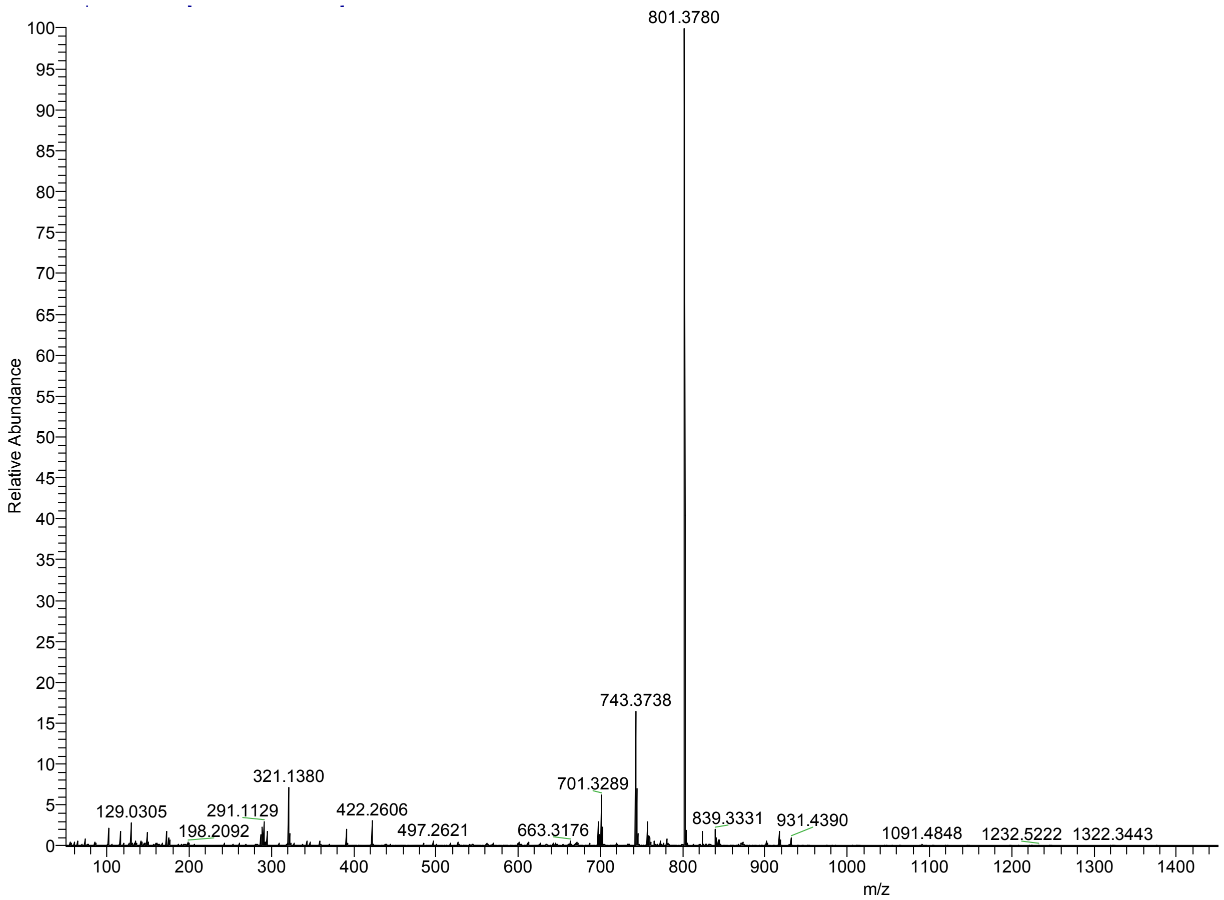

Figure S17. Spectroscopic data for compound **2/K**: (**a**) ^{1}H NMR (500.13 MHz, DMSO-d_6), and (**b**) HRMS (ESI).

(a)

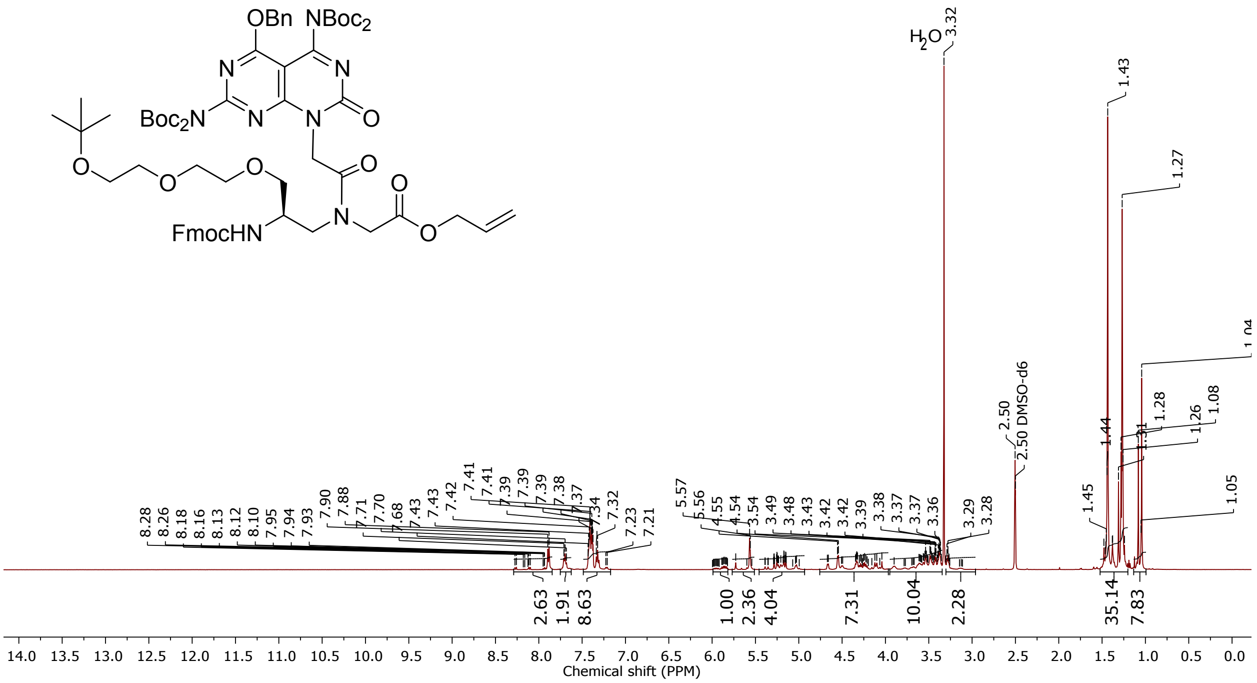

(b)

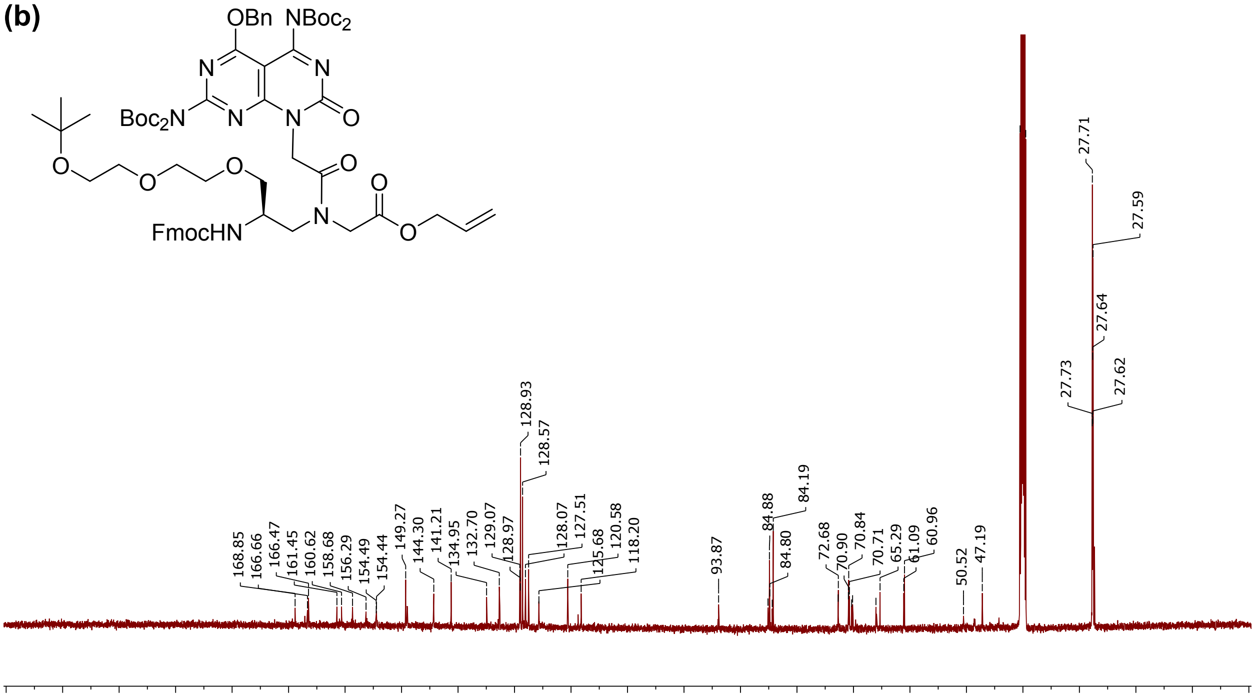

(c)

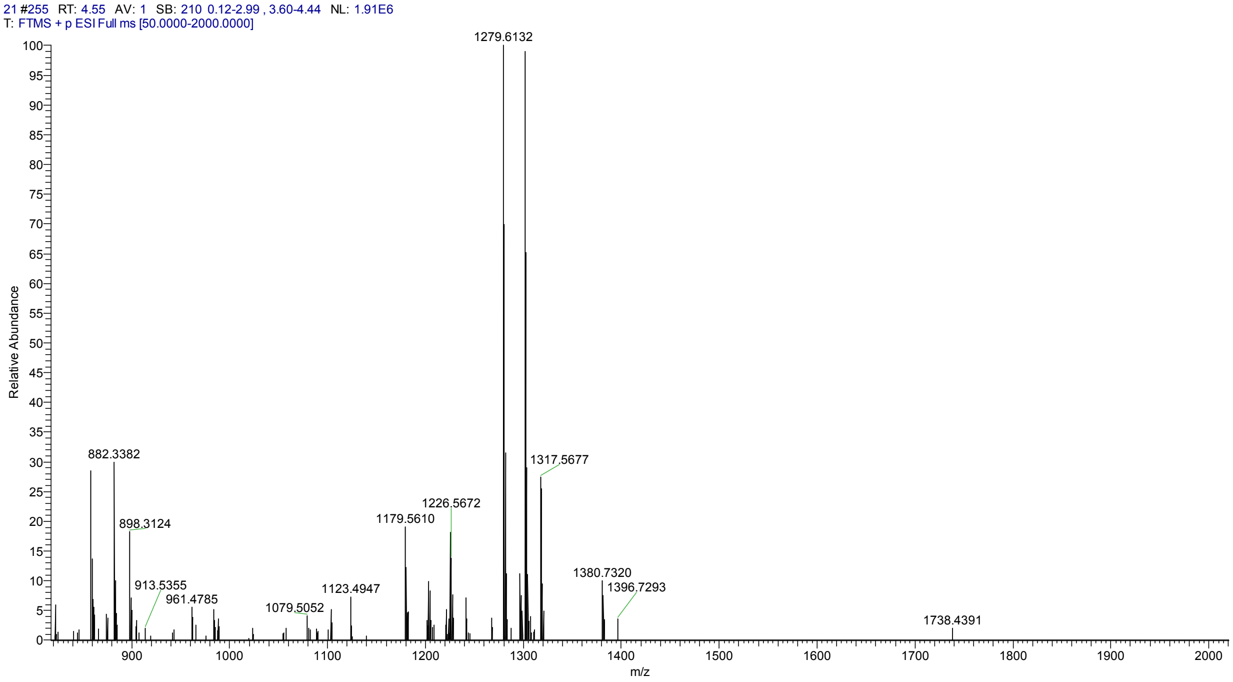

Figure S18. Spectroscopic data for compound **18a**: (**a**) ^{1}H NMR (500.13 MHz, DMSO-d_6), (**b**) $^{13}C\{^{1}H\}$ NMR (125.77 MHz, DMSO-d_6), and (**c**) HRMS (ESI).

(a)

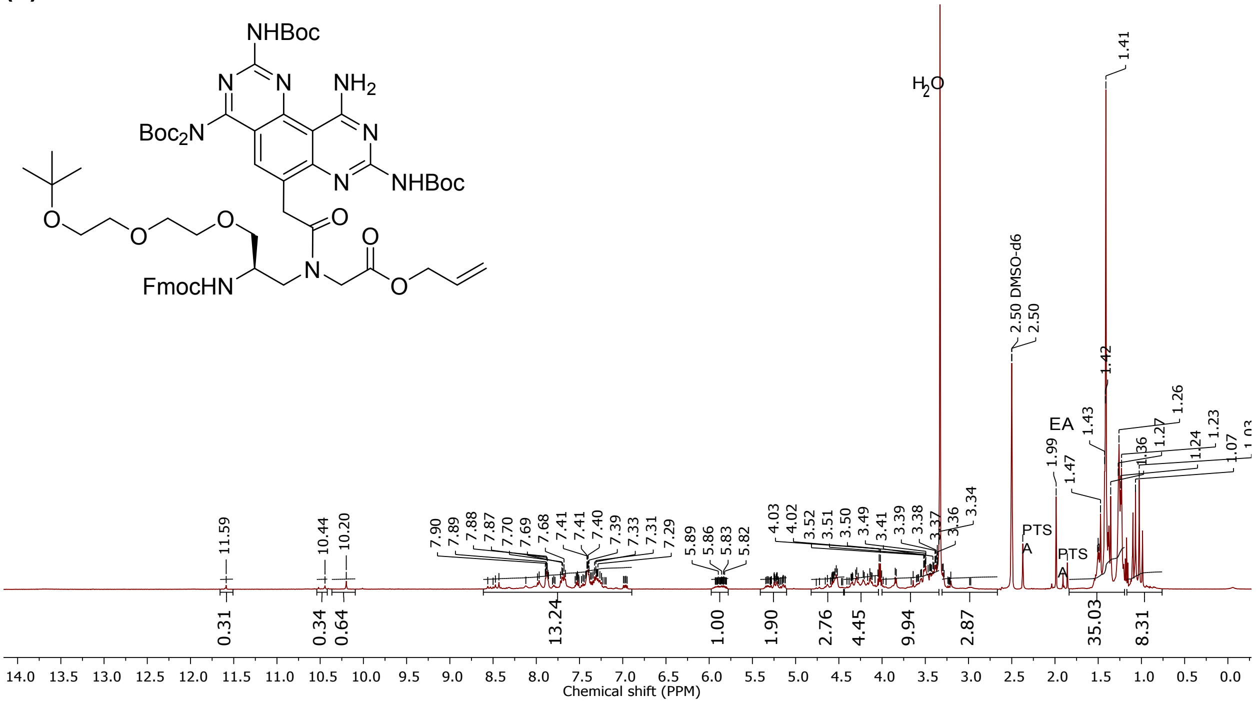
NHBoc
NH2
Boc2N
NHBoc
FmocHN
H2O
2.50 DMSO-d6
EA
PTSA
Chemical shift (PPM)

(b)

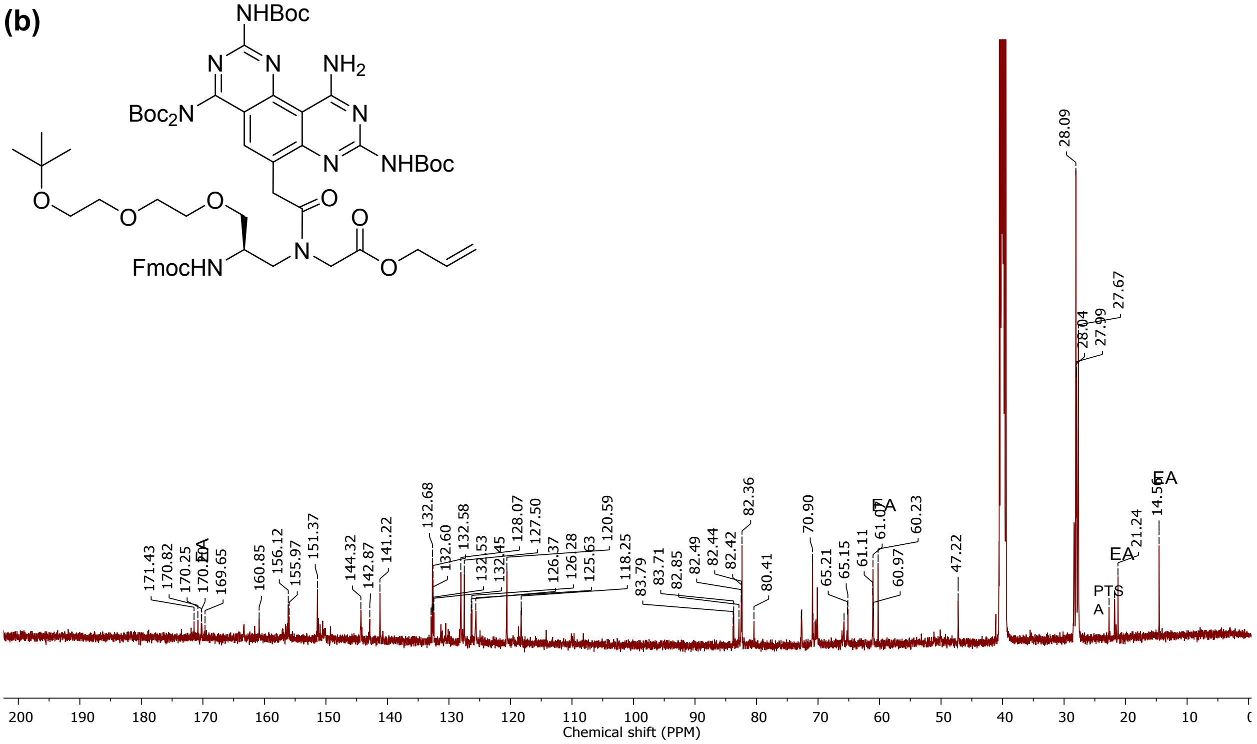
NHBoc
NH2
Boc2N
NHBoc
FmocHN
EA
PTSA
Chemical shift (PPM)

(c)

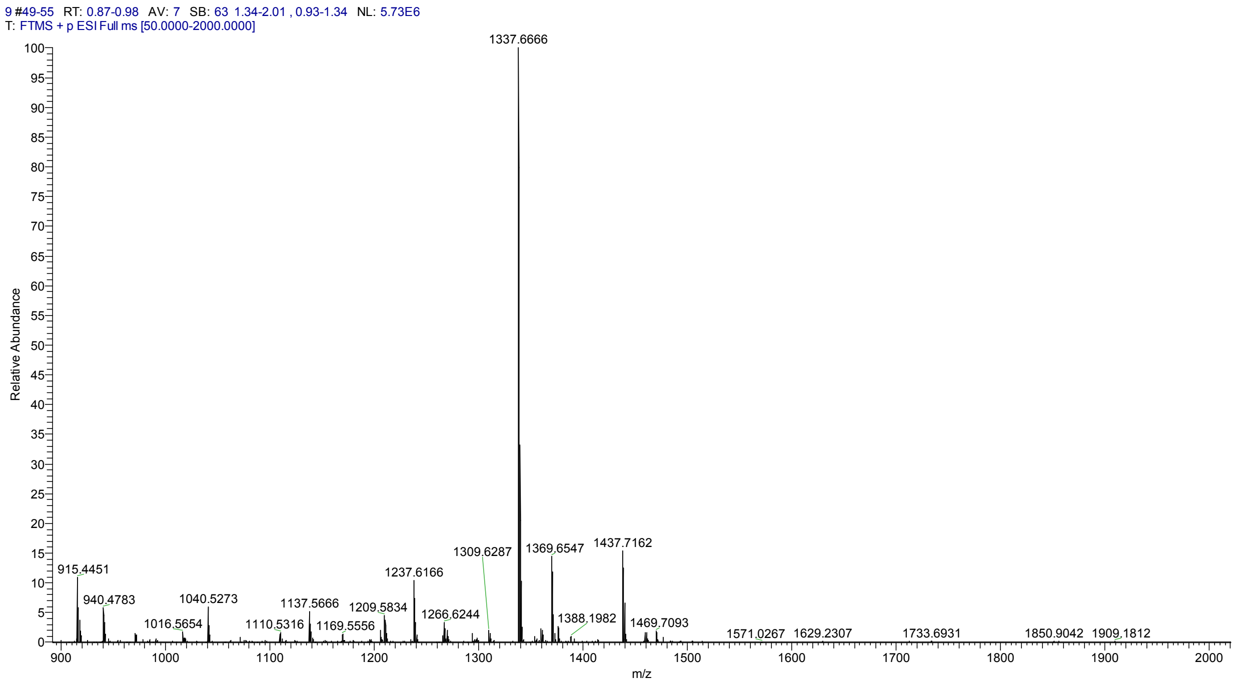

Figure S19. Spectroscopic data for compound **18b:** (**a**) ^{1}H NMR (500.13 MHz, DMSO-d_6), (**b**) $^{13}C\{^{1}H\}$ NMR (125.77 MHz, DMSO-d_6), and (**c**) HRMS (ESI).

(a)

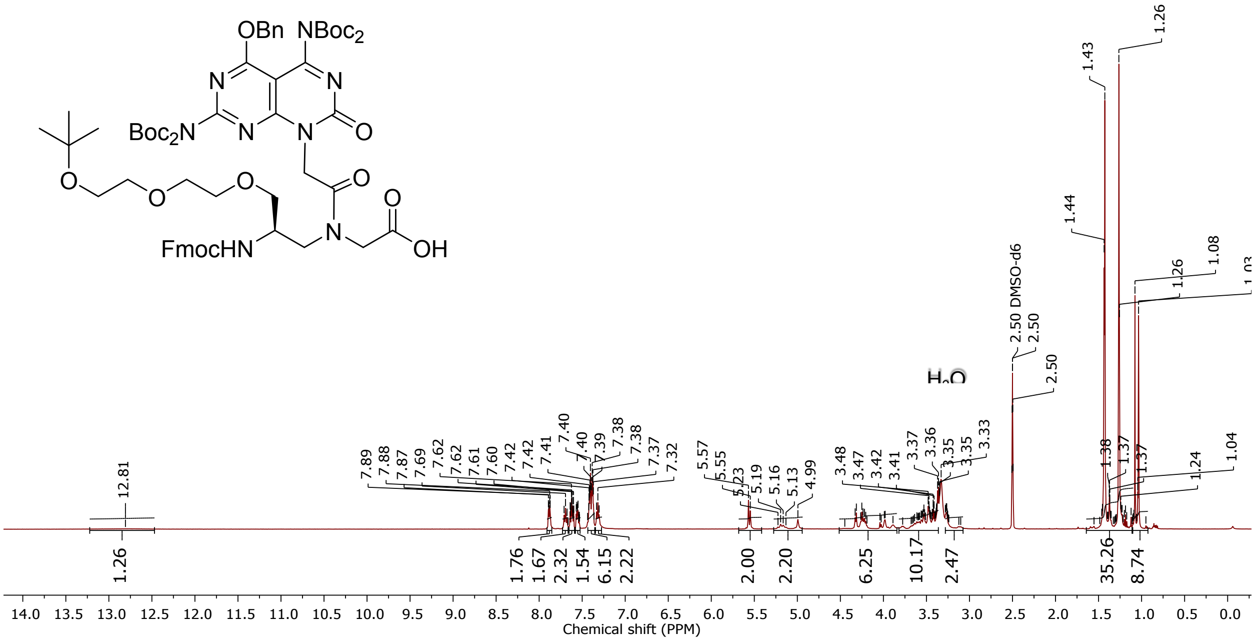

OBn
NBoc2
Boc2N
FmocHN
OH
H2O
2.50 DMSO-d6
Chemical shift (PPM)

(b)

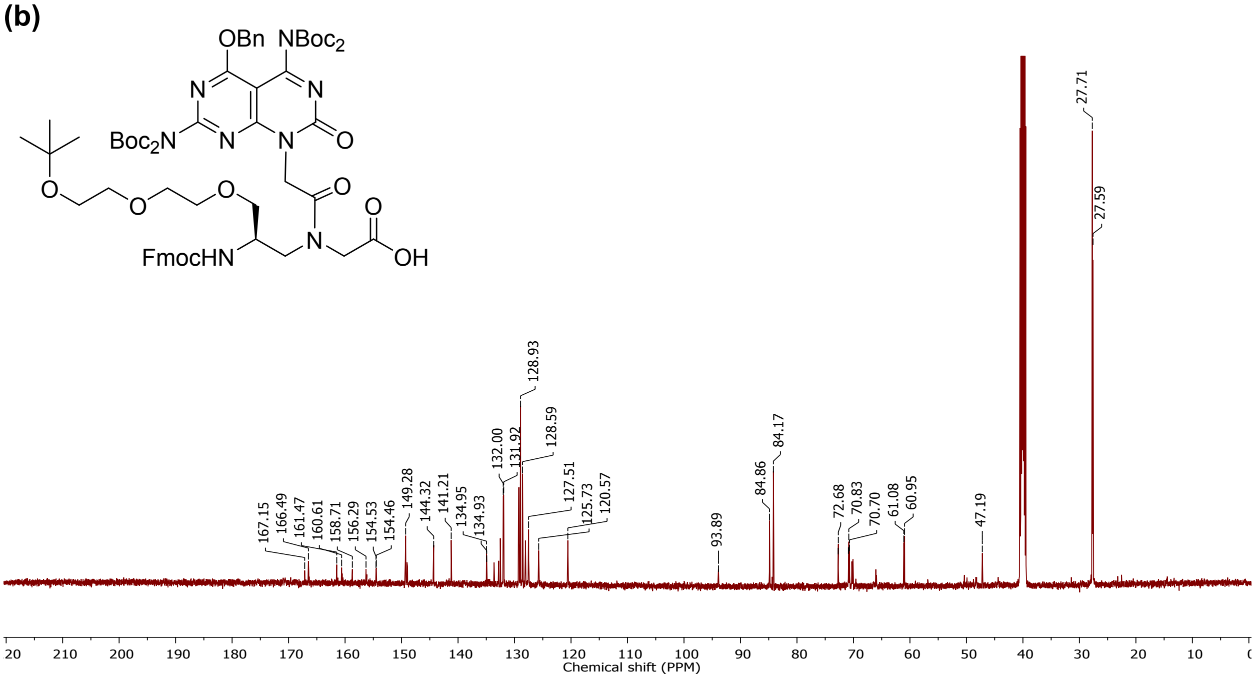

OBn
NBoc2
Boc2N
FmocHN
OH
Chemical shift (PPM)

(c)

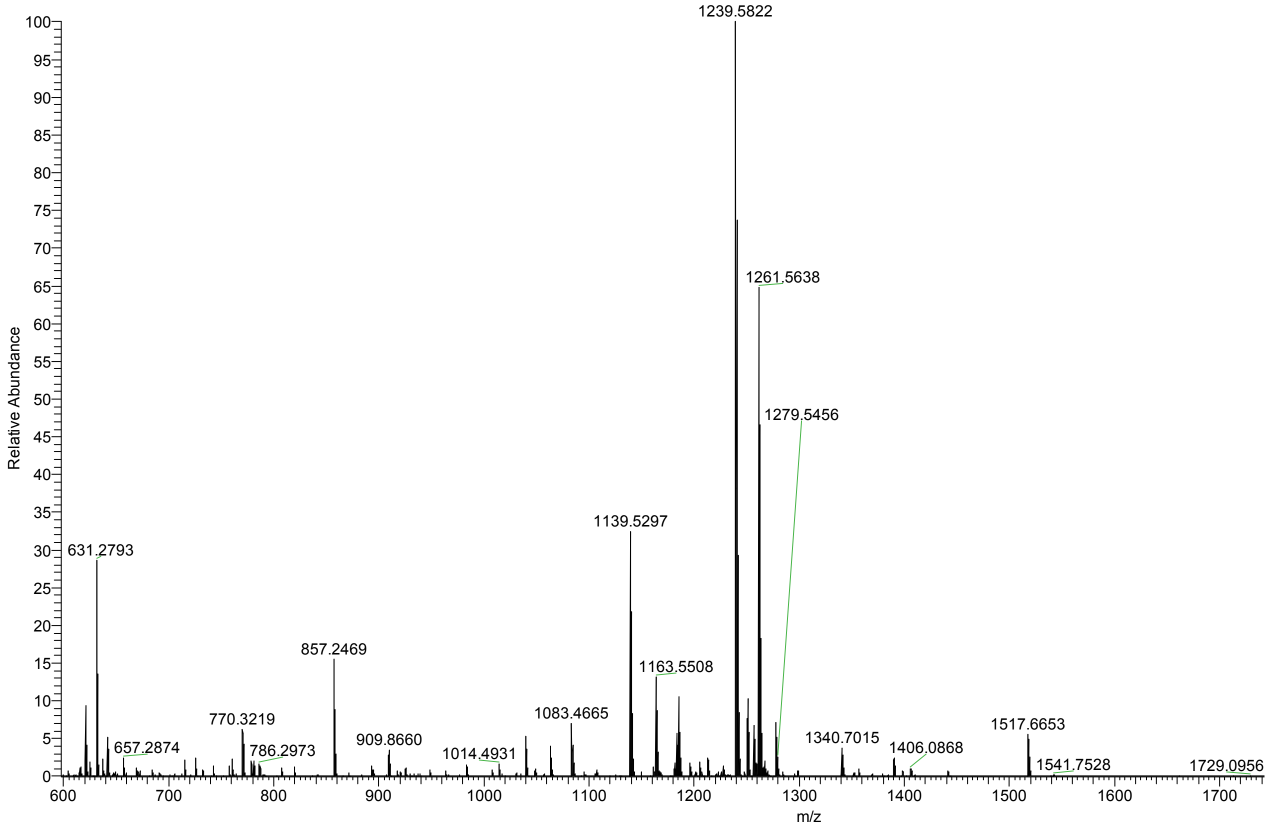

Figure S20. Spectroscopic data for compound **19**: (**a**) ^{1}H NMR (500.13 MHz, DMSO-d_6), (**b**) ^{13}C{^{1}H} NMR (125.77 MHz, DMSO-d_6), and (**c**) HRMS (ESI).

(a)

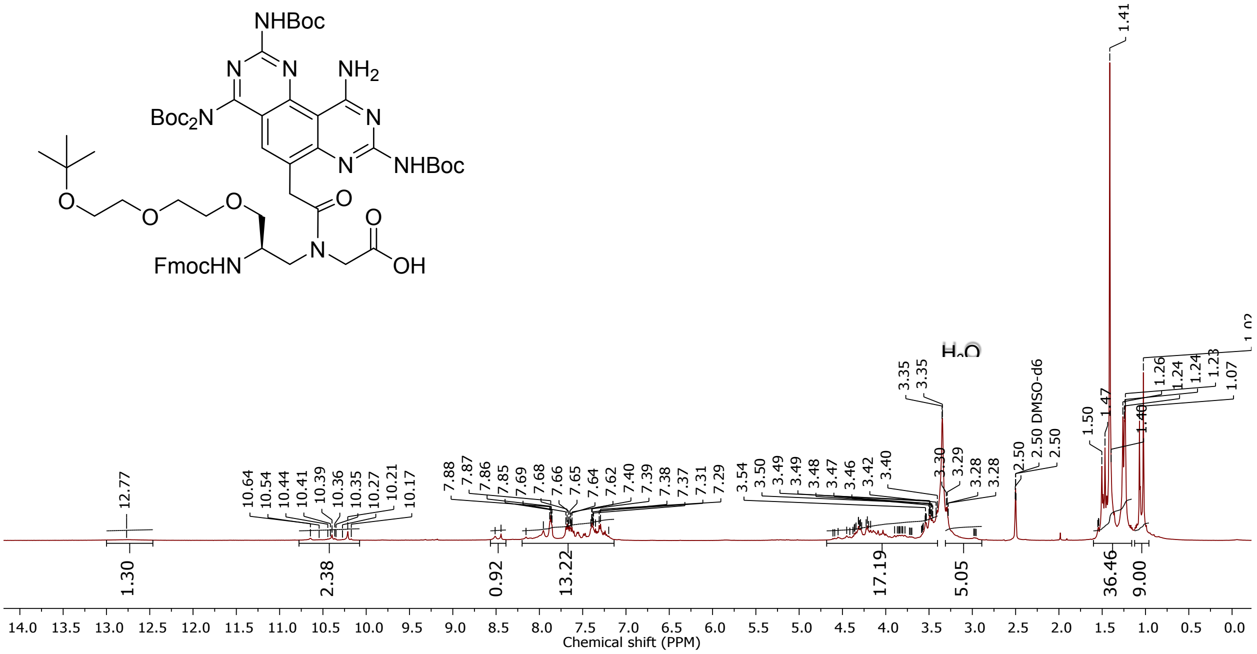
NHBoc
NH2
Boc2N
NHBoc
FmocHN
OH
H2O
DMSO-d6
Chemical shift (PPM)

(b)

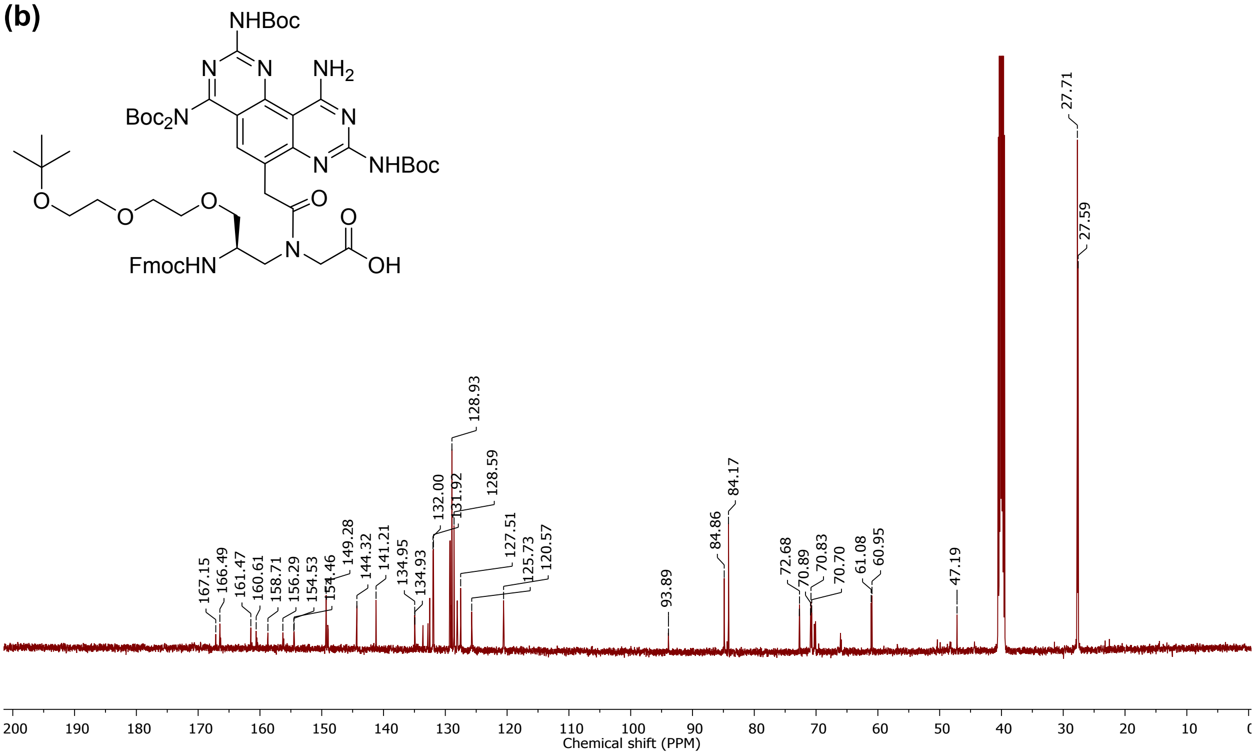
NHBoc
NH2
Boc2N
NHBoc
FmocHN
OH
Chemical shift (PPM)

(c)

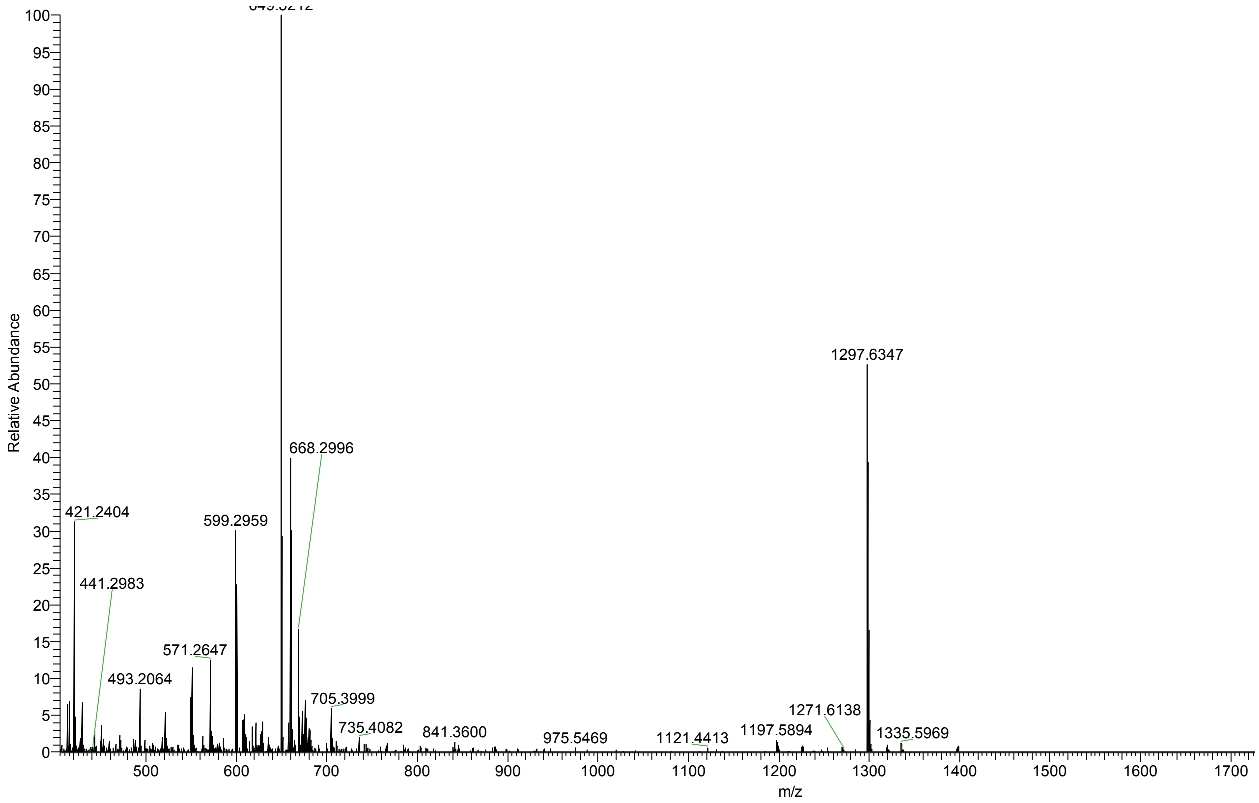

Figure S21. Spectroscopic data for compound **20**: (**a**) ^{1}H NMR (500.13 MHz, DMSO-d_6), (**b**) $^{13}C\{^{1}H\}$ NMR (125.77 MHz, DMSO-d_6), and (**c**) HRMS (ESI).
